# Diversity distribution patterns of Chinese endemic seed plant species and their implications for conservation planning

**DOI:** 10.1038/srep33913

**Published:** 2016-09-23

**Authors:** Jihong Huang, Jianhua Huang, Xinghui Lu, Keping Ma

**Affiliations:** 1 Key Laboratory of Forest Ecology and Environment, the State Forestry Administration, Institute of Forest Ecology, Environment and Protection, Chinese Academy of Forestry, Beijing 100091, China; 2 Co-Innovation Center for Sustainable Forestry in Southern China, Nanjing Forestry University, Nanjing 210037, China; 3 State Key Laboratory of Vegetation and Environmental Change, Institute of Botany, Chinese Academy of Sciences, Beijing 100093, China; 4 School of Economics, Minzu University of China, Beijing 100081, China

## Abstract

Endemism is an important concept in biogeography and biodiversity conservation. China is one of the richest countries in biodiversity, with very high levels of plant endemism. In this study, we analysed the distribution patterns of diversity, the degree of differentiation, and the endemicity of Chinese endemic seed plants using the floristic unit as a basic spatial analysis unit and 11 indices. The analysis was based on distribution data of 24,951 native seed plant species (excluding subspecies and varieties) and 12,980 Chinese endemic seed plant species, which were sourced from both specimen records and published references. The distribution patterns of Chinese endemic flora were generally consistent but disproportionate across China for diversity, degree of differentiation and endemicity. The South Hengduan Mountains Subregion had the highest values for all indices. At the regional level, both the Hengduan Mountains and the Central China regions were highest in diversity and degrees of differentiation. However, both the rate of local endemic to native species and the rate of local to Chinese endemic species were highest in the Taiwan Region and the South Taiwan Region. The Hengduan Mountains Region and the Central China Region are two key conservation priority areas for Chinese endemic seed plants.

The Chinese flora has long attracted the attention of botanists and biogeographers^1^,^2^,^3^,^4^,^5^,^6^,^7^,^8^. China is one of the richest countries in terms of plant biodiversity^9^,^10^. The total number of vascular plant species in China is 31362 (Wu *et al*.^11^). The Chinese flora is also highly endemic^12^,^13^,^14^; a previous study found that endemic species account for 52.1% of all seed plant species^8^. China is also the only country in the world that supports vegetational continuity from tropical, to subtropical, temperate and boreal forests^2^,^15^. This continuous latitudinal gradient of forest vegetation, in combination with the many mountain ranges in China, presumably reduced rates of extinction during glaciations and increased rates of evolution and speciation for vascular plants^2^. China has attracted the attention of ecologists and conservationists because of the extraordinary richness of its flora^16^,^17^,^18^,^19^,^20^ and because it is among the countries with the highest numbers of threatened species in the world (Jenkins *et al*.^21^). Of the World Wildlife Fund’s Global 200 most Critical and Endangered Ecoregions, 17 are located in or intersect with China (Olson & Dinerstein^22^). Furthermore, of the 34 global biodiversity hotspots identified by Conservation International (Mittermeier *et al*.^23^), four either intersect with or are located within China. In total, 10 hotspot ecoregions^24^, 20 hotspots of endemic woody seed plant species^20^ and 8 hotspots of threatened plant species^25^ have been identified in China. By the end of 2014, China had established 2729 nature reserves^26^, but many were established opportunistically and do not fully represent and protect biodiversity priority areas^27^ or threatened plants^28^.

The flora in China is extremely rich and complex in species composition and distribution patterns. Thus, the regionalization of Chinese flora is also difficult and challenging. Wu was the first researcher to publish a comprehensive regionalization of Chinese flora based on pioneering works that had been published over decades. He classified Chinese flora into 2 kingdoms, 7 subkingdoms and 22 regions^29^. Subsequently, the regionalization of Chinese flora was updated and refined into 4 kingdoms, 7 subkingdoms, 24 regions and 49 subregions in total^1^.

As described previously, many studies of biodiversity conservation have been conducted across China. However, they primarily used an administrative unit, particularly at the provincial level, as the spatial unit for analysis rather than a natural geographic or floristic unit. It is better to identify conservation priority areas on the basis of a natural geographic or floristic unit because such classifications substantially reduce the influence of and definition by human factors. Therefore, using the floristic unit as the basic unit of spatial analysis, we examined the distribution patterns, diversification and degrees of differentiation of Chinese endemic seed plants and identified conservation priority areas across the country for conservation planning and future effective conservation actions.

## Materials and Methods

### Species data

Based on a previously published list of Chinese endemic seed plant species^16^, we compiled a data set of species distributions of Chinese endemic seed plants at the county level. The main sources used to compile the distribution dataset were as follows: (a) Flora of China^11^ and Flora Reipublicae Popularis Sinicae^30^; (b) local flora, plant checklists and relevant monographs; (c) journal papers on plant taxonomy and distribution; and (d) specimens. Our research sources included 1044 flora, monographs, reports and theses; 516 papers; and 37 herbaria through the end of 2012. The references and herbaria are listed in Appendix A. The distribution data were extracted from the source data and compiled and unified to county level. Then, a geographic distribution database of Chinese endemic seed plant species was established. We compiled 789,004 records of 24,951 native seed plant species (excluding subspecies and varieties) with distribution information at the county level. Of these species, 12,980 (Appendix B) are endemic to China.

### Floristic regions of China

**Figure 1:**
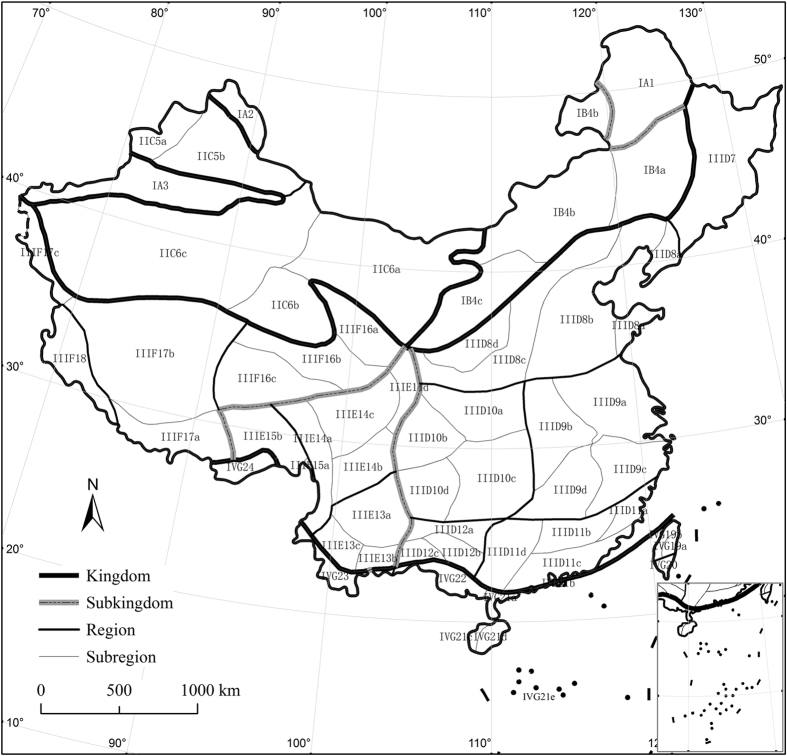
Distribution of floristic regionalization of China. Albers projection. The subregion codes on the map are consistent with those provided in Table 1. The map was generated using ArcGIS 9.3 (ESRI, Redlands, CA, USA; http://www.esri.com).

We digitized the map of floristic regions of China^1^ using ArcGIS 9.3^31^. The floristic regions of China were composed of 4 kingdoms, 7 subkingdoms, 24 regions and 49 subregions (Table 1 and Fig. 1). There are 9 regions without subregions. We used the floristic subregion as the basic unit for spatial analysis in this study. Thus, 58 spatial floristic units were examined (i.e., 49 subregions and 9 regions) (Table 1 and Fig. 1). Two of the 58 units — the Islands of East Guangdong along sea Subregion (IVG21b) and the Islands of South China Sea Subregion (IVG21e) — comprise multiple islands. We were unable to generate a checklist of plant species for these two units because high-precision species distribution data for them were not available. Therefore, 56 spatial floristic units were generated. We extracted centroids (latitude and longitude coordinates) for all counties using Data Management Tools in ArcGIS. These centroids were incorporated into 56 floristic units with Analysis Tools, and records of the floristic units for each species were then obtained. Distribution information regarding the presence or absence of each species was documented for each floristic unit.

### Data analysis

Measurement of species diversity was based on species richness, i.e., the number of species in each spatial unit^32^. Family diversity and genus diversity were calculated using family richness and genus richness, respectively, i.e., the number of families and genera in each spatial unit. In addition, the local endemic species (NLES) for each floristic unit were counted according to their distributions.

The degrees of differentiation among endemic flora were represented by differentiation indices, which included a species differentiation index (D_s_)^33^, a genus differentiation index (D_g_)^33^ and a species–family differentiation index (D_sf_)^34^. The functions of the three indices are shown in Equations (1, 2, 3).


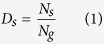



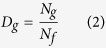



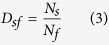


where *D*_*s*_, *D*_*g*_ and *D*_*sf*_ are the species differentiation index, genus differentiation index and species–family differentiation index for a floristic unit, respectively, and *N*_*s*_ is the number of species endemic in China in a floristic unit, *N*_*g*_ is the number of genera of endemic flora in China in a floristic unit and *N*_*f*_ is the number of families of endemic flora in China in a floristic unit.

Biodiversity conservation is closely associated with species distribution and biogeography. Generally, species that are confined to a limited area have an increased risk of extinction. Here, we calculated the weighted endemism^35^,^36^ for each floristic unit. The calculation of weighted endemism is shown in Equation (4).


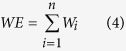


where *n* is the number of species in a focal floristic unit and *W*_*i*_ is the weight of species *i*, which is the inverse of its range (i.e., the number of floristic units in which species *i* occurs).

Proportions of endemism were calculated to compare the distribution patterns of endemic flora in China. These proportions included the rate of Chinese endemic to native species (RCENS), the rate of local endemic to native species (RLENS) and the rate of local to Chinese endemic species (RLCES).

To summarize, the 11 indices were as follow: the number of species (N_s_), the number of genera (N_g_), the number of families (N_f_), the number of local endemic species (NLES), the degree of species differentiation (*D*_*s*_), the degree of genus differentiation (*D*_*g*_), the degree of species–family differentiation (*D*_*sf*_), weighted endemism (WE), the rate of Chinese endemic to native species (RCENS), the rate of local endemic to native species (RLENS) and the rate of local to Chinese endemic species (RLCES). These 11 indices were standardized by dividing their maxima and then summed to produce a total value. All floristic units were ranked according to their total value, and conservation priority areas for Chinese endemic seed plants were identified according to each unit’s rank. The spatial floristic unit was used to prioritize conservation areas, and the units with the highest total values were the tip-ranked areas because higher values were determined by greater endemic species richness and the degree of differentiation and endemism. In this study, the top eight floristic units were designated conservation priority areas.

Correlations between the values of indices and the geographic distance from focal floristic units to the centre floristic unit with the highest value for each index were identified using the Mantel test^37^. All calculations were carried out using the R statistical software package, version 3.2.2^38^. All geographic distribution patterns of Chinese endemic flora were mapped using ArcGIS 9.3 software.

## Results

### Composition and distribution of Chinese endemic seed plant species

**Figure 2:**
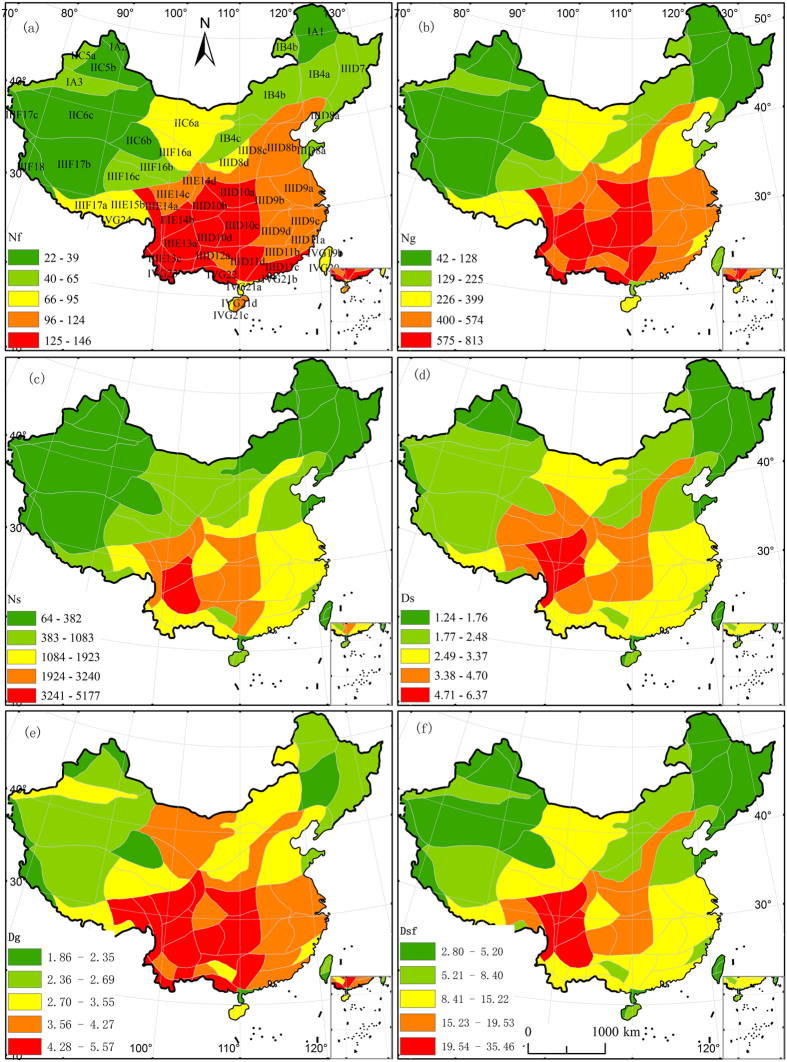
Distribution of biodiversity and differentiation indices across Chinese floristic units. (**a**) N_f_ =  number of families of Chinese endemic seed plants, (**b**) N_g_ = number of genera of Chinese endemic seed plants, (**c**) N_s_ = number of species of Chinese endemic seed plants, (**d**) D_s_ = degree of species differentiation among Chinese endemic seed plants, (**e**) D_g_ = degree of genus differentiation among Chinese endemic seed plants, and (**f**) D_sf_ = degree of species–family differentiation of Chinese endemic seed plants. Five levels for each index were categorized using the Natural Breaks (Jenks) method in ArcGIS. Albers projection. The subregion codes on the map are consistent with those provided in Table 1. The map was generated using ArcGIS 9.3 (ESRI, Redlands, CA, USA; http://www.esri.com).

Families and genera of Chinese endemic seed plant species were unevenly distributed across the country. At the family level, the South Hengduan Mountains Subregion (IIIE14b) and the Central Yunnan Plateau Subregion (IIIE13a) contained the most families (Table 2 and Fig. 2a). These two subregions were followed by the Southeast Yunnan Subregion (IIIE13b), the Guizhou-Guangxi Border Subregion (IIID12a) and the Guizhou Plateau Subregion (IIIE13b). The South Hengduan Mountains Subregion (IIIE14b) and the Central Yunnan Plateau Subregion (IIIE13a) were the richest in genera of Chinese endemic seed plant species, with 813 and 785 genera, respectively (Table 2). These subregions were followed by the Guizhou Plateau Subregion (IIID10d), the Yunnan-Myanmar-Thailand Border Region (IVG23), the Sanjiang Valley Subregion (IIIE14a), the Guizhou-Guangxi Border Subregion (IIID12a), the Southeast Yunnan Subregion (IIIE13b), the Guangdong-Guangxi Mountain Subregion (IIID11d), the Sichuan-Hubei-Hunan Border Subregion (IIID10c) and the Beibu Gulf Region (IVG22) (Table 2 and Fig. 2b).

Chinese endemic seed plant species were also unevenly distributed across the country. The South Hengduan Mountains Subregion (IIIE14b) was the richest, with 5177 species of Chinese endemic seed plants, which was followed by the Central Yunnan Plateau Subregion (IIIE13a), with 3688 species (Table 2). The Pamir-Karakoram-Kunlun Subregion was the least rich in Chinese endemic species richness and has only 64 species (Table 2). At the region level, the distribution of Chinese endemic seed plants was concentrated mainly in the Hengduan Mountains Region (IIIE14), followed by the Yunnan Plateau Region (IIIE13), the Yunnan-Guizhou-Guangxi Border Region (IIID12), the Lingnan Mountains Region (IIID11), the Central China Region (IIID10), the Yunnan-Myanmar-Thailand Border Region (IVG23) and the Beibu Gulf Region (IVG22) (Fig. 2c).

The distribution patterns of Chinese endemic flora at family, genus and species levels across the country were generally consistent (Fig. 2a–c). The patterns were significantly correlated (P < 0.001), and all correlation coefficients were greater than 0.80. The South Hengduan Mountains Subregion (IIIE14b) and the Central Yunnan Plateau Subregion (IIIE13a), which bordered each other, were centres of Chinese endemic flora because they had the highest numbers of endemic taxa. The number of families, genera and species of Chinese endemic seed flora decreased with increasing distance from focal floristic units to the centre floristic unit with the highest value for each index (Fig. 2a–c), but the number of families and genera decreased significantly (P < 0.05).

### Species differentiation

The degree of differentiation among endemic seed plant species was unevenly distributed across China (Fig. 2d–f). The highest species differentiation was in the South Hengduan Mountains Subregion (IIIE14b), with a value of 6.37, followed by the North Hengduan Mountains Subregion (IIIE14c), with a value of 5.56 (Table 2). The lowest degree of species differentiation was in the Tacheng and Yili Subregion, with a value of 1.24 (Table 2). The genus differentiation among Chinese endemic seed plant species was the highest (5.57) in the South Hengduan Mountains Subregion (IIIE14b) and the lowest (1.86) in the South Taiwan Region (IVG20). The pattern for the species–family differentiation index was similar to that observed for genus differentiation (Table 2).

### Local endemic species and their endemicity

**Figure 3:**
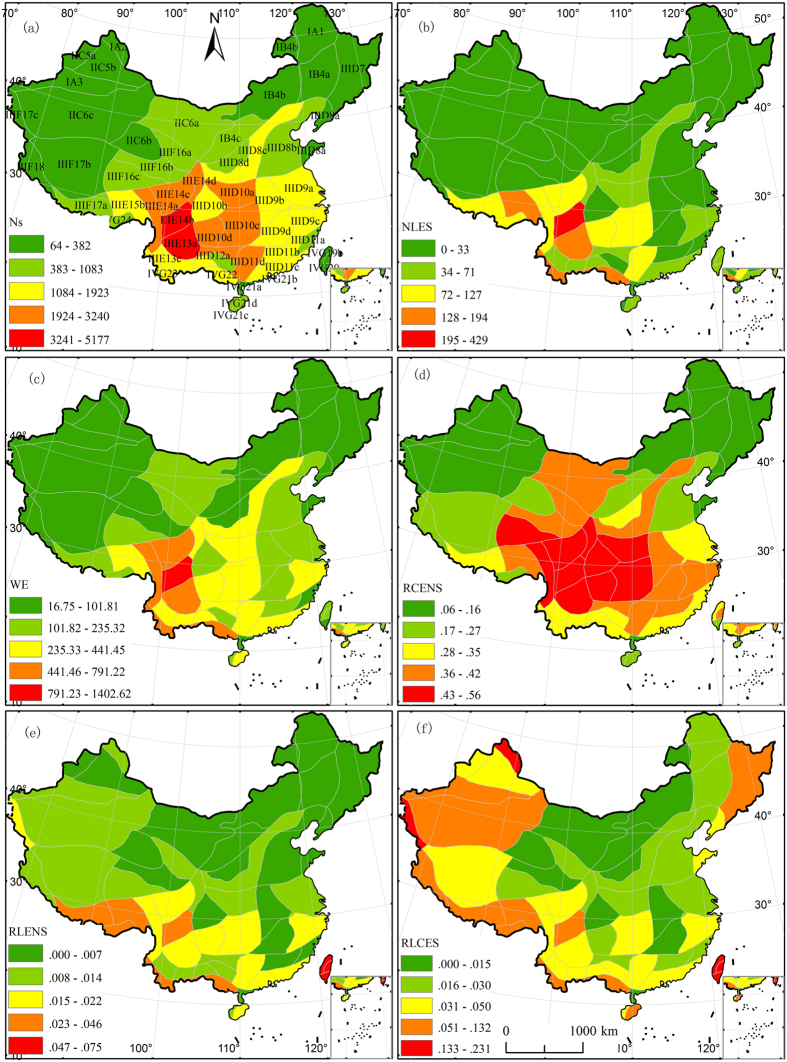
Distribution of endemic indices and endemic rates cross Chinese floristic unit. (**a**) N_s _= number of species of Chinese endemic seed plants, (**b**) NLES = number of local endemic species, (**c**) WE = weighted endemism, (**d**) RCENS = rate of Chinese endemic to native species, (**e**) RLENS = rate of local endemic to native species, and (**f**) RLCES = rate of local to Chinese endemic species. Five levels for each index were categorized using the Natural Breaks (Jenks) method in ArcGIS. Albers projection. The subregion codes on the map are consistent with those used in Table 1. The map was generated using ArcGIS 9.3 (ESRI, Redlands, CA, USA; http://www.esri.com).

The spatial distribution patterns of Chinese endemic plant species, local endemic species and weighted endemism were significantly correlated (P < 0.001) (Fig. 3a–c), and all correlation coefficients were greater than 0.75. The South Hengduan Mountains Subregion (IIIE14b) was the richest in local endemic species, with a value of 429. The Yunnan-Myanmar-Thailand Border Subregion ranked second, with 194 local endemic species.

The endemic rates were also unevenly distributed across the country (Fig. 3d–f). The rate of Chinese endemic to native plant species (RCENS) was highest in the South Hengduan Mountains Subregion (IIIE14b) and the North Hengduan Mountains Subregion (IIIE14c). Both the rate of local endemic to native species (RLENS) and the rate of local to Chinese endemic species (RLCES) were the highest in the Taiwan Region (VG19) and the South Taiwan Region (IVG20). The rate of local to Chinese endemic species (RLCES) was also very high in the Altai Region (IA2) and the Pamir-Karakoram-Kunlun Subregion (IIIF17c), followed by the South Taiwan Region (IVG20).

### Priority areas of floristic regions for Chinese endemic seed plant species

**Figure 4:**
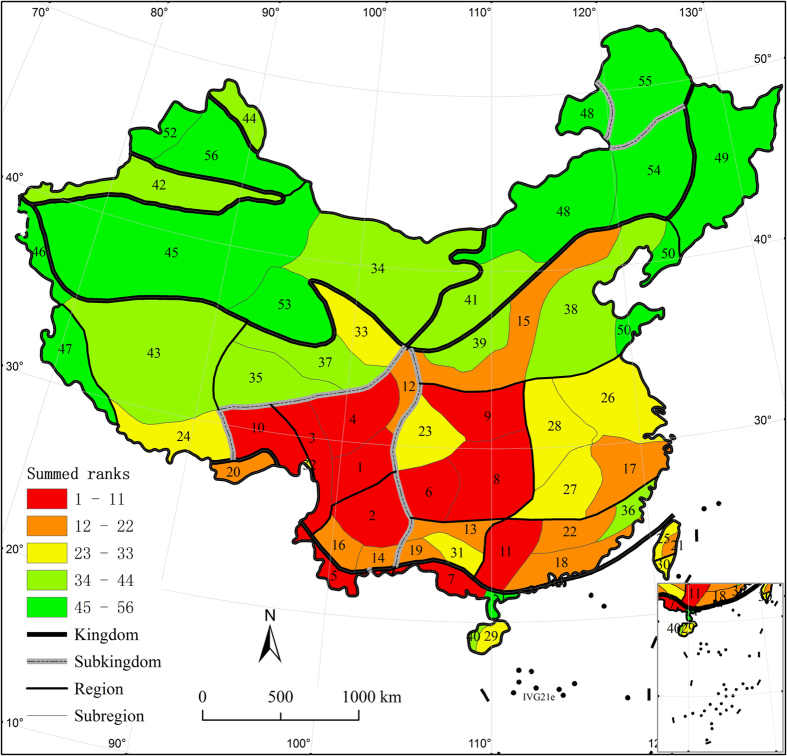
Distribution of the summed rank of all 11 indices cross Chinese floristic unit. Five levels for the summed rank were categorized using the Natural Breaks (Jenks) method in ArcGIS. Albers projection. Arabic numbers on the map indicate the rank. The map was generated using ArcGIS 9.3 (ESRI, Redlands, CA, USA; http://www.esri.com).

The South Hengduan Mountains Subregion (IIIE14b), the Central Yunnan Plateau Subregion (IIIE13a), the Sanjiang Valley Subregion (IIIE14a) and the North Hengduan Mountains Subregion (IIIE14c) had the highest total values of all floristic units (Fig. 4). These subregions, with the exception of the Central Yunnan Plateau Subregion (IIIE13a), are in the geographic space of the Hengduan Mountains Region (Table 1). The subregions that followed those with the highest values were the Guizhou Plateau Subregion (IIID10d), the Sichuan-Hubei-Hunan Border Subregion (IIID10c), the Qinling-Bashan Subregion (IIID10a), and the Taohe-Minshan Subregion (IIIE14d). Of these regions, the first three are in the Central China Region (Table 1), and the Taohe-Minshan Subregion is also in the Hengduan Mountains Region (Table 1).

## Discussion

### Species differentiation

The degree of species differentiation among Chinese endemic seed plants was unevenly distributed across the country. Although the degree of differentiation was not high in many parts of China, it was particularly high in the Hengduan Mountains^12^, which indicated that the Hengduan Mountains are the centre of differentiation for Chinese endemic seed plants^39^. This result is consistent with the hypothesis that Southwest China is the differentiation centre of the Chinese flora^1^,^2^,^7^. The Hengduan Mountains are the core part of Southwest China in terms of biological diversity and differentiation^2^. Therefore, the Hengduan Mountains are the centre of biological diversity and differentiation in China.

### Endemicity

The rate of Chinese endemic to native plant species was highest in the South Hengduan Mountains Subregion (IIIE14b), followed by the North Hengduan Mountains Subregion (IIIE14c). Based on this result, endemicity of flora in the Hengduan Mountains is very high across China. However, the South Hengduan Mountains Subregion and the North Hengduan Mountains Subregion differ in endemicity. The terrain of China from west to east forms a flight of three steps, commonly called the “Geomorphological Three Steps”. The First Step mainly encompasses the Qinghai-Tibet Plateau, north to the Kunlun, Aerhchin, and Qilian mountains and east to Min, Qionglai, Daxue and Hengduan mountains. The Second Step lies between the Hengduan Mountains to the west and the Daxing’anling, Taihang, Funiu, and Xuefeng mountains to the east, primarily including the Inner Mongolian Plateau, Loess Plateau, Qinling Mountains, Sichuan Basin, and Yun-Gui Plateau. The Third Step covers the entire area east from the Second Step, and includes the Northeast China Plain, North China Plain, Middle-lower Yangtze Plain, Jiangnan hills, Nanling Mountains, Guangdong and Guangxi hills, Zhejiang and Fujian hills as well as Taiwan and Hainan^20^. The floristic endemicity was higher in the south Hengduan Mountains than that in the North Hengduan Mountains Subregion, most likely because the Hengduan Mountains are topographically located along the border of the Second Step and the Third Step in China, and the warm, moist airflows in summer occur primarily in the South Hengduan Mountains Subregion and have only a limited influence if they arrive in the North Hengduan Mountains Subregion. By contrast, the North Hengduan Mountains Subregion is often influenced by dry and cold airflows. The altitude range is mainly between 2000 and 3000 m in the South Hengduan Mountains Subregion, which is the altitude characterized by a rapid increase in the rate of Chinese endemic seed plants^8^.

The rate of local endemic to native plant species was highest in the Taiwan Region (IVG19) and the South Taiwan Region (IVG20), which indicates that the flora of Taiwan is distinctive. The rate of local to national endemic plant species was the highest in the Taiwan Region (IVG19), the South Taiwan Region (IVG20), the Altai Region (IA2) and the Pamir-Karakoram-Kunlun Subregion (III17c). This result shows that the island of Taiwan, the Altai Region (IA2) and the Pamir-Karakoram-Kunlun Subregion (III17c) have different flora that might be influenced by trans-boundary flora from neighbouring countries.

### Conservation priority areas

The four top conservation priority areas were identified; the South Hengduan Mountains Subregion (IIIE14b), the Central Yunnan Plateau Subregion (IIIE13a), the Sanjiang Valley Subregion (IIIE14a) and the North Hengduan Mountains Subregion (IIIE14c). Most of these subregions are in the geographical terrain of the Hengduan Mountains. The Hengduan Mountains are rich in mountainous flora and are thus a key area for studies of the relationships among Laurasian, Gondwanian and Tethyan flora^40^. Vegetation with both horizontal and vertical replacement is markedly dominant in the Hengduan Mountains^41^,^42^. Moreover, new and old endemic plant species coexist, although the new endemic species are dominant in this region^1^,^3^. The flora of this region is divided into three geographical elements based on ancient geographical environments^43^: Boreal, Tethys and Gondwana. Studies of the trade-offs among these geographical elements over space and time are beneficial in clarifying the relationships between the development of flora and the movements of tectonic plates^1^. Conservation International listed the montane areas of Southwest China as one of the 34 global biodiversity hotspots^23^.

Three other top conservation priority areas were identified; the Guizhou Plateau Subregion (IIID10d), the Sichuan-Hubei-Hunan Border Subregion (IIID10c) and the Qinling-Bashan Subregion (IIID10a). These three subregions are in the Central China Region. The flora of this region comprises direct and nearly intact descendants of a Tertiary ancient flora^1^ for several reasons. First, the Ancient Central China region did not suffer transgression after Indosinian movement in the Triassic period^44^. Second, montane refuges that were widespread during the Pleistocene glacial period were less affected in this region. In the glacial periods throughout the glacial-interglacial cycles, some plants migrated between the north and south and between low and high elevations near the Hengduan Mountains^44^,^45^. Third, this region is completely surrounded by mountains and has an enclosed terrain that is complex and varied^44^. All of these factors are closely related to the preservation of old endemic species, and to the differentiation of new endemic species^1^,^12^,^46^. Therefore, we suggest that more attention should be paid to this region for its protection. In our study, the conservation priority areas identified for Chinese endemic seed plants were highly consistent with the key flora of China that were identified in previous studies^1^. In addition, in this study, conservation priority areas were identified for the first time by using a floristic unit as the basic unit of spatial analysis across China. With this approach, the artificial impact of the unit of spatial analysis on the distribution patterns of species was substantially weakened because the bounded floristic subregion depended much more on natural factors than on human factors. Consequently, determination of conservation priority areas for Chinese endemic seed plants of the China flora is more credible when a floristic unit is the basic unit for spatial analysis.

## Conclusion

The diversity and degrees of differentiation among Chinese endemic seed plants are unevenly distributed across the country. Both the Hengduan Mountains and Central China regions are diversity centres that also have the highest degrees of differentiation. Moreover, the Hengduan Mountains Region and the Central China Region are two key conservation priority areas for Chinese endemic seed plants based on diversity, degrees of differentiation and endemicity. In addition, the Taiwan Region, the Altai Region and the Pamir-Karakoram-Kunlun Subregion have unique endemic flora, and greater attention should be paid to the flora of these regions or subregions in future conservation planning and actions.

## Additional Information

**How to cite this article**: Huang, J. *et al*. Diversity distribution patterns of Chinese endemic seed plant species and their implications for conservation planning. *Sci. Rep*. **6**, 33913; doi: 10.1038/srep33913 (2016).

## Supplementary Material

Supplementary Information

Supplementary Appendix A

Supplementary Appendix B

## Figures and Tables

**Table 1:** Floristic regionalization of China.

Code	Subregion code	Subregion name	Kingdom code	Kingdom name	Subkingdom code	Subkingdom name	Region code	Region name
1	IA1	Da Hinggan Ling Region	I	Holarctic Kingdom	IA	Eurasian Forest Subkingdom	IA1	Da Hinggan Ling Region
2	IA2	Altai Region	I	Holarctic Kingdom	IA	Eurasian forest Subkingdom	IA2	Altai Region
3	IA3	Tianshan Region	I	Holarctic Kingdom	IA	Eurasian forest Subkingdom	IA3	Tianshan Region
4	IB4a	Northeast Plain forest steppe Subregion	I	Holarctic Kingdom	IB	Eurasian Steppe Subkingdom	IB4	Inner Mongolia Grassland Region
5	IB4b	Eastern Inner Mongolia grassland Subregion	I	Holarctic Kingdom	IB	Eurasian Steppe Subkingdom	IB4	Inner Mongolia Grassland Region
6	IB4c	Ordos, Shensi-Kansu-Ningia desert grassland Subregion	I	Holarctic Kingdom	IB	Eurasian Steppe Subkingdom	IB4	Inner Mongolia Grassland Region
7	IIC5a	Tacheng and Yili Subregion	II	Tethys Kingdom	IIC	Central Asia Desert Subkingdom	IIC5	Junggar Region
8	IIC5b	Junggar Subregion	II	Tethys Kingdom	IIC	Central Asia Desert Subkingdom	IIC5	Junggar Region
9	IIC6a	Southwest Inner Mongolia Subregion	II	Tethys Kingdom	IIC	Central Asia Desert Subkingdom	IIC6	Kashgar Region
10	IIC6b	Qaidam Basin Subregion	II	Tethys Kingdom	IIC	Central Asia Desert Subkingdom	IIC6	Kashgar Region
11	IIC6c	Kashi Subregion	II	Tethys Kingdom	IIC	Central Asia Desert Subkingdom	IIC6	Kashgar Region
12	IIID7	Northeast China Region	III	Eastern Asiatic Kingdom	IIID	China-Japan Forest Plant Subkingdom	IIID7	Northeast Region
13	IIID8a	Liaoning, Shandong Peninsula Subregion	III	Eastern Asiatic Kingdom	IIID	China-Japan Forest Plant Subkingdom	IIID8	North China Region
14	IIID8b	North China Plain Subregion	III	Eastern Asiatic Kingdom	IIID	China-Japan Forest Plant Subkingdom	IIID8	North China Region
15	IIID8c	North China Mountain Subregion	III	Eastern Asiatic Kingdom	IIID	China-Japan Forest Plant Subkingdom	IIID8	North China Region
16	IIID8d	The Loess Plateau Subregion	III	Eastern Asiatic Kingdom	IIID	China-Japan Forest Plant Subkingdom	IIID8	North China Region
17	IIID9a	Huanghuai Plain Subregion	III	Eastern Asiatic Kingdom	IIID	China-Japan Forest Plant Subkingdom	IIID9	East China Region
18	IIID9b	Jianghan Plain Subregion	III	Eastern Asiatic Kingdom	IIID	China-Japan Forest Plant Subkingdom	IIID9	East China Region
19	IIID9c	South Zhejiang Mountain Subregion	III	Eastern Asiatic Kingdom	IIID	China-Japan Forest Plant Subkingdom	IIID9	East China Region
20	IIID9d	South Jiangxi-East Hunan Hills Subregion	III	Eastern Asiatic Kingdom	IIID	China-Japan Forest Plant Subkingdom	IIID9	East China Region
21	IIID10a	Qinling-Bashan Subregion	III	Eastern Asiatic Kingdom	IIID	China-Japan Forest Plant Subkingdom	IIID10	Central China Region
22	IIID10b	Sichuan Pendi Subregion	III	Eastern Asiatic Kingdom	IIID	China-Japan Forest Plant Subkingdom	IIID10	Central China Region
23	IIID10c	Sichuan, Hubei and Hunan Border Subregion	III	Eastern Asiatic Kingdom	IIID	China-Japan Forest Plant Subkingdom	IIID10	Central China Region
24	IIID10d	Guizhou Plateau Subregion	III	Eastern Asiatic Kingdom	IIID	China-Japan Forest Plant Subkingdom	IIID10	Central China Region
25	IIID11a	North Fujian Mountain Subregion	III	Eastern Asiatic Kingdom	IIID	China-Japan Forest Plant Subkingdom	IIID11	Lingnan Mountains Region
26	IIID11b	North Guangdong Subregion	III	Eastern Asiatic Kingdom	IIID	China-Japan Forest Plant Subkingdom	IIID11	Lingnan Mountains Region
27	IIID11c	East Section of Nanling Subregion	III	Eastern Asiatic Kingdom	IIID	China-Japan Forest Plant Subkingdom	IIID11	Lingnan Mountains Region
28	IIID11d	Guangdong-Guangxi Mountain Subregion	III	Eastern Asiatic Kingdom	IIID	China-Japan Forest Plant Subkingdom	IIID11	Lingnan Mountains Region
29	IIID12a	Guizhou-Guangxi Border Subregion	III	Eastern Asiatic Kingdom	IIID	China-Japan Forest Plant Subkingdom	IIID12	Yunnan, Guizhou and Guangxi Region
30	IIID12b	Hongshuihe River Basin Subregion	III	Eastern Asiatic Kingdom	IIID	China-Japan Forest Plant Subkingdom	IIID12	Yunnan, Guizhou and Guangxi Region
31	IIID12c	Southeast Yunnan Limestone Subregion	III	Eastern Asiatic Kingdom	IIID	China-Japan Forest Plant Subkingdom	IIID12	Yunnan, Guizhou and Guangxi Region
32	IIIE13a	Central Yunnan Plateau Subregion	III	Eastern Asiatic Kingdom	IIIE	China-Himalayan Plant Subkingdom	IIIE13	Yunnan Plateau Region
33	IIIE13b	East Yunnan Subregion	III	Eastern Asiatic Kingdom	IIIE	China-Himalayan Plant Subkingdom	IIIE13	Yunnan Plateau Region
34	IIIE13c	Southwest Yunnan Subregion	III	Eastern Asiatic Kingdom	IIIE	China-Himalayan Plant Subkingdom	IIIE13	Yunnan Plateau Region
35	IIIE14a	Sanjiang valley Subregion	III	Eastern Asiatic Kingdom	IIIE	China-Himalayan Plant Subkingdom	IIIE14	Hengduan Mountains Region
36	IIIE14b	South Hengduan Mountains Subregion	III	Eastern Asiatic Kingdom	IIIE	China-Himalayan Plant Subkingdom	IIIE14	Hengduan Mountains Region
37	IIIE14c	North Hengduan Mountains Subregion	III	Eastern Asiatic Kingdom	IIIE	China-Himalayan Plant Subkingdom	IIIE14	Hengduan Mountains Region
38	IIIE14d	Taohe-Minshan Subregion	III	Eastern Asiatic Kingdom	IIIE	China-Himalayan Plant Subkingdom	IIIE14	Hengduan Mountains Region
39	IIIE15a	Dulongjiang River-North Myanmar Subregion	III	Eastern Asiatic Kingdom	IIIE	China-Himalayan Plant Subkingdom	IIIE15	East Himalayan Region
40	IIIE15b	Southeast Tibet Subregion	III	Eastern Asiatic Kingdom	IIIE	China-Himalayan Plant Subkingdom	IIIE15	East Himalayan Region
41	IIIF16a	Qilian Mountains Subregion	III	Eastern Asiatic Kingdom	IIIF	Qing-Tibet Plateau Subkingdom	IIIF16	Tanggute Region
42	IIIF16b	Animaqing Subregion	III	Eastern Asiatic Kingdom	IIIF	Qing-Tibet Plateau Subkingdom	IIIF16	Tanggute Region
43	IIIF16c	Tanggula Subregion	III	Eastern Asiatic Kingdom	IIIF	Qing-Tibet Plateau Subkingdom	IIIF16	Tanggute Region
44	IIIF17a	Upper and Middle of Yarlung Zangbo River Subregion	III	Eastern Asiatic Kingdom	IIIF	Qing-Tibet Plateau Subkingdom	IIIF17	Tibet-Pamir-Kunlun Region
45	IIIF17b	Qiangtang Plateau Subregion	III	Eastern Asiatic Kingdom	IIIF	Qing-Tibet Plateau Subkingdom	IIIF17	Tibet-Pamir-Kunlun Region
46	IIIF17c	Pamir-Karakoram-Kunlun Subregion	III	Eastern Asiatic Kingdom	IIIF	Qing-Tibet Plateau Subkingdom	IIIF17	Tibet-Pamir-Kunlun Region
47	IIIF18	Himalayan Region	III	Eastern Asiatic Kingdom	IIIF	Qing-Tibet Plateau Subkingdom	IIIF18	Himalayan Region
48	IVG19a	Taiwan high mountain Subregion	IV	Paleotropic Kingdom	IVG	Malaysia Subkingdom	IVG19	Taiwan Region
49	IVG19b	Taibei Subregion	IV	Paleotropic Kingdom	IVG	Malaysia Subkingdom	IVG19	Taiwan Region
50	IVG20	South Taiwan Region	IV	Paleotropic Kingdom	IVG	Malaysia Subkingdom	IVG20	South Taiwan Region
51	IVG21a	West Guangdong-North Hainan Subregion	IV	Paleotropic Kingdom	IVG	Malaysia Subkingdom	IVG21	South Sea Region
52	IVG21b	Islands of east Guangdong along sea Subregion	IV	Paleotropic Kingdom	IVG	Malaysia Subkingdom	IVG21	South Sea Region
53	IVG21c	Southwest Hainan Subregion	IV	Paleotropic Kingdom	IVG	Malaysia Subkingdom	IVG21	South Sea Region
54	IVG21d	Central Hainan Subregion	IV	Paleotropic Kingdom	IVG	Malaysia Subkingdom	IVG21	South Sea Region
55	IVG21e	Islands of South China Sea Subregion	IV	Paleotropic Kingdom	IVG	Malaysia Subkingdom	IVG21	South Sea Region
56	IVG22	Beibu Gulf Region	IV	Paleotropic Kingdom	IVG	Malaysia Subkingdom	IVG22	Beibu Gulf Region
57	IVG23	Yunnan, Myanmar and Thailand Border Region	IV	Paleotropic Kingdom	IVG	Malaysia Subkingdom	IVG23	Yunnan, Myanmar and Thailand Border Region
58	IVG24	South edge of eastern Himalayan Region	IV	Paleotropic Kingdom	IVG	Malaysia Subkingdom	IVG24	South edge of eastern Himalayan Region

**Table 2:** Composition, diversity, degree of differentiation, and endemicity of endemic seed plants for each floristic unit in China.

Floristic region code	Composition of Chinese endemic flora			Indices of differentiation			Number of local endemic species	Value of weighted endemism	Rate of Chinese endemic to native species	Rate of local endemic to native species	Rate of local to Chinese endemic species
	Num. family	Num. genus	Num. species	D_s_	D_g_	D_sf_					
IA1	27	72	127	1.76	2.67	4.70	3	18	0.10	0.00	0.02
IA2	28	64	91	1.42	2.29	3.25	17	29	0.06	0.01	0.19
IA3	43	139	268	1.93	3.23	6.23	26	72	0.11	0.01	0.10
IB4a	46	108	164	1.52	2.35	3.57	4	24	0.11	0.00	0.02
IB4b	48	146	277	1.90	3.04	5.77	4	41	0.16	0.00	0.01
IB4c	65	223	527	2.36	3.43	8.11	4	69	0.25	0.00	0.01
IIC5a	56	128	159	1.24	2.29	2.84	7	28	0.08	0.00	0.04
IIC5b	33	83	132	1.59	2.52	4.00	5	32	0.07	0.00	0.04
IIC6a	83	321	1083	3.37	3.87	13.05	16	161	0.37	0.01	0.01
IIC6b	34	70	140	2.00	2.06	4.12	0	17	0.26	0.00	0.00
IIC6c	32	86	162	1.88	2.69	5.06	16	42	0.11	0.01	0.10
IIID7	47	122	174	1.43	2.60	3.70	66	399	0.49	0.01	0.03
IIID8a	58	150	216	1.44	2.59	3.72	15	183	0.46	0.00	0.01
IIID8b	103	334	789	2.36	3.24	7.66	89	409	0.49	0.02	0.04
IIID8c	119	504	1890	3.75	4.24	15.88	81	441	0.49	0.01	0.03
IIID8d	87	278	688	2.47	3.20	7.91	13	97	0.32	0.01	0.02
IIID9a	113	446	1206	2.70	3.95	10.67	26	229	0.38	0.01	0.02
IIID9b	116	442	1285	2.91	3.81	11.08	71	296	0.33	0.02	0.05
IIID9c	122	494	1505	3.05	4.05	12.34	99	414	0.40	0.02	0.05
IIID9d	116	458	1350	2.95	3.95	11.64	56	335	0.42	0.01	0.03
IIID10a	131	609	2499	4.10	4.65	19.08	33	163	0.32	0.01	0.03
IIID10b	129	495	1463	2.96	3.84	11.34	50	262	0.34	0.01	0.03
IIID10c	138	627	2426	3.87	4.54	17.58	12	32	0.09	0.01	0.07
IIID10d	140	678	2724	4.02	4.84	19.46	7	30	0.13	0.00	0.03
IIID11a	105	314	712	2.27	2.99	6.78	13	109	0.26	0.00	0.02
IIID11b	124	499	1507	3.02	4.02	12.15	55	305	0.40	0.01	0.03
IIID11c	129	501	1422	2.84	3.88	11.02	2	70	0.33	0.00	0.00
IIID11d	139	627	2115	3.37	4.51	15.22	33	187	0.35	0.01	0.03
IIID12a	141	637	2048	3.22	4.52	14.52	16	147	0.39	0.00	0.01
IIID12b	124	433	1006	2.32	3.49	8.11	61	273	0.40	0.02	0.04
IIID12c	131	560	1531	2.73	4.27	11.69	11	162	0.39	0.00	0.01
IIIE13a	146	785	3688	4.70	5.38	25.26	167	791	0.48	0.02	0.05
IIIE13b	141	632	1807	2.86	4.48	12.82	59	365	0.33	0.01	0.03
IIIE13c	131	560	1620	2.89	4.27	12.37	60	311	0.33	0.01	0.04
IIIE14a	135	654	3240	4.95	4.84	24.00	127	716	0.45	0.02	0.04
IIIE14b	146	813	5177	6.37	5.57	35.46	429	1403	0.56	0.05	0.08
IIIE14c	106	486	2701	5.56	4.58	25.48	97	548	0.55	0.02	0.04
IIIE14d	124	574	2422	4.22	4.63	19.53	46	368	0.49	0.01	0.02
IIIE15b	91	399	1630	4.09	4.38	17.91	0	212	0.37	0.00	0.00
IIIF16a	72	274	984	3.59	3.81	13.67	156	419	0.42	0.04	0.10
IIIF16b	58	191	677	3.54	3.29	11.67	22	153	0.41	0.01	0.02
IIIF16c	55	195	734	3.76	3.55	13.35	4	102	0.42	0.00	0.01
IIIF17a	81	280	904	3.23	3.46	11.16	13	115	0.46	0.01	0.02
IIIF17b	39	103	218	2.12	2.64	5.59	93	235	0.30	0.03	0.10
IIIF17c	22	42	64	1.52	1.91	2.91	8	41	0.27	0.01	0.04
IIIF18	31	72	141	1.96	2.32	4.55	12	21	0.08	0.02	0.19
IVG19a	73	182	302	1.66	2.49	4.14	10	30	0.19	0.01	0.07
IVG19b	85	225	382	1.70	2.65	4.49	61	131	0.37	0.07	0.20
IVG20	79	147	221	1.50	1.86	2.80	70	156	0.29	0.05	0.18
IVG21a	79	158	238	1.51	2.00	3.01	51	100	0.26	0.06	0.23
IVG21c	95	299	494	1.65	3.15	5.20	3	35	0.16	0.00	0.01
IVG21d	110	380	776	2.04	3.45	7.05	19	135	0.22	0.01	0.04
IVG22	138	618	1920	3.11	4.48	13.91	65	267	0.25	0.02	0.08
IVG23	136	659	1923	2.92	4.85	14.14	177	499	0.35	0.03	0.09
IVG24	95	322	798	2.48	3.39	8.40	194	526	0.31	0.03	0.10
