# Supplementary material for: Diversity distribution patterns of Chinese endemic seed plant species and their implications for conservation planning
Source: Sci Rep. 2016 Sep 23;6:33913. doi: 10.1038/srep33913 (PMC5034272; doi:10.1038/srep33913)
Supplement: Supplementary Information [file srep33913-s1.pdf]

Manuscript title

Diversity distribution patterns of Chinese endemic seed plant species and their implications for conservation planning

Author information

Authors list

Jihong Huang, Jianhua Huang, Xinghui Lu, Keping Ma

Affiliations

Key Laboratory of Forest Ecology and Environment, the State Forestry Administration, Institute of Forest Ecology, Environment and Protection, Chinese Academy of Forestry, Beijing 100091, China; Co-Innovation Center for Sustainable Forestry in Southern China, Nanjing Forestry University, Nanjing 210037, China.

Jihong Huang & Xinghui Lu

State Key Laboratory of Vegetation and Environmental Change, Institute of Botany, Chinese Academy of Sciences, Beijing 100093, China

Jihong Huang & Keping Ma

School of Economics, Minzu University of China, Beijing 100081, China

Jianhua Huang

Contributions

Jihong, Jianhua and Keping designed the study. Jihong collected data. Jihong and Jianhua carried out the data analysis and wrote the manuscript. Jihong, Jianhua, Keping and Xinghui reviewed the manuscript.

Competing interests

The authors declare no competing financial interests.

Corresponding author

Keping Ma: Tel.: 086-10-62836223; Email: kpma@ibcas.ac.cn

Supplementary information

Additional Supplementary Information may be found in the online version of this article.

Appendix A Reference and herbarium lists that we have consulted to establish the Chinese seed plant species inventory and to collect their distribution information.

Appendix B The list and the geographic distributions of Chinese endemic seed plant species.
