# Supplementary material for: Diversity distribution patterns of Chinese endemic seed plant species and their implications for conservation planning
Source: Sci Rep. 2016 Sep 23;6:33913. doi: 10.1038/srep33913 (PMC5034272; doi:10.1038/srep33913)
Supplement: Supplementary Appendix A [file srep33913-s2.doc]

Appendix A: References and herbarium consulted to establish Chinese seed plant species inventory and collect their distribution information

References

The below list includes 1044 flora, monographs, reports or theses, and 578 articles). Here, we don’s translated Chinese into English, but retain the original language for all references for convenient to check.

1. An, M. T., H. H. Zhang, Q. Lin, T. L. Wei. 2011. Arisaema wangmoense (Araceae), a New Species from China. Novon. 21(1): 1-3.
2. Andrew, H. 2005. A new species of Calamus (Palmae) from Taiwan. Taiwania. 50(3): 222-226.
3. Auvray, G., V. Malecot. 2011. Revised lectotypification of Spartium scoparium L. (Fabaceae). Taxon. 60(5): 1480-1481.
4. Bi, H. Y., Y. T. Hou, S. X. Yu. 2010. Impatiens pingxiangensis sp nov (Balsaminaceae) from the limestone areas in Guangxi, China. Nordic Journal of Botany. 28(3): 304-308.
5. Buerki, S., P. B. Phillipson, M. W. Callmander. 2011. A Taxonomic Revision of Gouania (Rhamnaceae) in Madagascar and the Other Islands of the Western Indian Ocean (the Comoro and Mascarene Islands, and the Seychelles). Annals of the Missouri Botanical Garden. 98(2): 157-195.
6. Chang, K. C., C. C. Wang, S. L. Deng, Y. Kono, F. Y. Lu, C. I. Peng. 2011. Cotoneaster rosiflorus (Rosaceae), a new species from Taiwan. Botanical Studies. 52(2): 211-218.
7. Chaowasku, T., P. J. A. Kessler, R. W. J. M. Van der Ham. 2012. A taxonomic revision and pollen morphology of the genus Dendrokingstonia (Annonaceae). Botanical Journal of the Linnean Society. 168(1): 76-90.
8. Chen, J., K. M. Alex, L. Chan. 2007. Name changes for Chinese Pilea (Urticaceae). Novon. 17(1): 24-26.
9. Chen, L. J., Z. J. Liu. 2010. Chroniochilus sinicus, a New Species of Orchidaceae from Yunnan, China. Novon. 20(3): 252-255.
10. Chen, L., F. G. Wang, A. Q. Dong, F. W. Xing. 2011. Zingiber nanlingensis sp. nov. (Zingiberaceae) from Guangdong, China. Nordic Journal of Botany. 29(4): 431-434.
11. Chen, L., X. W. Li, J. B. Zhang, J. Q. Li. 2010. Castanopsis malipoensis and C. jinpingensis (Fagaceae), two new species from Yunnan, China. Annales Botanici Fennici. 47(4): 301-305.
12. Chen, L., Z. G. Zhang, Y. Hu, X. W. Li, J. Q. Li. 2011. A New Species and One New Name in Castanopsis (Fagaceae) from Hainan, China. Novon. 21(3): 317-321.
13. Chen, W. H., Y. M. Shui. 2010. Anoectochilus malipoensis (Orchidaceae), a new species from Yunnan, China. Annales Botanici Fennici. 47(2): 129-134.
14. Chen, X. A., J. Y. Huang, L. Consaul, X. Chen. 2010. Two New Species of Rhododendron (Ericaceae) from Guizhou, China. Novon. 20(4): 386-391.
15. Chen, X. A., L. Consaul, J. Y. Huang, H. Xie, X. Chen. 2010. Rhododendron subroseum sp. nov. and R. denudatum var. glabriovarium var. nov. (Ericaceae) from the Guizhou Province, China. Nordic Journal of Botany. 28(4): 496-498.
16. Chen, X. A., L. Consaul, J. Y. Huang, X. Chen. 2010. New taxa of Rhododendron (Ericaceae) from China. Annales Botanici Fennici. 47(5): 397-402.
17. Chen, Y. S. 2010. Cremanthodium latilobum sp. nov. and C-medogense sp. nov. (Asteraceae) from Chinese eastern Himalaya. Nordic Journal of Botany. 28(6): 756-760.
18. Chen, Y. S. 2010. Saussurea baoxingensis sp. nov. (Compositae, Cardueae) from Sichuan, China. Nordic Journal of Botany. 28(6): 761-763.
19. Chen, Y. S. 2011. A new species and a new combination in Parasenecio (Asteraceae). Annales Botanici Fennici. 48(2): 166-168.
20. Chen, Y. S. 2011. Saussurea megacephala (Asteraceae), a new species from Xizang, China. Annales Botanici Fennici. 48(2): 142-144.
21. Chen, Y. S. 2011. The identity of Parasenecio subglaber (Asteraceae) from China. Annales Botanici Fennici. 48(1): 34-36.
22. Chen, Y. S., Q. L. Gan. 2011. New species and nomenclatural action in Saussurea DC. (Asteraceae). Journal of Systematics and Evolution. 49(2): 160-161.
23. Chen, Z. L., S. J. Zeng, K. L. Wu, J. Duan. 2010. Dendrobium shixingense sp. nov. (Orchidaceae) from Guangdong, China. Nordic Journal of Botany. 28(6): 723-727.
24. Chiu, H. L., C. T. Shii, T. E. A. Yang. 2011. A New Variety of Musa itinerans (Musaceae) in Taiwan. Novon. 21(4): 405-412.
25. Choi, H. J., B. U. Oh. 2010. A new species and a new combination of Allium sect. Rhizirideum (Alliaceae) from northeastern China and Korea. Brittonia. 62(3): 199-205.
26. Choi, H. J., B. U. Oh. 2011. A partial revision of Allium (Amaryllidaceae) in Korea and north-eastern China. Botanical Journal of the Linnean Society. 167(2): 153-211.
27. Chung, K. F., H. van der Werff, C. I. Peng. 2010. Observations on the Floral Morphology of Sassafras Randaiense (Lauraceae). Annals of the Missouri Botanical Garden. 97(1): 1-10.
28. Chung, S. W., T. C. Hsu, C. I. Peng. 2010. Phacellanthus (Orobanchaceae), a newly recorded genus in Taiwan. Botanical Studies. 51(4): 531-536.
29. Cialdella, A. M., F. O. Zuloaga. 2011. Taxonomic Study of Gymnopogon (Poaceae, Chloridoideae, Cynodonteae). Annals of the Missouri Botanical Garden. 98(3): 301-330.
30. Cleal, C. J., B. A. Thomas. 2010. Botanical nomenclature and plant fossils. Taxon. 59(1): 261-268.
31. Cong, Y. Y., K. M. Liu. 2010. Impatiens obiongipetala (Balsaminaceae), a New Species from Yunnan, China. Novon. 20(4): 392-395.
32. Cong, Y. Y., Y. L. Xiang, K. M. Liu. 2010. Impatiens quadriloba sp nov (Balsaminaceae) from Sichuan, China. Nordic Journal of Botany. 28(3): 309-312.
33. De Faria, A. P. G., T. Wendt, G. K. Brown. 2010. A revision of Aechmea subgenus Macrochordion (Bromeliaceae) based on phenetic analyses of morphological variation. Botanical Journal of the Linnean Society. 162(1): 1-27.
34. de Stefano, R. D., G. C. Fernandez-Concha. 2011. Morphology-Inferred Phylogeny and a Revision of the Genus Emmotum (Icacinaceae). Annals of the Missouri Botanical Garden. 98(1): 1-27.
35. Deng, M., A. Coombes, Q. S. Li. 2011. Lectotypification of Quercus arbutifolia (Fagaceae) and the taxonomic treatment of Quercus subsect. Chrysotrichae. Nordic Journal of Botany. 29(2): 208-214.
36. Deng, M., Z. K. Zhou, A. Coombes. 2010. Lectotypification and New Synonymy in Quercus subg. Cyclobalanopsis (Fagaceae). Novon. 20(4): 400-405.
37. Deng, Y. F. 2011. The valid publication of the name Euaraliopsis (Araliaceae). Taxon. 60(5): 1482-1484.
38. Deng, Y. F., J. R. I. Wood, F. Ying. 2010. Strobilanthes biocullata (Acanthaceae), a New Species from Hunan, China. Novon. 20(4): 406-411.
39. Deng, Y. F., J. R. I. Wood, H. Li. 2010. Strobilanthes ovata (Acanthaceae), a New Species from Gaoligong Shan in Yunnan, China. Novon. 20(2): 143-146.
40. Dietrich, P., L. Xu. 2004. New species and combinations in Astragalus (Leguminosae) from China and the Himalayas. Novon. 14(2): 216-226.
41. Dong, H. J., Y. S. Chen. 2010. Ligularia qiaojiaensis sp. nov. (Asteraceae) from Yunnan, China. Nordic Journal of Botany. 28(6): 683-684.
42. Dong, S. Y. 2010. A new species of Dryopteris (Dryopteridaceae) from Hainan Island, China. Botanical Studies. 51(4): 537-542.
43. Du, X. C., Y. Ren. 2010. Hydrocotyle changanensi (Araliaceae), a new species from Shaanxi, China. Annales Botanici Fennici. 47(5): 403-407.
44. Duan, L. D., Q. Lin. 2010. Elatostema cataractum (Urticaceae), a new species from Guizhou Province, China. Annales Botanici Fennici. 47(3): 229-232.
45. Editorial committee of the flora of Taiwan. 1993. Flora of Taiwan 2nd edition, Vol. 3. Notional Taiwan University Press, Taibei.
46. Editorial committee of the flora of Taiwan. 1994. Flora of Taiwan 2nd edition, Vol. 1. Notional Taiwan University Press, Taibei.
47. Editorial committee of the flora of Taiwan. 1996. Flora of Taiwan 2nd edition, Vol. 2. Notional Taiwan University Press, Taibei.
48. Editorial committee of the flora of Taiwan. 1998. Flora of Taiwan 2nd edition, Vol. 4. Notional Taiwan University Press, Taibei.
49. Editorial committee of the flora of Taiwan. 2000. Flora of Taiwan 2nd edition, Vol. 5. Notional Taiwan University Press, Taibei.
50. Editorial committee of the flora of Taiwan. 2003. Flora of Taiwan 2nd edition, Vol. 6. Notional Taiwan University Press, Taibei.
51. Ehrendorfer, F. 2010. New and Critical Taxa of Rubia and Galium (Rubiaceae, Rubieae) for the Flora of China. Novon. 20(3): 268-277.
52. Feng, C. L., X. H. Jin. 2010. Liparis guangxiensis sp. nov. (Malaxideae: Orchidaceae) from China. Nordic Journal of Botany. 28(6): 697-698.
53. Ferrufino-Acosta, L. 2010. Taxonomic revision of the genus Smilax (Smilacaceae) in Central America and the Caribbean Islands. Willdenowia. 40(2): 227-280.
54. Gao, C. M., Y. F. Deng. 2011. Justicia addisoniensis (Acanthaceae), comb. nova. Annales Botanici Fennici. 48(1): 91-92.
55. Gao, Q., Y. Liu. 2011. Aspidistra hezhouensis (Ruscaceae s.l.), a new species from Guangxi, China. Journal of Systematics and Evolution. 49(5): 506-506.
56. Givnish, T. J. 2010. Ecology of plant speciation. Taxon. 59(5): 1326-1366.
57. Graham, S. A. 2010. Revision of the Caribbean Genus Ginoria (Lythraceae), Including Haitia from Hispaniola. Annals of the Missouri Botanical Garden. 97(1): 34-90.
58. Guo, W. Y., J. A. Yang, D. Gromyko, A. G. Ablaev, Q. Wang, C. S. Li. 2010. First record of Cercidiphylloxylon (Cercidiphyllaceae) from the Palaeocene of Fushun, NE China. Journal of Systematics and Evolution. 48(4): 302-308.
59. Guo, W., Y. Yu, Q. A. Fan, J. S. Ma, W. B. Liao. 2010. Photinia sorbifolia (Rosaceae), a new species from Hunan Province, China. Annales Botanici Fennici. 47(5): 394-396.
60. Guo, X., R. J. Wang. 2011. Hedyotis xinyiensis (Rubiaceae), a new species from China. Annales Botanici Fennici. 48(5): 443-447.
61. Guo, Y. B., N. H. Xia, R. S. Lin. 2011. Dendrocalamus exauritus, a new combination (Poaceae: Bambusoideae). Annales Botanici Fennici. 48(2): 174-176.
62. Guo, Y. B., R. S. Lin, N. H. Xia. 2010. Dendrocalamus menglongensis sp. nov. (Poaceae-Bambusoideae) from Yunnan, China. Nordic Journal of Botany. 28(4): 506-508.
63. He, H., L. B. Zhang. 2010. Polystichum kungianum, sp nov (sect. Mastigopteris, Dryopteridaceae) from Chongqing, China. Botanical Studies. 51(3): 395-401.
64. He, S. Y., P. T. Li, J. Y. Lin, G. Y. Lin, H. L. Zeng. 2011. Hoya jianfenglingensis (Apocynaceae), a New Species from Hainan, China. Novon. 21(3): 343-346.
65. He, S. Y., R. C. Zhou, P. T. Li, J. Y. Lin, W. Wu. 2011. A new species of Apocynaceae from Hainan, China. Journal of Systematics and Evolution. 49(2): 161-161.
66. He, S. Z., K. He, J. Y. Wu. 2011. Aspidistra liboensis (Ruscaceae), a new species from Guizhou, China. Annales Botanici Fennici. 48(5): 439-442.
67. He, S. Z., Q. W. Sun, Y. C. Li. 2010. Tremacron aurantiacum var. weiningense (Gesneriaceae) var. nov from Guizhou, China. Nordic Journal of Botany. 28(2): 199-201.
68. He, S. Z., W. F. Xu, Y. Y. Wang, Q. W. Sun. 2011. A New Species of Aspidistra (Ruscaceae) from Guizhou, China. Novon. 21(2): 187-189.
69. He, S. Z., Y. Y. Wang, B. L. Guo, W. F. Xu. 2010. Epimedium pudingense (Berberidaceae), a new species from Guizhou, China. Annales Botanici Fennici. 47(3): 226-228.
70. He, T., S. Chen, S. Liu. 2002. Metagentiana, a new genus of Gentianaceae. Botanical Bulletin of Academia Sinica. 43(1): 83-91.
71. Hong, D. Y. 2010. Nomenclatural Notes on the Morinaceae and Valerianaceae in China. Novon. 20(4): 418-419.
72. Hong, D. Y. 2010. Taxonomic Notes on Chinese Campanulaceae. Novon. 20(4): 420-425.
73. Hong, D. Y. 2011. Paeonia rotundiloba (D. Y. Hong) D. Y. Hong: A new status in tree peonies (Paeoniaceae). Journal of Systematics and Evolution. 49(5): 464-467.
74. Hong, D. Y., S. Ge. 2010. Taxonomic Notes on the Genus Adenophora (Campanulaceae) in China. Novon. 20(4): 426-428.
75. Hou, Y. T., X. R. Zhou, S. X. Yu. 2011. Impatiens parvisepala (Balsaminaceae), a new species from Guangxi, China. Annales Botanici Fennici. 48(1): 57-62.
76. Hsu, T. C., C. M. Kuo. 2011. Gastrodia albida (Orchidaceae), a new species from Taiwan. Annales Botanici Fennici. 48(3): 272-275.
77. Hsu, T. W., Y. Kono, T. Y. Chiang, C. I. Peng. 2011. Ypsilandra (Melanthiaceae: Liliaceae sensu lato), a new generic record for Taiwan. Botanical Studies. 52(1): 99-104.
78. Hu, X. M., Q. W. Zeng, L. Fu, F. W. Xing. 2011. Manglietia Kaifui (Magnoliaceae), a New Species from Yunnan, China. Pakistan Journal of Botany. 43(5): 2269-2275.
79. Hu, X., N. A. Liu. 2010. Hedychium longipetalum (Zingiberaceae), a new species from Yunnan, China. Annales Botanici Fennici. 47(3): 237-239.
80. Hu, X., N. Liu. 2010. Hedychium menghaiense (Zingiberaceae), a new species from Yunnan, China. Journal of Systematics and Evolution. 48(2): 146-151.
81. Huang, S. X., Y. G. Wei, W. H. Luo. 2010. Chirita nandanensis (Gesneriaceae), a new species from Guangxi, China. Annales Botanici Fennici. 47(2): 139-140.
82. Huang, Y. F., R. H. Jiang, D. X. Nong, W. B. Xu. 2011. Chionographis shiwandashanensis sp nov (Melanthiaceae) from southern Guangxi, China. Nordic Journal of Botany. 29(5): 605-607.
83. Huang, Y. S., W. H. Wu, W. B. Xu, Y. Liu. 2011. Firmiana calcarea sp nov (Malvaceae) from limestone areas in Guangxi, China. Nordic Journal of Botany. 29(5): 608-610.
84. Iokawa, H. Ohashi. 2002. A taxonomic study of the genus Campylotropis (Leguminosae) I. The Journal of Japanese Botany. 77(4): 179-195.
85. Iokawa, H. Ohashi. 2002. A taxonomic study of the genus Campylotropis (Leguminosae) II. The Journal of Japanese Botany. 77(5): 251-252.
86. Iokawa, H. Ohashi. 2002. A taxonomic study of the genus Campylotropis (Leguminosae) III. The Journal of Japanese Botany. 77(6): 315-319.
87. Jaanus, P., T. Tiiu, V. Malle. 2004. Vegetation of Estonian watercourses: the drainage basin of the southern coast of the Gulf of Finland. Annals Botanici Fennici. 41(3): 157-177.
88. JI, Y., H. Li, Z. Xu. 2004. Arisaema menglaense (Araceae), a new species from southern Yunnan, China. Annals Botanici Fennici. 41(2): 133-135.
89. Jiang, N., X. Lin, K. Y. Guan, W. B. Yu. 2011. Valid publication of the name Helicia falcata (Proteaceae). Nordic Journal of Botany. 29(1): 61-62.
90. Jiang, N., X. M. Peng, W. B. Yu. 2011. Valid Publication of Asarum longirhizomatosum (Aristolochiaceae). Novon. 21(2): 190-191.
91. Jiang, R. H., X. C. Zhang, Y. Liu. 2011. Asplenium cornutissimum (Aspleniaceae), a new species from karst caves in Guangxi, China. Brittonia. 63(1): 83-86.
92. Jiang, W., D. Z. Li, H. Wang. 2010. Thladiantha tomentosa (Cucurbitaceae) comb. nov. from southwestern China. Nordic Journal of Botany. 28(6): 699-701.
93. Jiang, Y. S., Y. Zhang, Y. Wang, F. Wen. 2011. Petrocodon multiflorus sp nov (Gesneriaceae) from Guangxi, China. Nordic Journal of Botany. 29(1): 57-60.
94. Jiang, Y., X. Zhu, Y. Du, H. Ohashi. 2004. A new species of Crotalaria L. (Leguminosae) from Zhejiang Province, China. The Journal of Japanese Botany. 79(6): 373-375.
95. Jin, M., S. Wu. 2003. Two new species of Arisaema (Araceae) from China. The Journal of Japanese Botany. 78(2): 81-85.
96. Jin, X. 2005. Bulbophyllum wuzhishanensis (Orchidaceae), a new species from Hainan, China. Brittonia. 57(3): 255-257.
97. Jin, X. 2005. Coelogyne weixiensis (Orchidaceae), a new species from Yunnan, China. Annals Botanici Fennici. 42(2): 135-137.
98. Jin, X. F., B. Y. Ding, Y. J. Zhang, D. Y. Hong. 2010. A Taxonomic Revision of Rhododendron Subg. Tsutsusi Sect. Brachycalyx (Ericaceae). Annals of the Missouri Botanical Garden. 97(2): 163-190.
99. Jin, X. F., C. Z. Zheng. 2010. Carex yandangshanica sp. nov. (Cyperaceae: C. sect. Rhomboidales) from Zhejiang, China. Nordic Journal of Botany. 28(6): 709-712.
100. Jin, X. F., Y. J. Zhao, C. Z. Zheng, H. W. Zhang. 2011. Carex zhejiangensis sp nov (Cyperaceae) from Zhejiang, eastern China. Nordic Journal of Botany. 29(1): 63-70.
101. Jin, X. H. 2010. Liparis cordifolia var. gongshanensis (Orchidaceae, Malaxideae), a New Variety from Yunnan, China. Novon. 20(3): 282-284.
102. Jin, X. H. 2011. Liparis cheniana (Malaxideae: Orchidaceae), a new species from Xizang, China. Annales Botanici Fennici. 48(2): 163-165.
103. Jin, X. H., D. Z. Li, X. G. Xiang, Y. J. Lai, X. C. Shi. 2012. Nujiangia (Orchidaceae: Orchideae): A new genus from the Himalayas. Journal of Systematics and Evolution. 50(1): 64-71.
104. Jin, X. H., Z. Q. Dai, Q. Y. Liu, X. Y. Ju, X. G. Xiang. 2011. Cephalanthera humilis sp nov (Orchidaceae) from Yunnan, China. Nordic Journal of Botany. 29(5): 598-600.
105. Jin, X., S. Chen H. Qin, G. Zhu, S. L. Gloria. 2004. A new species of Holcoglossum (Orchidaceae) from China. Novon. 14(2): 178-179.
106. Jin, X., S. Chen, H. Qin, G. Zhu, S. L. Gloria. 2004. A new species of Didymoplexiella (Orchidaceae) from China. Novon. 14(2): 176-177.
107. Lammers, T. G. 2011. Revision of the Infrageneric Classification of Lobelia L. (Campanulaceae: Lobelioideae). Annals of the Missouri Botanical Garden. 98(1): 37-62.
108. Li, G. F., Y. M. Shui, W. H. Chen, Z. D. Wei. 2011. A new species of Impatiens (Balsaminaceae) from Yunnan, China. Brittonia. 63(4): 452-456.
109. Li, J. M., S. X. Zhu. 2010. Chirita auriculata (Gesneriaceae), a new species from China, placed in sect. Chirita based on molecular data. Annales Botanici Fennici. 47(3): 221-225.
110. Li, R., S. D. Zhang. 2011. Aletris simpliciflora sp. nov. (Nartheciaceae) from southwest Xizang, China. Nordic Journal of Botany. 29(4): 440-442.
111. Li, T., H. Zhang. 2010. A New Species of Rhodiola (Crassulaceae) from Western Sichuan, China. Novon. 20(1): 53-56.
112. Li, X. W., J. Q. Li. 2010. A review of the infrageneric taxonomy and nomenclature of Actinidia (Actinidiaceae). Annales Botanici Fennici. 47(2): 106-108.
113. Li, Y. L., Z. J. Gong, C. C. Wang, J. Shen. 2010. New species and new records of diatoms from Lake Fuxian, China. Journal of Systematics and Evolution. 48(1): 65-72.
114. Liang, S. Y., S. R. Zhang. 2010. New Combinations in Schoenoplectus (Cyperaceae) from China. Novon. 20(2): 170-171.
115. Liang, Y. S., C. H. Chen, J. L. Tsai. 2011. Lindernia kinmenensis sp. nov. (Scrophulariaceae) from Kinmen (Taiwan). Nordic Journal of Botany. 29(4): 435-439.
116. Lin, C. R., C. I. Peng, Y. Kono, Y. Liu. 2010. Aspidistra obconica, Asparagaceae [Ruscaceae], a new species from limestone areas in Guangxi, China. Botanical Studies. 51(2): 263-268.
117. Lin, C. R., W. B. Xu, Y. Liu. 2011. Aspidistra albiflora sp. nov. (Asparagaceae) from southwestern Guangxi, China. Nordic Journal of Botany. 29(4): 443-446.
118. Lin, C. R., Y. Liu. 2011. Aspidistra longituba (Ruscaceae), a new species from Guangxi, China. Annales Botanici Fennici. 48(6): 519-521.
119. Lin, C. R., Y. Liu. 2011. Aspidistra punctatoides sp nov (Ruscaceae) from limestone areas in Guangxi, China. Nordic Journal of Botany. 29(2): 189-193.
120. Lin, J. Y., S. Y. He, M. Li, G. Y. Lin. 2010. Heterostemma pingtaoi (Apocynaceae, Asclepiadoideae), a New Species from Hainan, China. Novon. 20(1): 60-62.
121. Lin, Q. W., Z. X. Zhang. 2010. Microtropis longicarpa and M. daweishanensis (Celastraceae), two new species from Yunnan, China. Annales Botanici Fennici. 47(2): 141-147.
122. Lin, Q., L. D. Duan, Z. R. Yang, Y. M. Shui. 2011. Notes on Elatostema section Androsyce Wedd. (Urticaceae). Journal of Systematics and Evolution. 49(2): 163-163.
123. Lin, Q., Y. M. Shui, L. D. Duan. 2011. Elatostema oppositum (Urticaceae), a New Species from Yunnan, China. Novon. 21(2): 212-215.
124. Lin, Q., Z. R. Yang, L. D. Duan, T. G. Gao. 2011. Miscellaneous taxonomic notes on Elatostema (Urticaceae) from China and its adjacent area. Nordic Journal of Botany. 29(5): 590-597.
125. Lin, Q., Z. R. Yang. 2011. Validation of the Name Asparagus kansuensis (Asparagaceae). Novon. 21(1): 69-70.
126. Lin, Q., Z. R. Yang. 2011. Validation of Two Names, Berchemia omeiensis and Rhamnus kwangsiensis (Rhamnaceae). Novon. 21(1): 71-72.
127. Linder, H. P., M. Baeza, N. P. Barker, C. Galley, A. M. Humphreys, K. M. Lloyd, D. A. Orlouch, M. D. Purie, B. K. Simon, N. Walsh, G. A. Verboom. 2010. A Generic Classification of the Danthonioideae (Poaceae). Annals of the Missouri Botanical Garden. 97(3): 306-364.
128. Liu, B., H.-N. Qin. 2013. Taxonomic revision of the Symplocos nakaharae complex (Symplocaceae) with special reference to fruit morphology. Journal of Systematics and Evolution. 51(1): 94-114.
129. Liu, X. L., X. N. Yue, Z. Y. Chang, L. R. Xu. 2011. A New Species of Astragalus (Leguminosae) from Northwestern Xinjiang, China. Novon. 21(2): 216-218.
130. Liu, Y. C., H. Peng. 2010. Miscanthus subgen. Rubimons subgen. nov (Poaceae) and Miscanthus villosus sp. nov. from southwest Yunnan, China. Nordic Journal of Botany. 28(6): 670-672.
131. Liu, Y. C., H. Peng. 2011. Microstegium butuoense (Poaceae), a new species from Sichuan, China. Annales Botanici Fennici. 48(2): 182-184.
132. Liu, Y., D. G. Zhang, Q. E. Yang. 2010. Sinosenecio hupingshanensis (Asteraceae), a new species from Hunan and Hubei, China. Botanical Studies. 51(3): 387-394.
133. Liu, Y., D. G. Zhang, Q. E. Yang. 2011. Sinosenecio albonervius (Asteraceae), a new species from Hunan and Hubei, China. Botanical Studies. 52(3): 359-365.
134. Liu, Y., Q. E. Yang. 2010. Sinosenecio yilingii (Asteraceae), a new species from Sichuan, China. Botanical Studies. 51(2): 269-275.
135. Liu, Y., Q. E. Yang. 2011. Hainanecio, a new genus of the Senecioneae, Asteraceae from China. Botanical Studies. 52(1): 115-120.
136. Liu, Y., Q. E. Yang. 2011. Sinosenecio sichuanicus (Asteraceae), a new species from Sichuan, China. Botanical Studies. 52(2): 219-223.
137. Liu, Y., W. B. Xu, B. Pan. 2010. Wentsaiboea tiandengensis sp. nov. and W. luochengensis sp. nov. (Gesneriaceae) from Karst caves in Guangxi, southern China. Nordic Journal of Botany. 28(6): 739-745.
138. Liu, Y., W. B. Xu, Y. S. Huang. 2011. Primulina guangxiensis sp nov (Gesneriaceae) from a karst cave in Guangxi, China. Nordic Journal of Botany. 29(6): 682-686.
139. Liu, Y., Y. Kono, C. R. Lin, W. B. Xu, C. I. Peng. 2011. Aspidistra erecta (Asparagaceae), a new species from limestone areas in Guangxi, China. Botanical Studies. 52(3): 367-373.
140. Liu, Z. Y., Y. Liu, Q. E. Yang. 2011. Sinosenecio nanchuanicus (Asteraceae), a new species small in size yet high in chromosome number from Chongqing, China. Botanical Studies. 52(1): 105-113.
141. Long, C., Z. Dao, H. Li. 2003. A new species of Cymbidium (Orchidaceae) from Tibet (Xizang), China. Novon. 13(2): 203-205.
142. Ma, H., N. Jiang, W. B. Yu, Z. H. Li. 2011. Valid publication of the name Callicarpa peichieniana (Lamiaceae). Nordic Journal of Botany. 29(2): 224-226.
143. Ma, H., Q. J. Pan, L. Wang, Z. H. Li, Y. M. Wan, X. X. Liu. 2011. Musella lasiocarpa var. rubribracteata (Musaceae), a New Variety from Sichuan, China. Novon. 21(3): 349-353.
144. Martin-Bravo, S., V. Valcarcel, P. Vargas, M. Luceno. 2010. Geographical speciation related to Pleistocene range shifts in the western Mediterranean mountains (Reseda sect. Glaucoreseda, Resedaceae). Taxon. 59(2): 466-482.
145. Meng, S. Y., J. L. Wang, Q. R. Liu. 2011. On the identity of Euonymus pallidifolia (Celastraceae). Annales Botanici Fennici. 48(2): 185-187.
146. Mou, F. J., D. X. Zhang. 2010. Rubovietnamia nonggangensis (Rubiaceae), a new species from China. Botanical Studies. 51(1): 119-126.
147. Nong, D. X., W. B. Xu, W. H. Wu, Y. Liu. 2010. Lysionotus fengshanensis Yan Liu & D. X. Nong sp. nov. (Gesneriaceae) from Guangxi, China. Nordic Journal of Botany. 28(6): 720-722.
148. Nong, H. T., L. S. Wu. 2010. Two New Species of Gentianaceae from Northwestern Yunnan, China. Novon. 20(2): 166-169.
149. Ohashi, H. 2005. A new species of Pueraria (Leguminosae) from Guizhou, China. The Journal of Japanese Botany. 80(1): 9-13.
150. Ohashi, H., R. R. Mill. 2000. Hylodesmum, a new name for Podocarpium (Leguminosae). Edinburgh Journal of Botany. 57(2): 171.
151. Pan, B., X. Y. Zhu. 2010. Taxonomic revision of Dumasia (Fabaceae, Papilionoideae). Annales Botanici Fennici. 47(4): 241-256.
152. Paszko, B., H. Y. Ma. 2011. Taxonomic revision of the Calamagrostis epigeios complex with particular reference to China. Journal of Systematics and Evolution. 49(5): 495-504.
153. Paul, O. 2002. Some brief notes on Taiwanese orchids 1. Taiwania. 47(4): 239-245.
154. Paul, O. 2003. Orchidaceous additions to the floras of China and Taiwan. Taiwania. 48(2): 87-91.
155. Paul, O. 2003. Orchidaceous additions to the floras of China and Vietnam. Taiwania. 48(3): 139-146.
156. Paul, O. 2004. Orchidaceous additions to the floras of China and Taiwan (II). Taiwania. 49(2): 95-101.
157. Peng, C. I., Y. Liu, S. M. Ku, Y. Kono, K. F. Chung. 2010. Begonia xbreviscapa (Begoniaceae), a new intersectional natural hybrid from limestone areas in Guangxi, China. Botanical Studies. 51(1): 107-117.
158. Peng, C., Y. Chen, W. Leong. 2005. Five new species of Begonia (Begoniaceae) from Taiwan. Botanical Bulletin of Academia Sinica. 46(3): 255-272.
159. Peng, C., Y. Liu, S. Ku. 2005. Begonia fangii (sect. Coelocentrum, Begoniaceae), a new species from limestone areas in Guangxi, China. Botanical Bulletin of Academia Sinica. 46(1): 83-90.
160. Peng, X. M., N. Jiang, W. B. Yu. 2011. Validation of the name Callicarpa bodinieri var. iteophylla (Lamiaceae). Journal of Systematics and Evolution. 49(5): 508-508.
161. Qaiser, M., T. Siddiqui, S. S. Shaukat. 2011. Two New Species of Euphrasia (Orobanchanceae) from Pakistan and Adjoining Areas. Pakistan Journal of Botany. 43(4): 1809-1818.
162. Qi, Y., Y. Wang. 2004. Wikstroemia fuminensis (Thymelaeaceae), a new species from Yunnan, China. Novon. 14(3): 324-326.
163. Ren, C., Q. E. Yang. 2010. The identity of Ligularia yui (Asteraceae: Senecioneae) from China. Nordic Journal of Botany. 28(6): 716-719.
164. Ren, Z. X., H. Wang, X. H. Jin, D. Z. Li. 2011. Calanthe yaoshanensis sp nov (Orchidaceae) from northeastern Yunnan, China. Nordic Journal of Botany. 29(1): 54-56.
165. Rong, Y. S., H. Ya, X. Bo, S. M. Liang, L. Juan. 2010. Gastrochilus brevifimbriatus (Orchidaceae, Aeridinae), a New Species from Chongqing, China. Novon. 20(1): 113-116.
166. Rouhan, G., R. C. Moran. 2011. Revision of Paleotropical Megalastrum (Dryopteridaceae). Annals of the Missouri Botanical Garden. 98(1): 90-100.
167. Sabirhazi, G., B. R. Pan, G. M. Shen, M. L. Zhang. 2010. Calligonum taklimakanense sp. nov. (Polygonaceae) from Xinjiang, China. Nordic Journal of Botany. 28(6): 680-682.
168. Shao, J. R., M. L. Zhou, X. M. Zhu, D. Z. Wang, D. Q. Bai. 2011. Fagopyrum wenchuanense and Fagopyrum qiangcai, Two New Species of Polygonaceae from Sichuan, China. Novon. 21(2): 256-261.
169. Shen, R. J., S. S. Lin, Y. Yu, D. F. Cui, W. B. Liao. 2010. Chiritopsis danxiaensis sp. nov. (Gesneriaceae) from Mount Danxiashan, south China. Nordic Journal of Botany. 28(6): 728-732.
170. Sukhorukov, A. P. 2011. Axyris (Chenopodiaceae s.str. or Amaranthaceae s.l.) in the Himalayas and Tibet. Willdenowia. 41(1): 75-82.
171. Sun, N., X. J. He, S. D. Zhou. 2010. Epidermal morphology of Ligusticum (Apiaceae) from China. Annales Botanici Fennici. 47(4): 261-279.
172. Sun, Q. A., Q. Lin, Z. L. Yi, Z. R. Yang, F. S. Zhou. 2010. A taxonomic revision of Miscanthus s.l. (Poaceae) from China. Botanical Journal of the Linnean Society. 164(2): 178-220.
173. Tang, H., F. Wen. 2011. Chirita tiandengensis (Gesneriaceae) sp nov from Guangxi, China. Nordic Journal of Botany. 29(2): 233-237.
174. Tang, Y., M. L. Zhou, D. Q. Bai, J. R. Shao, X. M. Zhu, D. Z. Wang, Y. X. Tang. 2010. Fagopyrum pugense (Polygonaceae), a New Species from Sichuan, China. Novon. 20(2): 239-242.
175. Tang, Y., Z. L. Dao, H. Li. 2011. Elaeocarpus gaoligongshanensis and E. dianxiensis (Elaeocarpaceae), two new species from Yunnan, China. Annales Botanici Fennici. 48(2): 169-173.
176. The Biodiversity Committee of Chinese Academy of Sciences. 2012. Catalogue of Life China: 2012 Annual Checklist. Science Press, Beijing.
177. Tian, B., T. L. Liu, J. Q. Liu. 2010. Ostryopsis intermedia, a new species of Betulaceae from Yunnan, China. Botanical Studies. 51(2): 257-262.
178. Toshio, Y., B. David. 2010. New Species of Meconopsis (Papaveraceae) from Mianning,Southwestern Sichuan,China. 云南植物研究. 32(6): 503-507.
179. Toshio, Y., B. David. 2011. New Species of Meconopsis (Papaveraceae) from Balang Shan, Western Sichuan, China. 植物分类与资源学报. 33(4): 409-413.
180. Tseng, Y. H., C. C. Wang. 2011. Alpinia oui (Zingiberaceae), a New Species from Taiwan. Novon. 21(2): 270-273.
181. Tseng, Y. H., C. T. Chao, H. W. Lin. 2011. A new species of Tylophora from coral reef areas in Hengchun Peninsula, Taiwan, China. Journal of Systematics and Evolution. 49(2): 162-162.
182. Tseng, Y. H., C. T. Chao. 2011. Tylophora lui (Apocynaceae), a new species from Taiwan. Annales Botanici Fennici. 48(6): 515-518.
183. Ulloa, C. U., D. L. Nickrent, C. Whitefoord, D. L. Kelly. 2010. Hondurodendron, a New Monotypic Genus of Aptandraceae from Honduras. Annals of the Missouri Botanical Garden. 97(3): 457-467.
184. van Welzen, P. C. 2010. Revision of the Asian Genus Koilodepas (Euphorbiaceae). Annals of the Missouri Botanical Garden. 97(2): 218-234.
185. Vanijajiva, O., J. W. Kadereit. 2011. A revision of Gynura (Asteraceae: Senecioneae). Journal of Systematics and Evolution. 49(4): 285-314.
186. von Raab-Straube, E. 2011. The genus Saussurea (Compositae, Cardueae) in China: taxonomic and nomenclatural notes. Willdenowia. 41(1): 83-95.
187. Vu, X.-P., T.-X. Do, 温放, 韦毅刚. 2011. 越南蛛毛苣苔属(苦苣苔科)植物新记录(英文). 广西植物. 31(3): 288-290.
188. Wang, C. B., X. G. Ma, X. J. He. 2011. Bupleurum candollei var. paucefulcrans comb. nov. (Apiaceae) from Guizhou, China: comparison of allied species based on morphology, anatomy and molecular data. Nordic Journal of Botany. 29(4): 424-430.
189. Wang, F. G., D. M. Liu, F. W. Xing. 2010. Two new species of Hypodematium (Hypodematiaceae) from limestone areas in Guangdong, China. Botanical Studies. 51(1): 99-106.
190. Wang, F. G., H. F. Chen, F. W. Xing. 2011. Davallia napoensis, a New Species of Davalliaceae from Guangxi, China. Novon. 21(3): 380-384.
191. Wang, J., Y. S. Ye, H. G. Ye. 2010. Ainsliaea asaroides sp nov (Asteraceae) from Guangdong, China. Nordic Journal of Botany. 28(2): 196-198.
192. Wang, Q. A., D. Y. Hong. 2011. Character analysis and taxonomic revision of the Microtoena insuavis complex (Lamiaceae). Botanical Journal of the Linnean Society. 165(3): 315-327.
193. Wang, Q., C. Yand, X. Zhu, H. Ohashi. 2001. A new species of Oxytropis DC. (Leguminosae) from Xinjiang, China. The Journal of Japanese Botany. 76(4): 219-221.
194. Wang, R. 2002. Two new species of Spiradiclis (Rubiaceae) from China. Novon. 12(3): 420-423.
195. Wang, X. 2010. Schmeissneria: An angiosperm from the Early Jurassic. Journal of Systematics and Evolution. 48(5): 326-335.
196. Wang, Y.-Z., R.-B. Mao, Y. Liu, J.-M. Li, Y. Dong, Z.-Y. Li, J. F. Smith. 2011. Phylogenetic reconstruction of Chirita and allies (Gesneriaceae) with taxonomic treatments. Journal of Systematics and Evolution. 49(1): 50-64.
197. Wang, Z., H. Li, F. Bian. 2002. Typhonium jinpingense, a new species from Yunnan, China, with the lowest diploid chromosome number in Araceae. Novon. 12(2): 286-289.
198. Weber, A., D. J. Middleton, A. Forrest, R. Kiew, C. L. Lim, A. R. Rafidah, S. Sontag, P. Triboun, Y.-G. Wei, T. L. Yao, M. Möller. 2011. Molecular systematics and remodelling of Chirita and associated genera (Gesneriaceae) Taxon. 60(3): 767-790.
199. Wei, C., Y. Ping. 2010. Astragalus beitashanensis, a New Species of Leguminosae from Xinjiang, China. Novon. 20(1): 21-22.
200. Wei, Y. G., W. T. Wang. 2010. Pellionia ronganensis sp nov (Urticaceae) from Guangxi, China. Nordic Journal of Botany. 28(1): 54-55.
201. Wei, Y. G., W. T. Wang. 2011. Elatostema recurviramum (Urticaceae), a New Cave-dwelling Species from Guangxi, China. Novon. 21(2): 281-284.
202. Wei, Y. G., W. T. Wang. 2011. Elatostema xanthotrichum and E. bamaense (Urticaceae), two new species from Guangxi, China. Annales Botanici Fennici. 48(1): 93-95.
203. Wu, L. S., H. T. Nong. 2010. Two New Species of Saussurea (Asteraceae) from the Qinghai-Xizang Plateau, China. Novon. 20(2): 172-174.
204. Wu, W. H., W. B. Xu, D. X. Nong, Y. Liu. 2011. Chirita ningmingensis (Gesneriaceae), a new species from Guangxi, China. Annales Botanici Fennici. 48(5): 422-424.
205. Wu, Z. Y., W. T. Wang, H. Wang, D. Z. Li. 2011. Elatostema densistriolatum sp nov., E-latistipulum sp nov and E-cyrtandrifolium var. hirsutum var. nov (Urticaceae) from southwest China. Nordic Journal of Botany. 29(2): 227-232.
206. Wu, Z., P. H. Raven, D. Hong. 1994. Flora of China, Vol. 17. Science Press or Missouri Botanical Garden Press, Beijing or St. Louis.
207. Wu, Z., P. H. Raven, D. Hong. 1995. Flora of China, Vol. 16. Science Press or Missouri Botanical Garden Press, Beijing or St. Louis.
208. Wu, Z., P. H. Raven, D. Hong. 1995. Flora of China, Vol. 6. Science Press or Missouri Botanical Garden Press, Beijing or St. Louis.
209. Wu, Z., P. H. Raven, D. Hong. 1996. Flora of China, Vol. 15. Science Press or Missouri Botanical Garden Press, Beijing or St. Louis.
210. Wu, Z., P. H. Raven, D. Hong. 1998. Flora of China, Vol. 18. Science Press or Missouri Botanical Garden Press, Beijing or St. Louis.
211. Wu, Z., P. H. Raven, D. Hong. 1999. Flora of China, Vol. 4. Science Press or Missouri Botanical Garden Press, Beijing or St. Louis.
212. Wu, Z., P. H. Raven, D. Hong. 2000. Flora of China, Vol. 24. Science Press or Missouri Botanical Garden Press, Beijing or St. Louis.
213. Wu, Z., P. H. Raven, D. Hong. 2001. Flora of China, Vol. 8. Science Press or Missouri Botanical Garden Press, Beijing or St. Louis.
214. Wu, Z., P. H. Raven, D. Hong. 2003. Flora of China, Vol. 5. Science Press or Missouri Botanical Garden Press, Beijing or St. Louis.
215. Wu, Z., P. H. Raven, D. Hong. 2003. Flora of China, Vol. 9. Science Press or Missouri Botanical Garden Press, Beijing or St. Louis.
216. Wu, Z., P. H. Raven, D. Hong. 2005. Flora of China, Vol. 14. Science Press or Missouri Botanical Garden Press, Beijing or St. Louis.
217. Wu, Z., P. H. Raven, D. Hong. 2006. Flora of China, Vol. 22. Science Press or Missouri Botanical Garden Press, Beijing or St. Louis.
218. Wu, Z., P. H. Raven, D. Hong. 2007. Flora of China, Vol. 12. Science Press or Missouri Botanical Garden Press, Beijing or St. Louis.
219. Wu, Z., P. H. Raven, D. Hong. 2007. Flora of China, Vol. 13. Science Press or Missouri Botanical Garden Press, Beijing or St. Louis.
220. Wu, Z., P. H. Raven, D. Hong. 2008. Flora of China, Vol. 11. Science Press or Missouri Botanical Garden Press, Beijing or St. Louis.
221. Wu, Z., P. H. Raven, D. Hong. 2008. Flora of China, Vol. 7. Science Press or Missouri Botanical Garden Press, Beijing or St. Louis.
222. Wu, Z., P. H. Raven, D. Hong. 2009. Flora of China, Vol. 25. Science Press or Missouri Botanical Garden Press, Beijing or St. Louis.
223. Wu, Z., P. H. Raven, D. Hong. 2010. Flora of China, Vol. 10. Science Press or Missouri Botanical Garden Press, Beijing or St. Louis.
224. Wu, Z., P. H. Raven, D. Hong. 2010. Flora of China, Vol. 23. Science Press or Missouri Botanical Garden Press, Beijing or St. Louis.
225. Wu, Z., P. H. Raven, D. Hong. 2011. Flora of China, Vol. 19. Science Press or Missouri Botanical Garden Press, Beijing or St. Louis.
226. Wu, Z., P. H. Raven, D. Hong. 2011. Flora of China, Vol. 20. Science Press or Missouri Botanical Garden Press, Beijing or St. Louis.
227. Wu, Z., P. H. Raven, D. Hong. 2011. Flora of China, Vol. 21. Science Press or Missouri Botanical Garden Press, Beijing or St. Louis.
228. Xia, G. H., C. H. Liu, W. Y. Xie, G. Y. Li. 2011. Pseudostellaria tianmushanensis sp nov (Caryophyllaceae) from Zhejiang, China. Nordic Journal of Botany. 29(2): 204-207.
229. Xiang, C. L., E. D. Liu, H. Peng. 2010. Nomenclatural notes on the genus Paraphlomis (Lamiaceae: Lamioideae) from China. Nordic Journal of Botany. 28(6): 667-669.
230. Xiang, C. L., E. D. Liu. 2010. Validation of the name Vitis yunnanensis (Vitaceae) endemic to Yunnan, China. Nordic Journal of Botany. 28(6): 764-765.
231. Xu, H., Y. D. Li, H. J. Yang, H. Q. Chen. 2011. Two New Species of Aristolochia (Aristolochiaceae) from Hainan Island, China. Novon. 21(2): 285-289.
232. Xu, H., Y. D. Li, H. Q. Chen. 2011. A New Species of Sciaphila (Triuridaceae) from Hainan Island, China. Novon. 21(1): 154-157.
233. Xu, W. B., B. Pan, Y. Liu. 2011. Petrocosmea huanjiangensis, a New Species of Gesneriaceae from Limestone Areas in Guangxi, China. Novon. 21(3): 385-387.
234. Xu, W. B., B. Pan, Y. S. Huang, Y. Liu. 2010. Chirita leprosa sp. nov. (Gesneriaceae) from limestone areas in Guangxi, China. Nordic Journal of Botany. 28(6): 705-708.
235. Xu, W. B., B. Pan, Y. S. Huang, Y. Liu. 2011. Chirita lijiangensis (Gesneriaceae), a new species from limestone area in Guangxi, China. Annales Botanici Fennici. 48(2): 188-190.
236. Xu, W. B., W. H. Wu, D. X. Nong, Y. Liu. 2010. Hemiboea purpurea sp nov (Gesneriaceae) from a limestone area in Guangxi, China. Nordic Journal of Botany. 28(3): 313-315.
237. Xu, W. B., Y. S. Huang, L. Wu, Y. Liu. 2011. Chirita luochengensis (Gesneriaceae), a new species from limestone areas in northern Guangxi, China. Brittonia. 63(2): 314-317.
238. Xu, W. F., S. Z. He, L. Yang. 2010. Aspidistra chishuiensis (Ruscaceae), a new species from Guizhou, China. Annales Botanici Fennici. 47(2): 118-120.
239. Yan, H. J., Z. J. Fang, H. Y. Zhang, S. Yu. 2011. Fallopia multiflora var. angulata, a New Combination in the Polygonaceae from China. Novon. 21(3): 388-391.
240. Yang, H. Q., M. S. Sun, Y. M. Yang. 2011. Fargesia huizensis (Poaceae: Bambusoideae), a new species from Yunnan, China. Annales Botanici Fennici. 48(5): 425-427.
241. Yang, Y. 2005. A new species of Ephedra L. (Ephedraceae) from Sichuan, China with a note on its systematic significance. Botanical Bulletin of Academia Sinica. 46(4): 363-366.
242. Yang, Y., X. W. Zhang, J. H. Wiersema. 2011. Notes on the typification of Beilschmiedia xizangensis (Lauraceae). Taxon. 60(2): 577-578.
243. Yang, Z. R., L. D. Duan, Q. Lin. 2011. Elatostema scaposum sp. nov. (Urticaceae) from Guizhou, China. Nordic Journal of Botany. 29(4): 420-423.
244. Yeh, C. L., C. S. Leou, T. C. Hsu, C. R. Yeh. 2011. Gastrodia sui sp. nov. (Orchidaceae) from Taiwan. Nordic Journal of Botany. 29(4): 417-419.
245. Yin, J. T., G. Gusman. 2010. Arisaema linearifolium (Araceae), a new species from northern Yunnan, China. Annales Botanici Fennici. 47(1): 76-78.
246. Yin, J., H. Li, Z. Xu. 2004. Arisaema menghaiense (Araceae), a new epiphytic species from South Yunnan, China. Novon. 14(3): 372-374.
247. Yu, H., S. Zhang. 2005. Epigeneium gaoligongense (Orchidaceae), a new species from Yunnan, China. Novon. 15(3): 495-497.
248. Yu, N. N., Y. Jia, J. C. Zhao. 2011. Synonymy and Typifications in Groutiella tomentosa (Orthotrichaceae, Bryopsida). Novon. 21(2): 290-293.
249. Yu, W. B., P. H. Huang, D. Z. Li, H. Wang. 2010. A New Species of Pedicularis (Orobanchaceae) from the Hengduan Mountains, Southwestern China. Novon. 20(4): 512-518.
250. Yu, W. B., P. H. Huang, H. Wang. 2011. A new synonym of Pedicularis cyathophylla (Orobanchaceae). Journal of Systematics and Evolution. 49(5): 507-507.
251. Yu, W. T., S. T. Chen, Z. K. Zhou. 2011. Microula pentagona sp nov and M-galactantha sp nov (Boraginaceae) from the eastern Qinghai-Tibetan Plateau. Nordic Journal of Botany. 29(2): 215-220.
252. Yuan, Y. M., Y. Song, X. J. Ge. 2011. Impatiens qingchengshanica (Balsaminaceae), a unique new species from China and its phylogenetic position. Botanical Studies. 52(2): 225-230.
253. Zhang, J. W., D. E. Boufford, H. Sun. 2011. Parasyncalathium JW Zhang, Boufford & H. Sun (Asteraceae, Cichorieae): A new genus endemic to the Himalaya-Hengduan Mountains. Taxon. 60(6): 1678-1684.
254. Zhang, J. W., J. X. Yao, J. R. Chen, C. S. Li. 2010. A new species of Leptocycas (Zamiaceae) from the Upper Triassic sediments of Liaoning Province, China. Journal of Systematics and Evolution. 48(4): 286-301.
255. Zhang, M. D., Y. M. Shui, W. H. Chen, R. M. Zhang, G. Y. Li. 2010. Raphiocarpus maguanensis (Gesneriaceae), a new species from China. Annales Botanici Fennici. 47(1): 71-75.
256. Zhang, Q., G. X. Liu, Z. Y. Hu. 2011. Durinskia baltica (Dinophyceae), a newly recorded species and genus from China, and its systematics. Journal of Systematics and Evolution. 49(5): 476-485.
257. Zhang, Y. X., D. Z. Li. 2011. A new combination in Pseudosasa and a revised description of Indosasa hispida (Poaceae, Bambusoideae). Annales Botanici Fennici. 48(1): 79-83.
258. Zhang, Z. F., J. Li, J. An, P. G. Wang, H. Zhang. 2010. A New Species of Erigeron (Asteraceae) from Sichuan, China. Novon. 20(1): 117-119.
259. Zhao, Y. P., J. Paule, C. X. Fu, M. A. Koch. 2010. Out of China: Distribution history of Ginkgo biloba L. Taxon. 59(2): 495-504.
260. Zhao, Y., Y. Wu, L. Zhao. 2005. Caragana leduensis, a new species of Leguminosae from Qinghai, China. Willdenowia. 35(1): 155-157.
261. Zheng, X. L., F. G. Wang, R. J. Zhang, F. W. Xing. 2010. Pteris changjiangensis (Pteridaceae), a new species from Hainan Island, China. Annales Botanici Fennici. 47(2): 156-158.
262. Zhu, G., S. Chen. 1999. Cypripedium taibaiense (Orchidaceae), a new species from Shaanxi, China. Novon. 9(3): 454.
263. Zhu, X. 2004. Oxytropis lhasaensis (Fabaceae), a new species from Xizang (Tibet) in China, with supplementary notes on the section Sericopetala. Annals Botanici Fennici. 41(6): 495-497.
264. Zhu, X. 2005. A taxonomic revision of Tibetia (Leguminosae, Papilionoideae, Galegeae). Botanical Journal of the Linnean Society. 148(4): 475-488.
265. Zhu, X., H. Ohashi., L. Xu. 1999. A new species of Oxytropis (Leguminosae) from Xinjiang, China. The Journal of Japanese Botany. 74(3): 133-135.
266. Zhu, X., H. Ohashi., S. Li. 1999. A new species of Oxytropis DC. (Leguminosae) from Xizang, China. The Journal of Japanese Botany. 74(3): 130-132.
267. Zhu, X., H. Ohashi., Y. Deng. 1999. A new Oxytropis species (Leguminosae) from North and Northwest China. The Journal of Japanese Botany. 74(3): 127-129.
268. Zhu, X., H. Ohashi., Y. Deng. 1999. Four new species of Oxytropis DC. (Leguminosae) from Xinjiang, China. The Journal of Japanese Botany. 74(2): 63-71.
269. Zhu, X., Y. Du, H. Ohashi. 2000. A new Oxytropis species (Leguminosae) from Xinjiang in China. The Journal of Japanese Botany. 75(5): 289-291.
270. Zhu, X., Y. Du, H. Ohashi. 2002. A new species of Oxytropis (Leguminosae) from Xizang (Tibet) in China. Novon. 12(3): 430-432.
271. 《贵州植物志》编委会编. 1982. 贵州植物志 1卷. 贵州人民出版社, 贵阳.
272. 《全国中草药汇编》编写组编著. 1975. 全国中草药汇编 (上)册. 人民卫生出版社, 北京.
273. 《全国中草药汇编》编写组编著. 1978. 全国中草药汇编 (下)册. 人民卫生出版社, 北京.
274. 《西藏植物名录》编辑组. 1980. 西藏植物名录. 西藏自治区科学技术委员会印.
275. 艾训儒,易咏梅主编. 2005. 清江流域植被研究. 中国农业科学技术出版社, 北京.
276. 安定国主编. 2002. 甘肃省小陇山高等植物志. 甘肃民族出版社, 兰州.
277. 安徽经济植物志增修编写办公室,安徽省人民政府经济文化研究中心. 1990. 安徽经济植物志 (上)册. 安徽科学技术出版社, 合肥.
278. 安沫平主编. 2007. 河北农区野生植物资源图谱. 中国农业出版社, 北京.
279. 安争夕. 1999. 新疆植物志 5卷. 新疆科技卫生出版社, 乌鲁木齐.
280. 巴特尔编著. 2007. 鄂托克生物资源 (上)册. 内蒙古人民出版社, 呼和浩特.
281. 巴特尔编著. 2007. 鄂托克生物资源 (下)册. 内蒙古人民出版社, 呼和浩特.
282. 白佩瑜. 2000. 云南植物志 11卷. 科学出版社, 北京.
283. 柏松林,吴德成. 1994. 中国大兴安岭植物志. 黑龙江科学技术出版社, 哈尔滨.
284. 班程农主编. 2003. 贵州亚热带野生经济植物资源及利用. 贵州民族出版社, 贵阳.
285. 北京师范大学生物系植物组. 1975. 北京地区植物志-单子叶植物. 北京人民出版社, 北京.
286. 北京市中草药资源普查队编. 1972. 北京植物名录. 北京市中草药资源普查队, 北京.
287. 北镇农校植生教研组,谷奉天编写. 1980. 北镇种子植物名录. 山东省惠民地区科学技术情报研究所.
288. 毕润成. 2004. 山西省五鹿山自然保护区科学考察报告. 中国科学技术出版社, 北京.
289. 薄乖民,何录德. 2006. 甘肃洮河自然保护区科学考察报告. 西安地图出版社, 西安.
290. 蔡振聪主编. 1985. 台湾特用植物图鑑. 台湾省立博物馆.
291. 曹建军. 2007. 宁夏野生观赏植物. 宁夏人民出版社, 银川.
292. 曹善寿主编. 2003. 菜阳河自然保护区. 云南科学技术出版社, 昆明.
293. 曹善寿主编. 2004. 糯扎渡自然保护区. 云南科学技术出版社, 昆明.
294. 曹铁如,喻勋林,周建仁. 1996. 湖南保靖县白云山木本植物区系的研究. 中南林学院学报. 16(2):16-22.
295. 曹伟,李冀云,傅沛云,于兴华,朱彩霞. 2004. 大兴安岭植物区系与分布. 东北大学出版社, 沈阳.
296. 曹伟,李冀云. 2003. 长白山植物自然分布. 东北大学出版社, 沈阳.
297. 曹伟,李冀云. 2007. 小兴安岭植物区系与分布. 科学出版社, 北京.
298. 曹昀,黄兆辉,陈学林. 2003. 小陇山稀有濒危、特有植物的多样性及其保护. 甘肃科技. 19(4):77-79.
299. 陈邦杰,吴鹏程,裘佩熹,徐炳声,周纪纶著. 1965. 黄山植物的研究——苔藓、蕨类、种子植物的区系和地理. 上海科学技术出版社, 上海.
300. 陈德媛,陈家明主编. 1988. 贵州中草药名录. 贵州人民出版社, 贵阳.
301. 陈德昭. 1988. 中国植物志 39卷. 科学出版社, 北京.
302. 陈封怀,胡启明. 1989. 中国植物志 59(1)卷(册). 科学出版社, 北京.
303. 陈封怀,胡启明. 1990. 中国植物志 59(2)卷(册). 科学出版社, 北京.
304. 陈封怀主编. 1987. 广东植物志 1卷. 广东科学技术出版社, 广州.
305. 陈封怀主编. 1991. 广东植物志 2卷. 广东科学技术出版社, 广州.
306. 陈根荣编著. 2009. 浙江树木图鉴. 中国林业出版社, 北京.
307. 陈汉斌,郑亦津,李法曾. 1990. 山东植物志 (下)册. 青岛出版社, 青岛.
308. 陈汉斌. 1990. 山东植物志 (上)册. 青岛出版社, 青岛.
309. 陈焕镛,黄成就. 1998. 中国植物志 22卷. 科学出版社, 北京.
310. 陈焕镛主编. 1964. 海南植物志 1卷. 科学出版社, 北京.
311. 陈焕镛主编. 1965. 海南植物志 2卷. 科学出版社, 北京.
312. 陈冀胜,郑硕主编. 1987. 中国有毒植物. 科学出版社, 北京.
313. 陈家辉,杨勇. 2010. 羌塘草原植物识别手册. 云南科学技术出版社, 昆明.
314. 陈家宽主编. 2003. 上海九段沙湿地自然保护区科学考察集. 科学出版社, 北京.
315. 陈家瑞. 2000. 中国植物志 53(2)卷(册). 科学出版社, 北京.
316. 陈介. 1979. 中国植物志 58卷. 科学出版社, 北京.
317. 陈介. 1979. 中国植物志 5卷. 科学出版社, 北京.
318. 陈介. 1984. 中国植物志 53(1)卷(册). 科学出版社, 北京.
319. 陈里娥,刘永金,邢福武,曾振平,李文华,徐滔,曾亚碧. 2003. 梧桐山植物. 中国林业出版社, 北京.
320. 陈利君, 刘仲健. 2011. 深圳拟兰,中国兰科一新种(英文). 植物科学学报. 29(1): 38-41.
321. 陈利君, 饶文辉, 刘仲健. 2010. 石豆兰属(Bulbophyllum)(兰科)中两个种的新名称(英文). 武汉植物学研究. 28(6): 772.
322. 陈利君, 饶文辉, 刘仲健. 2010. 中国兰科石豆兰属(Bulbophyllum)二新种(英文). 武汉植物学研究. 28(4): 417-423.
323. 陈谦海,陈心启. 2003. 贵州兰科新资料. 植物分类学报. 41(3):263-266.
324. 陈谦海. 1985. 贵州鼠李科、漆树科的新植物. 云南植物研究. 7(4):413-416.
325. 陈谦海. 2004. 贵州植物志 10卷. 贵州科学技术出版社, 贵阳.
326. 陈蓉伯,田梠,王君,葛玉祥. 2001. 内蒙古红花尔基樟子松林国家级自然保护区综合考察报告. 内蒙古林业勘察设计院.
327. 陈榕生,叶秀端编. 1989. 厦门植物园植物名录. 北京出版社, 北京.
328. 陈三阳,王慷林. 2002. 云南棕榈藤新资料. 云南植物研究. 24(2):199-204.
329. 陈守良. 1990. 中国植物志 10(1)卷(册). 科学出版社, 北京.
330. 陈守良. 1997. 中国植物志 10(2)卷(册). 科学出版社, 北京.
331. 陈书坤,李德铢. 2006. 云南植物志 12卷. 科学出版社, 北京.
332. 陈书坤,王红. 2006. 云南植物志 16卷. 科学出版社, 北京.
333. 陈书坤. 1997. 云南植物志 8卷. 科学出版社, 北京.
334. 陈书坤. 1997. 中国植物志 43(3)卷(册). 科学出版社, 北京.
335. 陈书坤. 1999. 中国植物志 45(2)卷(册). 科学出版社, 北京.
336. 陈蜀江,侯平,李文华,李虎. 2006. 新疆夏尔希里自然保护区综合科学考察. 新疆科学技术出版社, 乌鲁木齐.
337. 陈松河, 郭惠珠, 黄克福. 2011. 中国竹亚科思劳竹属一新种——万石山思劳竹. 植物研究. 31(6): 641-643.
338. 陈涛,张宏达. 1995. 南岭植物区系地理学研究. 广西植物. 15(2):131-138.
339. 陈天虎主编. 2007. 恩施市维管束植物名录. 湖北科学技术出版社, 武汉.
340. 陈天云主编. 2010. 宁夏主要农业野生植物. 阳光出版社, 银川.
341. 陈伟烈,江明喜,赵常明,田自强. 2008. 三峡库区谷地的植物与植被. 中国水利水电出版社, 北京.
342. 陈伟烈,张喜群,梁松筠,金义兴,杨启修. 1994. 三峡库区的植物与复合农业生态系统. 科学出版社, 北京.
343. 陈伟球. 1999. 中国植物志 71(2)卷(册). 科学出版社, 北京.
344. 陈卫,胡东,付必谦. 2007. 北京湿地生物多样性研究. 科学出版社, 北京.
345. 陈蔚辉编著. 2008. 粤东植物名录. 华南理工大学出版社, 广州.
346. 陈文红,税玉民,郎楷永. 2003. 滇东南兰科无柱兰属一新种及其生物地理学意义. 云南植物研究. 25(5):521-524.
347. 陈文红,税玉民,喻智勇. 2003. 滇东南丫蕊花属(百合科)一新种及其生物地理学意义. 植物研究. 23(3):267-268.
348. 陈文红,税玉民. 2002. 滇东南大围山地区远志科一新种. 云南植物研究. 24(4):428-430.
349. 陈锡侨,吴七根. 2007. 澳门常见中草药 1册. 广东科学技术出版社, 广州.
350. 陈锡侨,吴七根. 2009. 澳门常见中草药 2册. 广东科学技术出版社, 广州.
351. 陈翔, 黄家勇, 谢华, 陈训. 2010. 中国贵州杜鹃属两新种(英文). 热带亚热带植物学报. 18(3): 259-263.
352. 陈心启,吉古和. 1998. 中国兰花全书. 中国林业出版社, 北京.
353. 陈心启,吉占和,罗毅波. 1999. 中国野生兰科植物彩色图鉴. 科学出版社, 北京.
354. 陈心启,李恒. 2002. 马尾南星--中国广西中部天南星科一新种. 云南植物研究. 24(5):607-608.
355. 陈心启,刘仲建. 2001. 兰科兜兰属一新种和一新变种. 植物分类学报. 39(5):455-458.
356. 陈心启,刘仲建. 2004. 中国兰科杓兰属一新种及一新变种. 云南植物研究. 26(4):382-384.
357. 陈心启,刘仲建. 2005. 杓兰属(兰科)中一个国产种类的新等级. 武汉植物学研究. 23(3):233-234.
358. 陈心启,罗毅波. 2002. 关于兰科*Eulophia hirsute* T.P.Lin的归属问题. 植物分类学报. 40(2):139-140.
359. 陈心启,罗毅波. 2004. 中国兰科植物一新种——云南巾唇兰. 植物分类学报. 42(5):457-459.
360. 陈心启. 1999. 中国植物志 18卷. 科学出版社, 北京.
361. 陈心启. 1999. 中国植物志 1卷. 科学出版社, 北京.
362. 陈艺林,杜玉芬. 2001. 西藏勾儿茶属一新种. 植物分类学报. 39(1):73-74.
363. 陈艺林,石铸. 1989. 贵州植物志 9卷. 四川民族出版社, 贵阳.
364. 陈艺林,石铸. 1999. 中国植物志 78(2)卷(册). 科学出版社, 北京.
365. 陈艺林. 1982. 中国植物志 48(1)卷(册). 科学出版社, 北京.
366. 陈艺林. 1999. 中国植物志 77(1)卷(册). 科学出版社, 北京.
367. 陈艺林. 2001. 中国植物志 47(2)卷(册). 科学出版社, 北京.
368. 陈勇. 2003. 大丰糜鹿国家级自然保护区可持续发展研究. 南京林业大学(博士论文).
369. 陈余钊,渊锡东,管加兴. 2006. 温州湿地资源. 中国林业出版社, 北京.
370. 陈玉峰著. 1997. 台湾植被志 2(下)册. 晨星出版社, 台中.
371. 陈玉峰著. 1998. 台湾植被志 3(下)册. 前卫出版社, 台北.
372. 陈征海. 2010. 天目山植物志 2卷. 浙江大学出版社, 杭州.
373. 陈子牛,翟书华,孟学会. 2009. 云南石林县资源植物. 云南科学技术出版社, 昆明.
374. 陈宗团,王博,朱开建. 2008. 厦门大屿岛白鹭自然保护区. 海洋出版社, 北京.
375. 成克武,张铁民,刘敬文主编. 2010. 唐山南湖湿地公园生物多样性及生态规划. 中国林业出版社, 北京.
376. 成晓. 2005. 云南植物志 21卷. 科学出版社, 北京.
377. 诚静容,黄普华. 1999. 中国植物志 45(3)卷(册). 科学出版社, 北京.
378. 程用谦. 1982. 中国植物志 20(1)卷(册). 科学出版社, 北京.
379. 程用谦. 1996. 中国植物志 79卷. 科学出版社, 北京.
380. 崔波,李服,马杰主编. 2008. 郑州植物志. 中国科学技术出版社, 北京.
381. 崔国发,邢韶华,赵勃. 2008. 北京山地植物和植被保护研究. 中国林业出版社, 北京.
382. 崔国发,邢韶华. 2009. 北京喇叭沟门自然保护区综合科学考察报告. 中国林业出版社, 北京.
383. 崔鸿宾. 1998. 中国植物志 42(2)卷(册). 科学出版社, 北京.
384. 崔乃然,宋宗仁,安沙舟,刘霞,孙连起. 1990. 新疆草地植物名录. 新疆人民出版社, 乌鲁木齐.
385. 崔乃然. 1996. 新疆植物志 6卷. 新疆科技卫生出版社, 乌鲁木齐.
386. 大连地区植物志编写组和大连自然博物馆. 1982. 大连地区植物志 (上)册. 大连理工大学出版社, 大连.
387. 大连地区植物志编写组和大连自然博物馆. 1982. 大连地区植物志 (下)册. 大连理工大学出版社, 大连.
388. 大连地区植物志编写组和大连自然博物馆. 1982. 大连地区植物志 (中)册. 大连理工大学出版社, 大连.
389. 戴好富,梅文莉主编. 2007. 海南药用植物现代研究. 中国科学技术出版社, 北京.
390. 戴伦凯,梁松筠. 2000. 中国植物志 12卷. 科学出版社, 北京.
391. 戴天澍,敬根才,张清华,宋朝枢主编. 1991. 鸡公山木本植物图鉴. 中国林业出版社, 北京.
392. 戴晓勇, 安明态, 杨成华. 2010. 贵州新分布植物. 贵州科学. 28(1): 89-92.
393. 单人骅,佘孟兰. 1979. 中国植物志 55(1)卷(册). 科学出版社, 北京.
394. 单人骅,佘孟兰. 1985. 中国植物志 55(2)卷(册). 科学出版社, 北京.
395. 单人骅,佘孟兰. 1992. 中国植物志 55(3)卷(册). 科学出版社, 北京.
396. 党坤良,李登武,张怀科,侯琳. 2009. 陕西黄柏塬自然保护区综合科学考察. 西北农林科技大学出版社, 咸阳.
397. 党坤良,孟中华,宋小民. 2004. 陕西子午岭自然保护区综合科学考察. 西北农林科技大学出版社, 咸阳.
398. 党荣理,潘晓玲. 2001. 西北干旱荒漠区植物区系的特有现象分析. 植物研究. 21(4):519-526.
399. 邓洪平,主审,字发主编. 2009. 西南大学校园高等植物学习指南. 西南师范大学出版社, 重庆.
400. 邓家刚,韦松基主编. 2008. 桂药原色图鉴. 上海科学技术出版社, 上海.
401. 邓莉兰,魏开云,樊国盛. 2003. 云南槭属植物一新种. 云南植物研究. 25(2):197-198.
402. 邓敏, 曹明, 席世丽, 曹小燕. 2011. 倒卵叶青冈——广西壳斗科一新记录种. 广西植物. 31(5): 575-577.
403. 邓敏, 李谦盛, 曹明, 席世丽. 2011. 雷公青冈和滇南青冈学名订正. 广西植物. 31(2): 148-152.
404. 邓云飞, Do, Van, HAI, Dzuong, Duc, HUYEN. 2010. 越南爵床科一新记录属——恋岩花属(英文). 热带亚热带植物学报. 18(1): 40-42.
405. 邓云飞, J. R.I.WOOD, 高春明. 2010. 中国马蓝属(爵床科)新组合和新分类群(英文). 热带亚热带植物学报. 18(5): 469-484.
406. 邓云飞, 郝振萍. 2011. 非洲爵床科一新组合——短穗观音草(英文). 热带亚热带植物学报. 19(4): 327.
407. 狄维忠主编. 1987. 贺兰山维管植物. 西北大学出版社, 西安.
408. 第二军医大学药学系生药学教研室编著. 1960. 中国药用植物图鉴. 上海科学技术出版社, 上海.
409. 刁正谷. 1990. 中国水生杂草. 重庆出版社, 重庆.
410. 丁宝章,王遂义,高增义主编. 1981. 河南植物志 1卷. 河南人民出版社, 郑州.
411. 丁宝章,王遂义主编. 1988. 河南植物志 2卷. 河南科学技术出版社, 郑州.
412. 丁宝章,王遂义主编. 1997. 河南植物志 3卷. 河南科学技术出版社, 郑州.
413. 丁宝章,王遂义主编. 1998. 河南植物志 4卷. 河南科学技术出版社, 郑州.
414. 丁炳录,康健主编. 1994. 大兴安岭资源植物. 气象出版社, 北京.
415. 丁汉波,孔繁升,石溥,许建飞,吴经伦,吴鹏程,李登科,林鹏,林来官,赵修复,赵昭昞,张永田,黄邦侃,黄年来. 1993. 武夷山自然保护区科学考察报告集. 福建科学技术出版社, 福州.
416. 丁丽惠主编. 2008. 浙江省丽水市林木种质资源. 中国农业出版社, 北京.
417. 东北林学院林学系. 1984. 东北林学院凉水自然保护区基础资料. 东北林业大学出版社, 哈尔滨.
418. 东北林学院林学系. 1984. 东北林学院帽儿山实验林场基础资料. 东北林业大学出版社, 哈尔滨.
419. 董汉飞,骆世明,刘琦,黄泮光,刘尚仁,叶锦昭,卢如秀,冯志坚,覃朝锋,李贞,黎华寿,莫熙穆. 1995. 鉴江流域水资源生态环境与经济发展. 中山大学出版社发行, 广州.
420. 董建新,孙春岐主编. 2007. 承德生物资源. 辽宁民族出版社, 沈阳.
421. 董金廷,侯学良编著. 1998. 淮北植物. 中国环境科学出版社, 北京.
422. 董旭,张胜邦,张更权主编. 2007. 青海祁连山自然保护区科学考察集. 中国林业出版社, 北京.
423. 董玉琛,郑殿升主编. 2005. 国家重点保护农业野生植物要略. 气象出版社, 北京.
424. 杜诚, 岳晓娜, 盛国文, 常朝阳, 徐朗然. 2010. 单小叶黄耆——内蒙古豆科一新记录种. 西北植物学报. 30(5): 1057-1059.
425. 杜品编著. 2006. 青藏高原甘南藏药植物志. 甘肃科学技术出版社, 兰州.
426. 杜怡斌. 2000. 河北野生资源植物志. 河北大学出版社, 保定.
427. 杜怡斌主编. 1992. 河北野生植物. 河北大学出版社, 保定.
428. 段富强,虎卫军,田进生,高启平,刘康林,马安义,张秀红,苏进禄,张荣,牛国璋,锁鹏珠,田建彪,张骞,田喜荣. 2005. 宁夏南华山自然保护区综合科学考察报告. 宁夏人民出版社, 银川.
429. 段林东, 林祁, 杨志荣, 邵青. 2011. 中国12省荨麻科植物分布新记录. 西北植物学报. 31(5): 1050-1052.
430. 樊国盛,陈书坤. 1997. 云南雀梅藤属二新种. 云南植物研究. 19(1):37-39.
431. 樊守金, 张学杰, 李法曾. 2010. 羊茅属(禾本科)新类群. 广西植物. 30(1): 26-28.
432. 樊守金,胡泽绪主编. 2003. 崂山植物志 (上)卷. 科学出版社, 北京.
433. 樊守金,胡泽绪主编. 2003. 崂山植物志 (下)卷. 科学出版社, 北京.
434. 范航清,陈光华,何斌原,莫竹承. 2005. 山口红树林滨海湿地与管理. 海洋出版社, 北京.
435. 方鼎,梁定仁. 2000. 广西胡颓子属(胡颓子科)三新种. 植物分类学报. 38(3):289-293.
436. 方鼎,谢志明. 2002. 广西茜草科植物三新种. 植物分类学报. 40(2):154-158.
437. 方鼎. 1999. 广西樟科植物两新种. 植物分类学报. 37(6):595-597.
438. 方鼎主编. 1993. 广西中药资源名录. 广西民族出版社, 南宁.
439. 方辉云,吴国凤,汪建恩,向德全编. 1988. 恩施市药用植物名录. 恩施市药品检验所汇编.恩施市卫生局.
440. 方精云,赵淑清,唐志尧. 2006. 长江中游湿地生物多样性保护的生态学基础. 高等教育出版社, 北京.
441. 方瑞征. 1991. 中国植物志 57(3)卷(册). 科学出版社, 北京.
442. 方瑞征. 1999. 中国植物志 57(1)卷(册). 科学出版社, 北京.
443. 方瑞征. 2003. 云南植物志 15卷. 科学出版社, 北京.
444. 方文培,胡文光. 1990. 中国植物志 56卷. 科学出版社, 北京.
445. 方文培,张泽荣. 1983. 中国植物志 52(2)卷(册). 科学出版社, 北京.
446. 方文培. 1981. 四川植物志 1卷. 四川人民出版社, 成都.
447. 方文培. 1981. 中国植物志 46卷. 科学出版社, 北京.
448. 方云亿主编. 1992. 浙江植物志 5卷. 浙江科学技术出版社 , 杭州.
449. 方志先,廖朝林主编. 2006. 湖北恩施药用植物志 (上)册. 湖北科学技术出版社, 武汉.
450. 方志先,廖朝林主编. 2006. 湖北恩施药用植物志 (下)册. 湖北科学技术出版社, 武汉.
451. 方志先,赵晖,赵敬华主编. 2007. 土家族药物志 (上)册. 中国医药科学技术出版社, 北京.
452. 方志先,赵晖,赵敬华主编. 2007. 土家族药物志 (下)册. 中国医药科学技术出版社, 北京.
453. 冯国楣,杨增宏主编. 1999. 中国杜鹃花 3册. 科学出版社, 北京.
454. 冯国楣. 1984. 中国植物志 49(2)卷(册). 科学出版社, 北京.
455. 冯国楣主编. 1988. 中国杜鹃花 1册. 科学出版社, 北京.
456. 冯国楣主编. 1992. 中国杜鹃花 2册. 科学出版社, 北京.
457. 冯显逵等编著. 1979. 宁夏六盘山贺兰山木本植物图鉴. 宁夏人民出版社, 银川.
458. 冯缨,严成,尹林克. 2003. 新疆植物特有种及其分布. 西北植物学报. 23(2):263-273.
459. 冯自诚,孙学刚,张承维编. 1989. 迭部树木简志. 天则出版社, 杨陵.
460. 冯自诚,徐梦龙主编. 1994. 甘南树木图志. 甘肃科学技术出版社, 兰州.
461. 符国瑷,洪小江. 2004. 海南岛润楠属一新种. 植物研究. 24(3):259-260.
462. 福建省林业厅编. 1997. 龙栖山自然保护区科学考察综合报告. 内部资料未正式出版.
463. 福建省商业厅编. 1960. 福建野生经济植物. 福建人民出版社, 福州.
464. 傅大立, 李炳仁, 傅建敏, 李建宏, 杨绍彬, 周道顺, 刘梦培. 2010. 中国杏属一新种. 植物研究. 30(1): 1-3.
465. 傅大立, 李芳文, 孙金花, 任继红. 2010. 四川玉兰属两新种. 植物研究. 30(4): 385-389.
466. 傅大立, 赵天榜, 陈志秀, 陈树森. 2010. 湖北玉兰属两新种. 植物研究. 30(6): 641-644.
467. 傅坤俊,傅竞秋,陈彦生. 2000. 黄土高原植物志 1卷. 科学出版社, 北京.
468. 傅坤俊,傅竞秋. 1983 秦岭植物志 1(4)卷(册). 科学出版社, 北京.
469. 傅坤俊,王作宾. 1960. 西北野生有用植物手册 4册. 陕西人民出版社, 西安.
470. 傅坤俊,肖崇礼,王作宾,唐昌林,郭本兆,傅竞秋,何善宝. 1959. 西北野生有用植物手册 1册. 陕西人民出版社, 西安.
471. 傅坤俊,肖崇礼,王作宾,唐昌林,郭本兆,傅竞秋,何善宝. 1959. 西北野生有用植物手册 2册. 陕西人民出版社, 西安.
472. 傅坤俊,张振万. 1981 秦岭植物志 1(3)卷(册). 科学出版社, 北京.
473. 傅坤俊,张志英. 1985 秦岭植物志 1(5)卷(册). 科学出版社, 北京.
474. 傅坤俊. 1993. 中国植物志 42(1)卷(册). 科学出版社, 北京.
475. 傅坤俊主编. 1989. 黄土高原植物志 5卷. 科学技术文献出版社, 北京.
476. 傅坤俊主编. 1992. 黄土高原植物志 2卷. 中国林业出版社, 北京.
477. 傅坤俊主编. 2000. 黄土高原植物志 1卷. 科学出版社, 北京.
478. 傅立国,陈潭清,郎楷永,洪涛,林祁,李勇. 2001. 中国高等植物 7卷. 青岛出版社, 青岛.
479. 傅立国,陈潭清,郎楷永,洪涛,林祁,李勇. 2002. 中国高等植物 13卷. 青岛出版社, 青岛.
480. 傅立国,陈潭清,郎楷永,洪涛,林祁,李勇. 2003. 中国高等植物 5卷. 青岛出版社, 青岛.
481. 傅立国,陈潭清,郎楷永,洪涛,林祁,李勇. 2003. 中国高等植物 6卷. 青岛出版社, 青岛.
482. 傅立国,陈潭清,郎楷永,洪涛,林祁,李勇. 2004. 中国高等植物 10卷. 青岛出版社, 青岛.
483. 傅立国,陈潭清,郎楷永,洪涛,林祁,李勇. 2005. 中国高等植物 11卷. 青岛出版社, 青岛.
484. 傅立国,陈潭清,郎楷永,洪涛,林祁,李勇. 2008. 中国高等植物 2卷. 青岛出版社, 青岛.
485. 傅立国,陈潭清,郎楷永,洪涛,林祁,李勇. 2009. 中国高等植物 12卷. 青岛出版社, 青岛.
486. 傅立国,陈潭清,郎楷永,洪涛,林祁,李勇. 2012. 中国高等植物 1卷. 青岛出版社, 青岛.
487. 傅立国,陈潭清,郎楷永,洪涛,林祁. 2000. 中国高等植物 4卷. 青岛出版社, 青岛.
488. 傅立国,陈潭清,郎楷永,洪涛,林祁. 2001. 中国高等植物 8卷. 青岛出版社, 青岛.
489. 傅立国,陈潭清,郎楷永,洪涛. 1999. 中国高等植物 9卷. 青岛出版社, 青岛.
490. 傅立国,陈潭清,浪楷永,洪涛. 2000. 中国高等植物 3卷. 青岛出版社, 青岛.
491. 傅沛云主编. 1998 东北草本植物志 12卷. 科学出版社, 北京.
492. 傅书遐,傅坤俊. 1984. 中国植物志 34(1)卷(册). 科学出版社, 北京.
493. 傅书遐. 1957. 中国主要植物图说蕨类植物门. 科学出版社, 北京.
494. 傅书遐主编,中国科学院武汉植物研究所. 2002. 湖北植物志 3卷. 湖北科学技术出版社, 武汉.
495. 傅书遐主编,中国科学院武汉植物研究所. 2002. 湖北植物志 4卷. 湖北科学技术出版社, 武汉.
496. 甘啟良. 2005. 竹溪植物志. 湖北科学技术出版社, 武汉.
497. 甘肃师范大学生物系编. 1962. 甘肃经济植物. 甘肃人民出版社, 兰州.
498. 高宝莼,谭仲明. 1989. 四川植物志 9卷. 四川民族出版, 成都.
499. 高宝莼主编. 1992. 四川省重点保护珍贵树木图志. 四川民族出版社, 成都.
500. 高春明, 邓云飞. 2010. 爵床科一新组合——菲律宾爵床(英文). 热带亚热带植物学报. 18(3): 257-258.
501. 高继银,帕克斯,杜跃强主编. 2005. 山茶属植物主要原种彩色图集. 浙江科学技术出版社, 杭州.
502. 高乞, 许为斌. 2011. 贵州苦苣苔科一新记录属——弥勒苣苔属. 西北植物学报. 31(4): 858-860.
503. 高谦,曹同. 2000. 云南植物志 17卷. 科学出版社, 北京.
504. 高松. 2008. 辽南地区药用植物图鉴. 科学出版社, 北京.
505. 高维衡编著. 1998. 崆峒山植物志. 甘肃文化出版社, 兰州.
506. 高学斌,康永祥. 2007. 陕西皇冠山省级自然保护区综合科学考察. 陕西科学技术出版社, 西安.
507. 郜国玉,王玉生,王新民. 1995. 豫北黄河故道湿地鸟类自然保护区科学考察与研究. 黄河水利出版社, 郑州.
508. 葛斌杰, 田旗, 魏宇昆, 王川, 崔浪军. 2011. 陕西省鼠尾草属植物地理分布新记录. 西北植物学报. 31(7): 1487-1489.
509. 葛继稳,胡鸿兴,李博. 2009. 湖北木林子自然保护区森林生物多样性研究. 科学出版社, 北京.
510. 耿伯介,王正平. 1996. 中国植物志 9(1)卷(册). 科学出版社, 北京.
511. 耿伯介,王正平. 1996. 中国植物志 9(1)卷. 科学出版社, 北京.
512. 耿以礼. 1959. 中国主要植物图说禾本科. 科学出版社, 北京.
513. 龚才. 1996. 赤习水林区综合科学考察集. 贵州科学技术出版社, 贵阳.
514. 苟光前, 王晓宇, 熊源新. 2010. 石蝴蝶属一新种——黄斑石蝴蝶. 植物研究. 30(4): 394-396.
515. 古晓林主编. 2001. 宁夏云雾山自然保护区科学考察与管理文集. 宁夏人民出版社, 银川.
516. 谷粹芝. 1999. 中国植物志 52(1)卷(册). 科学出版社, 北京.
517. 官少飞,张天火. 1989. 江西水生高等植物. 上海科学技术出版社, 上海.
518. 管开云,田代科. 2000. 云南秋海棠属三新种. 云南植物研究. 22(2):129-134.
519. 管中天. 1983. 四川植物志 2卷. 四川人民出版社, 成都.
520. 广东省林业厅技术室编. 1957. 广东主要经济树木 1分册, 广东省林业厅编印.
521. 广东省植物研究所编. 1974. 海南植物志 3卷. 科学出版社, 北京.
522. 广东省植物研究所编. 1977. 海南植物志 4卷. 科学出版社, 北京.
523. 广西大瑶山自然资源综合考察队著. 1988. 广西大瑶山自然资源考察. 学林出版社, 上海.
524. 广西花坪林区综合考察队编. 1986. 广西花坪林区综合考察报告. 山东科学技术出版社, 济南.
525. 广西南宁树木园编. 1988. 广西南宁树木园树木名录. 广西南宁树木园出版.
526. 广西植物编委会. 1988. 广西植物增刊一广西弄岗自然保护区综合考察报告. 广西植物.
527. 广西植物研究所. 1971. 广西植物名录 2(双子叶植物)册. 广西植物研究所(未正式出版物), 南宁.
528. 广西植物研究所. 1973. 广西植物名录 3(单子叶植物)册. 广西植物研究所(未正式出版物), 南宁.
529. 广西植物研究所编著,钟济新主编. 1982. 广西石灰岩石山植物图谱. 广西人民出版社, 南宁.
530. 广西壮族自治区海岸带和海涂资源综合调查领导小组. 1986. 广西壮族自治区海岸带和海涂资源综合调查报告第七卷(植被和林业).
531. 广西壮族自治区医药研究所药用植物园编. 1974. 广西医药研究所药用植物园药用植物名录. 广西壮族自治区医药研究所药用植物园, 南宁.
532. 贵州省环境保护局,六盘水市环境保护局编. 1990. 六盘水野钟黑叶猴自然保护区科学考察集. 贵州民族出版社, 贵阳.
533. 贵州省环境保护局编. 1990. 赤水桫椤自然保护区科学考察集. 贵州民族出版社发行, 贵阳.
534. 贵州省植物园编. 1989. 贵州省植物园植物名录. 贵州人民出版社, 贵阳.
535. 郭本兆. 1987. 中国植物志 9(3)卷(册). 科学出版社, 北京.
536. 郭本兆主编. 1987. 青海经济植物志. 青海人民出版社, 西宁.
537. 郭城孟. 1999. 台湾维管束植物简志 1卷. 承峰美术印刷股份有限公司, 台北.
538. 郭文场,张德群,衣茂功主编. 1993. 东北野生可食植物. 中国林业出版社, 北京.
539. 郭文艺. 2007. 陕西摩天岭自然保护区综合科学考察与研究. 陕西科学技术出版社, 西安.
540. 郭英荣,江波,王英永,乐新贵,葛刚. 2010. 江西阳际峰自然保护区综合科学考察报告. 科学出版社, 北京.
541. 郭永兵, 夏念和. 2010. 国产牡竹属野龙竹和版纳甜龙竹的订正. 热带亚热带植物学报. 18(2): 133-136.
542. 郭玉生主编. 2010. 太行山树木志. 天津科学技术出版社, 天津.
543. 国家环境保护局,中国科学院植物研究所编 1987. 中国珍稀濒危保护植物名录 1册. 科学出版社, 北京.
544. 国家林业局调查规划设计院,宁夏罗山自然保护区管理所. 2000. 宁夏罗山自然保护区科学考察集. 内部资料未正式出版.
545. 国家林业局中南调查规划设计院,广西大明山自然保护区管理处. 2000. 广西大明山自然保护区综合科学考察. 内部资料未正式出版.
546. 国家林业局中南林业调查规划设计院,湖南六步溪自然保护区管理局. 2006. 湖南六步溪自然保护区综合科学考察.
547. 国家怡,贾文泽. 1999. 黄河三角洲生物多样性研究. 青岛出版社, 青岛.
548. 哈斯巴根,苏亚拉图编著. 2008. 内蒙古野生蔬菜资源及其民族植物学研究. 科学出版社, 北京.
549. 韩恩俊,赵七元,张嘉仁编著. 1991. 北方天然香料色素植物资源开发. 农村读物出版社, 北京.
550. 韩剑准. 2001. 海南尖峰岭自然保护区综合科学考察报告.
551. 韩玉铃,岳春雷,李贺鹏,邵利萍,陈友吾,叶碎高,严齐斌,赵聚国. 2009. 河道生态建设－河道植物资源. 中国水利水电出版社, 北京.
552. 杭州植物园. 1976. 杭州植物园栽培植物名录. 杭州植物园.
553. 郝振萍, 邓云飞, T. F.DANIEL. 2010. 中国爵床科一新组合——尾叶爵床(英文). 热带亚热带植物学报. 18(5): 485-487.
554. 何飞, 刘兴良, 刘世荣, 蔡蕾, 文智猷, 潘红丽, 樊华, 胡宗达. 2010. 卧龙自然保护区种子植物新记录. 四川林业科技. 31(5): 79-82.
555. 何家庆. 2001. 皖北资源植物志. 中国农业出版社, 北京.
556. 何景,曾沧江. 1978. 中国植物志 54卷. 科学出版社, 北京.
557. 何顺志. 1996. 贵州绞股蓝属(葫芦科)一新种. 植物分类学报. 34(2):207-209.
558. 何廷农. 1988. 中国植物志 62卷. 科学出版社, 北京.
559. 何廷农. 1999. 四川植物志 15卷. 四川民族出版社, 成都.
560. 何友均著. 2008. 三江源自然保护区森林植物多样性及其保护研究. 中国林业出版社, 北京.
561. 何云核. 2002. 安徽冬青属一新种. 植物分类学报. 40(4):380-382.
562. 贺家仁,刘志斌. 2008. 甘孜州高等植物. 四川科学技术出版社, 成都.
563. 贺家仁主编. 1993. 甘孜州树木. 四川科学技术出版社, 成都.
564. 贺利中,刘仁林主编. 2010. 江西七溪岭自然保护区科学考察及生物多样性研究. 江西科学技术出版社, 南昌.
565. 贺士元,刑其华,尹祖棠,江先甫. 1993. 北京植物志 (上)册. 北京出版社, 北京.
566. 贺士元,刑其华,尹祖棠,江先甫. 1993. 北京植物志 (下)册. 北京出版社, 北京.
567. 贺士元主编. 1986. 河北植物志 1卷. 河北科学技术出版社, 石家庄.
568. 贺士元主编. 1989 河北植物志 2卷. 河北科学技术出版社, 石家庄.
569. 贺士元主编. 1991. 河北植物志 3卷. 河北科学技术出版社, 石家庄.
570. 黑龙江省野生经济植物图志编辑委员会编. 1963. 黑龙江省野生经济植物图志. 黑龙江人民出版社, 哈尔滨.
571. 红河苏铁自然保护区考察队. 1996. 云南红河苏铁自然保护区综合考察报告. 内部资料未正式出版.
572. 洪德元. 1983. 中国植物志 73(2)卷(册). 科学出版社, 北京.
573. 洪起平,丁平,丁炳扬主编. 2007. 浙江凤阳山-百山祖国家级自然保护区凤阳山自然资源考察与研究. 中国林业出版社, 北京.
574. 洪洋,吴俊军编著. 2000. 辉河自然保护区综合考察. 内蒙古文化出版社, 呼伦贝尔.
575. 侯海宫,吕益涛,苏耀海,徐培元. 2000. 苍耳属一新变种--一室苍耳. 植物研究. 20(3):249-250.
576. 侯宽昭主编. 1956. 广州植物志. 科学出版社, 北京.
577. 侯学良,税玉民. 2003. 中国哥纳香属(番荔枝科)一新种. 云南植物研究. 25(3):258-260.
578. 侯学煜. 1988. 中国自然地理：植物地理 (下)册. 科学出版社, 北京.
579. 侯元同, 王继梅. 2010. 中国菊属的一些新组合. 广西植物. 30(6): 815-817.
580. 胡浩, 金效华, 孙琦, 孙学刚. 2010. 中国兰科植物一新记录种(英文). 热带亚热带植物学报. 18(4): 401-402.
581. 胡嘉琪,梁师文. 1996. 黄山植物. 复旦大学出版社, 上海.
582. 胡嘉琪. 2002. 中国植物志 70卷. 科学出版社, 北京.
583. 胡琳贞,方明渊. 1994. 中国植物志 57(2)卷(册). 科学出版社, 北京.
584. 胡明芳, 黎维英, 刘江枫, 陈丽爱, 周敏. 2010. 福建兰科新记录属—叉柱兰属. 福建林业科技. 37(3): 106-107.
585. 胡启明, 郝刚, 邓云飞. 2011. 黑色报春花学名订正(英文). 热带亚热带植物学报. 19(3): 222-224.
586. 胡启明主编. 2003. 香港稀有及珍贵植物. 香港特别行政区政府渔农自然护理署.
587. 湖北赛武当自然保护区科考组. 2008. 湖北赛武当自然保护区科学考察报告.
588. 湖北省植物研究所编著. 1976 湖北植物志 1卷. 湖北人民出版社, 武汉.
589. 湖南省林业调查规划设计院. 2000. 湖南小溪自然保护区自然资源综合科学考察报告.
590. 湖南省林业调查规划设计院. 2001. 湖南炎陵桃源洞自然保护区自然资源综合科学考察报告. 内部资料未正式出版.
591. 湖南省森林资源管理保护局,湖南省永州市林业局. 1998. 湖南永州都庞岭自然保护区自然资源综合科学考察报告. 内部资料未正式出版.
592. 湖南省中医药研究所编. 1972. 湖南药物志 2辑. 湖南人民出版社, 长沙.
593. 湖南舜皇山自然保护区综合考察队. 2006. 湖南舜皇山自然保护区综合科学考察报告集. 国家林业局中南林业调查规划设计院.
594. 扈明阁主编. 1990. 赤峰草地. 农业出版社, 北京.
595. 华西野生植物保护实验中心,都江堰市林业局. 1991. 中国都江堰市植物名录. 内部资料未正式出版.
596. 黄成就. 1997. 中国植物志 43(2)卷(册). 科学出版社, 北京.
597. 黄桂林. 2009. 青海可鲁克湖-托素湖湿地自然保护区生物多样性. 北京出版社, 北京.
598. 黄金非,王国杰. 1992. 嫩江流域中药资源及应用. 黑龙江科学技术出版社, 哈尔滨.
599. 黄金玲,蒋得斌. 2002. 广西猫儿山自然保护区综合科学考察. 湖南科学技术出版社, 长沙.
600. 黄金祥,李信,钱进源主编. 1996. 塞罕坝植物志. 中国科学技术出版社, 北京.
601. 黄立铨,石成亮,王才明. 1986. 桂林植物园栽培植物名录. 广西植物研究所(内部资料).
602. 黄明忠, 刘芝龙, 王青隆, 黄法祥, 杨光穗, 尹俊梅, 莫饶. 2011. 中国石斛兰属(兰科)一新记录种——广坝石斛兰(英文). 热带亚热带植物学报. 19(6): 558-560.
603. 黄威廉. 1989. 贵州植物志 6卷. 四川民族出版社, 成都.
604. 黄威廉主编. 1989. 贵州珍稀濒危植物. 中国环境科学出版社, 北京.
605. 黄俞淞, 陆茂新, 杨金财, 许为斌. 2011. 中国双唇兰属(兰科)一新记录种——中越双唇兰. 广西植物. 31(5): 578-580.
606. 黄俞淞, 许为斌, 叶晓霞, 刘演. 2010. 中国广西石灰岩地区苦苣苔科—新种——鹿寨唇柱苣苔(英文). 热带亚热带植物学报. 18(2): 137-139.
607. 黄宗国. 2004. 海洋河口湿地生物多样性. 海洋出版社, 北京.
608. 黄宗国主编. 2006. 厦门湾物种多样性. 海洋出版社, 北京.
609. 辉朝茂,杜凡. 1998. 中国横断山区竹亚科箭竹属新分类群. 植物研究. 18(3):257-274.
610. 辉朝茂,薛纪如. 1992. 中国竹亚科梨藤竹属的研究. 植物分类学报. 30(2):163-168.
611. 辉朝茂,杨宇明. 1999. 中国高黎贡山国家自然保护区竹亚科新分类群. 林业科学. 35(1):43-46.
612. 吉林波罗湖自然保护区科学考察组. 2009. 吉林波罗湖自然保护区科学考察报告.
613. 吉林省野生经济植物志编辑委员会编著. 1961. 吉林省野生经济植物志. 吉林省人民出版社, 长春.
614. 吉占和. 1999. 中国植物志 19卷. 科学出版社, 北京.
615. 贾大柱主编. 1993. 安徽省天马自然保护区科学考察报告. 金寨县林业局编印.
616. 贾恢先,孙学刚. 2005. 中国西北内陆盐地植物图谱. 中国林业出版社, 北京.
617. 贾良智,周俊主编. 1987. 中国油脂植物. 科学出版社, 北京.
618. 江海声,黄文忠. 1998. 海南省南湾自然保护区及其周边生物多样性. 广东科学技术出版社, 广州.
619. 江海声. 2006. 海南吊罗山生物多样性及其保育. 广东科学技术出版社, 广州.
620. 江苏省植物研究所编. 1977. 江苏植物志 (上)册. 江苏人民出版社 , 南京.
621. 江苏省植物研究所编. 1982. 江苏植物志 (下)册. 江苏科学技术出版社 , 南京.
622. 姜传义. 2000. 中国杀虫植物志. 新疆科技卫生出版社, 乌鲁木齐.
623. 姜登伟编著. 1999. 麦秀林区植物简志. 青海人民出版社, 西宁.
624. 姜景民. 1990. 浙江省槭属植物区系与分布的初步研究. 林业科学研究. 3(6):624-628.
625. 蒋谦才,李镇魁主编. 2008. 中山野生植物. 广东科学技术出版社, 广州.
626. 蒋谦才. 2009. 中山市维管植物名录. 广东科学技术出版社, 广州.
627. 蒋日红, 吴望辉, 农东新, 许为斌. 2011. 中国石灰岩地区霉草科一新记录种——星状霉草. 广西植物. 31(1): 9-10.
628. 蒋英,李秉滔. 1977. 中国植物志 63卷. 科学出版社, 北京.
629. 蒋英,李秉滔. 1979. 中国植物志 30(2)卷(册). 科学出版社, 北京.
630. 蒋政权. 2005. 北京市顺义区杨镇苇塘湿地生物多样性与生态旅游规划研究. 首都师范大学(硕士论文).
631. 蒋志刚. 2002. 天坑地缝风景名胜区生物多样性研究与保护. 中国林业出版社, 北京.
632. 蒋志刚. 2005. 陕西青木川自然保护区的生物多样性. 清华大学出版社, 北京.
633. 蒋志刚. 2009. 江西桃红岭梅花鹿国家级自然保护区生物多样性研究. 清华大学出版社, 北京.
634. 蒋志刚主编. 2006. 陕西老县城自然保护区的生物多样性. 清华大学出版社, 北京.
635. 交通部天津水运工程科学研究所. 2007. 天津古海岸与湿地国家级自然保护区综合考察报告. 交通部天津水运工程科学研究所,天津古海岸与湿地国家级自然保护区管理处.
636. 焦瑜,李承森. 2007. 中国云南蕨类植物(新编). 科学出版社, 北京.
637. 金连成,邱英杰. 2004. 辽宁野生动植物和湿地资源. 东北林业大学出版社, 哈尔滨.
638. 金水虎, 马丹丹, 欧丹燕, 李根有. 2010. 普陀山4个植物新类群. 西北植物学报. 30(8): 1701-1702.
639. 金水虎. 2010. 天目山植物志 1卷. 浙江大学出版社, 杭州.
640. 金孝锋,翁东明主编. 2009. 清凉峰植物. 浙江大学出版社, 杭州.
641. 金孝锋. 2010. 天目山植物志 4卷. 浙江大学出版社, 杭州.
642. 金效华,吉占和,覃海宁,萧丽萍,蹇平. 2002. 贵州兰科植物增补. 植物分类学报. 40(1):82-88.
643. 金效华,钱义咏. 2001. 中国石斛属一新种. 植物分类学报. 39(3):269-271.
644. 金效华,赵晓冬,施晓春. 2009. 高黎贡山原生兰科植物. 科学出版社, 北京.
645. 金振洲主编. 2009. 云南高原湿地植物的分类与地理生态特征汇编. 科学出版社, 北京.
646. 靳淑英. 1999. 中国高等植物模式标本汇编(补编). 中国林业出版社, 北京.
647. 靳淑英编. 1994. 中国高等植物模式标本汇编. 科学出版社, 北京.
648. 靳淑英编. 2007. 中国高等植物模式标本汇编(补编二). 科学出版社, 北京.
649. 九江市林业局. 2004. 九江市林业局野生植物标本馆馆藏标本志要.
650. 孔冬梅,张义贤,张峰,谢树莲编著. 2008. 山西庞泉沟自然保护区植物学. 中国农业科学技术出版社, 北京.
651. 孔红. 2002. 天水地区种子植物野外实习指导. 兰州大学出版社, 兰州.
652. 孔宪武,简焯坡. 1979. 中国植物志 25(2)卷(册). 科学出版社, 北京.
653. 孔宪武,王文采. 1989. 中国植物志 64(2)卷(册). 科学出版社, 北京.
654. 孔宪武著. 1962. 兰州植物通志. 甘肃人民出版社, 兰州.
655. 孔宪需. 2001. 中国植物志 5(2)卷(册). 科学出版社, 北京.
656. 孔宪需著. 1988. 四川植物志 6卷. 四川科学技术出版社, 成都.
657. 宽甸县白石砬子自然保护区,中国科学院林业土壤研究所. 1984. 辽宁省白石砬子植物名录. 白石砬子自然保护区.
658. 匡可任,李沛琼. 1979. 中国植物志 21卷. 科学出版社, 北京.
659. 匡可任,路安民. 1978. 中国植物志 67(1)卷(册). 科学出版社, 北京.
660. 旷建军主编. 2008. 南岳衡山维管束植物总览. 湖南科学技术出版社, 长沙.
661. 蓝崇钰,王勇军. 2001. 广东内伶仃岛自然资源与生态研究. 中国林业出版社, 北京.
662. 蓝开敏. 1988. 贵州植物志 5卷. 四川民族出版社, 成都.
663. 郎楷永,冯志舟,李渤生. 1997. 中国高山花卉. 中国世界语出版社, 北京.
664. 郎楷永,萧丽萍. 2002. 国产开唇兰属(兰科)一新种. 植物分类学报. 40(2):164-166.
665. 郎楷永. 1999. 中国植物志 17卷. 科学出版社, 北京.
666. 乐天宇,徐纬英著. 1957. 陕甘宁盆地植物志. 中国林业出版社, 北京.
667. 雷明德编. 1999. 陕西植被. 科学出版社, 北京.
668. 雷耘. 2010. 武当山南麓植物地理学研究. 湖北科学技术出版社, 武汉.
669. 黎斌, 李思锋, 张莹, 陈昊, 张瑞博. 2010. 秦岭植物区系一新记录科——茶茱萸科. 西北植物学报. 30(1): 203-204.
670. 黎光南主编. 1990. 云南中药志 1卷. 云南科学技术出版社, 昆明.
671. 黎明主编. 2009. 河南燃料油植物资源与应用. 河南大学出版社, 开封.
672. 黎兴江. 2002. 云南植物志 18卷. 科学出版社, 北京.
673. 黎兴江. 2005. 云南植物志 19卷. 科学出版社, 北京.
674. 李安仁,徐国士. 2005. 中国蓼科植物图谱. 海洋出版社, 北京.
675. 李安仁. 1998. 中国植物志 25(1)卷(册). 科学出版社, 北京.
676. 李保国,何鹏举主编. 2007. 陕西周至国家级自然保护区生物多样性. 陕西科学技术出版社, 西安.
677. 李丙贵主编. 2000. 湖南植物志 2卷. 湖南科学技术出版社, 长沙.
678. 李丙贵主编. 2004. 湖南植物志 1卷. 湖南科学技术出版社, 长沙.
679. 李秉涛. 1993. 亚洲番荔枝科植物新资料. 广西植物. 13(4):311-315.
680. 李秉滔. 1994. 中国植物志 44(1)卷(册). 科学出版社, 北京.
681. 李采兰,杨济中,高东藩,陈家明,李朝斗. 1965. 贵州民间药物(第一辑). 贵州人民出版社, 贵阳.
682. 李操主编. 2010. 四川白羊自然保护区综合科学考察. 四川科学技术出版社, 成都.
683. 李朝銮. 1998. 中国植物志 48(2)卷(册). 科学出版社, 北京.
684. 李成岐,米泰岩,周翰儒. 2007. 安徽经济植物志 (下)册. 安徽科学技术出版社, 合肥.
685. 李春光,王永明,玄武编. 1990. 长白山东北部野生经济植物志. 延边人民出版社, 延边.
686. 李德铢,薛纪如. 1995. 贡山竹属—我国云南竹亚科一新属. 植物分类学报. 33(6):597-601.
687. 李根有, 陈征海, 胡军飞, 金水虎, 马丹丹, 欧丹燕. 2010. 发现于浙江普陀山岛的2个植物新变种(英文). 浙江林学院学报. 26(6): 908-909.
688. 李根有,陈敬佑主编. 2007. 浙江林学院植物园植物名录. 中国林业出版社, 北京.
689. 李根有,颜福彬主编. 2007. 浙江温岭植物资源. 中国农业出版社, 北京.
690. 李光照,李虹. 2006. 中国南方花卉. 上海科学技术出版社, 上海.
691. 李光照编著. 2008. 中国广西杜鹃花. 上海科学技术出版社, 上海.
692. 李桂林主编. 1998. 赛罕乌拉自然保护区综合科学考察集. 内蒙古赛罕乌拉自然保护区.
693. 李恒,郭辉军,刀志灵主编. 2000. 高黎贡山植物. 科学出版社, 北京.
694. 李恒,李嵘,刀志灵. 2003. 独龙虾脊兰(兰科)的合格发表. 植物分类学报. 41(3):267-270.
695. 李恒,王仲朗,龙春林. 1999. 高黎贡山南星属(天南星科)新资料. 云南植物研究. 21(11):55-60.
696. 李恒,钟兴耀主编. 2010. 中国大山包黑颈鹤自然保护区植物. 科学出版社, 北京.
697. 李恒. 1993. 独龙江地区植物. 云南科学技术出版社, 昆明.
698. 李恒. 2003. 云南植物志 14卷. 科学出版社, 北京.
699. 李恒. 2009. 云南湿地植物名录. 科学出版社, 北京.
700. 李宏伟主编. 2003. 白马雪山国家级自然保护区. 云南民族出版社, 昆明.
701. 李虹, 蒋水元, 孙世荣, 李光照. 2010. 值得关注的广西新记录和新组合植物. 广西植物. 30(3): 295-296.
702. 李惠民主编. 1990. 山西省经济植物志. 中国林业出版社, 北京.
703. 李吉宁, 蒋旭亮, 李志刚, 陈天云, 张志耘. 2011. 清水河枸杞,宁夏茄科一新种. 广西植物. 31(4): 427-429.
704. 李继钧,武云飞,朱松泉,李德浩,王祖祥,黄永昭,郑英敏,刘尚武,潘锦堂,张盍曾,颜京松,叶沧江,王基琳,印象初,郑昌琳. 1979. 西藏阿里地区动植物考察报告. 科学出版社, 北京.
705. 李冀云. 2003. 中国东北蒿属光砂蒿的两个新变种. 植物研究. 23(4):388-389.
706. 李冀云主编. 2004. 东北草本植物志 9卷. 科学出版社, 北京.
707. 李嘉珏,谢忙义主编. 2001. 甘肃太统－崆峒山自然保护区科学考察集. 中国林业出版社, 北京.
708. 李建,黄翔. 2006. 北京市永定河门头沟段湿地自然保护区科学考察报告. 中国科学技术出版社, 北京.
709. 李建东,王贵卿. 1993. 吉林省长岭腰井子羊草草原自然保护区资料汇编. 吉林省长岭腰井子羊草草原自然保护区资料汇编编委会.
710. 李建清,李长贵,徐何方,胡小绿,陈小芬. 2001. 楠溪江珍稀濒危特有植物调查研究. 浙江林业科技. 21(5):33-34.
711. 李剑武, 陶国达, 刘强. 2011. 中国兰科一新记录属——袋距兰属(英文). 植物分类与资源学报. 33(6): 643-644.
712. 李捷,李锡文. 1996. 中国樟科植物拾零. 云南植物研究. 18(1):53-55.
713. 李金水主编. 2006. 黄山珍稀植物. 中国林业出版社, 北京.
714. 李朗, 李捷, 李锡文. 2011. 国产樟科楠属五种植物之分类修订. 植物分类与资源学报. 33(2): 157-160.
715. 李连芳. 2005. 围场红松洼国家级自然保护区主要生物资源及多样性编目研究. 科学出版社, 北京.
716. 李林英主编. 2006. 山西植物种质资源研究. 中国林业出版社, 北京.
717. 李琳, 叶德平, 李剑武, 邢福武. 2011. 中国石豆兰属(兰科)二新记录(英文). 热带亚热带植物学报. 19(2): 149-151.
718. 李琳, 叶德平, 邢福武. 2010. 中国蝴蝶兰属(兰科)一新记录(英文). 广西植物. 30(3): 292-294.
719. 李佩洪, 吴银明. 2010. 四川野生花椒资源调查. 四川林业科技. 31(6): 82-85.
720. 李庆奎主编. 2009. 天津八仙山国家级自然保护区生物多样性考察. 天津科学技术出版社, 天津.
721. 李仁郁. 1991. 黑龙江省西部草原常见植物检索. 黑龙江科学技术出版社, 哈尔滨.
722. 李荣伟,王乐辉,陈秀明,何飞著. 2007. 青藏高原东缘野生观赏植物. 四川科学技术出版社, 成都.
723. 李世全主编. 1987. 秦岭巴山天然药物志. 陕西科学技术出版社, 西安.
724. 李书春,李秾,吴诗华,刘秀梅,吴泽民,何明勋,吴诚和. 1983. 安徽木本植物. 安徽科学技术出版社, 合肥.
725. 李书春,吴诗华,吴泽民,何家庆,刘秀梅,张定成,黄世华,王德群,孙乐知,钱啸虎,郭新弧,武祖发,董双庆,卢心固,李真,吴诚和,李秾,陈仁钧,叶如欣,邵建章,刘启新,张智,吴学良,强胜. 1990. 安徽植物志 3卷. 中国展望出版社, 北京.
726. 李书春主编. 1995. 中条山树木志. 中国林业出版社, 北京.
727. 李书心,刘淑贞,曹伟. 2005. 东北草本植物志 8卷. 科学出版社, 北京.
728. 李书心主编. 1988. 辽宁植物志 (上)册. 辽宁科学技术出版社, 沈阳.
729. 李书心主编. 1992. 辽宁植物志 (下)册. 辽宁科学技术出版社, 沈阳.
730. 李树刚,梁畴芬. 1991. 广西植物志 1卷. 广西科学技术出版社, 南宁.
731. 李树刚. 1987. 中国植物志 60(1)卷(册). 科学出版社, 北京.
732. 李树刚. 1995. 中国植物志 41卷. 科学出版社, 北京.
733. 李树刚主编. 2005. 广西植物志 2卷. 广西科学技术出版社, 南宁.
734. 李思锋,黎斌. 2009. 秦巴山区野生观赏植物. 陕西科学技术出版社, 西安.
735. 李思健著. 2007. 枣庄野生植物资源. 山东大学出版社, 济南.
736. 李添地,周锦超,吴兆洪. 2003. 香港植物志(蕨类植物门). 嘉道理农场暨植物园公司.
737. 李卫忠,赵鹏祥,贾生平. 2006. 陕西延安黄龙山褐马鸡自然保护区综合科学考察. 西北农林科技大学出版社, 咸阳.
738. 李锡文,税玉民. 2004. 云南樟科油丹属一新种——黄连山油丹. 植物分类学报. 42(6):551-554.
739. 李锡文,祝正银. 1992. 四川植物志 10卷. 四川民族出版社, 成都.
740. 李锡文. 1982. 中国植物志 31卷. 科学出版社, 北京.
741. 李锡文. 1990. 中国植物志 50(2)卷(册). 科学出版社, 北京.
742. 李小伟, 曹兵, 秦伟春, 姬学龙, 施兴文, 王平. 2010. 宁夏被子植物分布新资料. 西北植物学报. 30(5): 1060-1062.
743. 李小伟, 曹兵, 秦伟春, 姬学龙, 施兴文, 王平. 2011. 宁夏大戟科植物分布新资料. 西北植物学报. 31(1): 195-196.
744. 李晓东, 昝艳燕, 刘宏涛, 李建强. 2011. 川鄂獐耳细辛一新变型(英文). 西北植物学报. 31(11): 1-2.
745. 李晓民. 2005. 黑龙江流域湿地及保护. 东北林业大学出版社, 哈尔滨.
746. 李新华. 2010. 中国小檗属二新异名. 广西植物. 30(4): 440-442.
747. 李延辉,裴盛基,赵世望编. 1984. 西双版纳植物名录. 云南民族出版社, 昆明.
748. 李延生主编. 1990. 辽宁树木志. 中国林业出版社, 北京.
749. 李扬汉主编. 1998. 中国杂草志. 中国农业出版社, 北京.
750. 李耀阶主编. 1987. 青海木本植物志. 青海人民出版社, 西宁.
751. 李以镔主编. 1995. 江西野生观赏植物. 中国林业出版社, 北京.
752. 李应武,侯惠敏,苏炳勋,卓正大,张国梁,孙继周,林璋德,刘迺发,高兆宁,许成,张嘏,包天祥. 1988. 六盘山自然保护区科学考察. 宁夏人民出版社, 银川.
753. 李永芳主编. 2008. 北京珍稀濒危及常见野生植物. 中国三峡出版社, 北京.
754. 李永康. 1988. 贵州植物志 8卷. 四川民族出版社, 贵阳.
755. 李永康主编. 1986. 贵州植物志 2卷. 贵州人民出版社, 贵阳.
756. 李战刚,党坤良,李登武. 2005. 陕西天华山自然保护区综合科学考察与研究. 陕西科学技术出版社, 西安.
757. 李战刚,康克功,吴振海主编. 2008. 陕西平河梁省级自然保护区综合科学考察与生物多样性研究. 陕西科学技术出版社, 西安.
758. 李战刚,任毅,王学杰. 2006. 陕西长青国家级自然保护区综合科学考察报告. 陕西科学技术出版社, 西安.
759. 李珍. 2007. 安徽鹞落坪自然保护区维管植物多样性研究. 安徽大学(硕士论文).
760. 李振基,吴小平,陈小麟,刘长明. 2009. 江西九岭山自然保护区综合科学考察报告. 科学出版社, 北京.
761. 李振宇,邱小敏. 1993. 广西九万山植物资源考察报告. 中国林业出版社, 北京.
762. 李振宇,石雷. 2007. 峨眉山植物. 北京科学技术出版社, 北京.
763. 李振宇,王印政主编. 2004. 中国苦苣苔科植物. 河南科学技术出版社, 郑州.
764. 李振宇主编. 1994. 龙栖山植物. 中国科学出版社, 北京.
765. 李正春,刘守仁,缪礼维编著. 1990. 新疆石河子饲用植物志. 新疆人民出版社, 乌鲁木齐.
766. 李忠平,刘增力,遇宝成. 2006. 吉林汪清东北红豆杉自然保护区生物多样性. 北京出版社, 北京.
767. 廉永善,孙坤主编. 2005. 甘肃植物志 2卷. 甘肃科学技术出版社, 兰州.
768. 梁建平主编. 2001. 广西珍稀濒危树种. 广西科学技术出版社, 南宁.
769. 梁立中, 聂思铭, 梁盛华, 梁盛海. 2010. 中国东北圆柏属新品种——直立型偃柏. 植物研究. 30(5): 632-633.
770. 梁显有主编. 1991. 西藏山南土地资源. 中国农业科学技术出版社, 北京.
771. 辽宁省老秃顶子自然保护区管理处,辽宁省林业厅. 1996. 老秃顶子自然保护区科学考察报告集. 辽宁林业胶版印刷厂印刷.
772. 辽宁省林业土壤研究所编著. 1975. 东北草本植物志 3卷. 科学出版社, 北京.
773. 辽宁省林业土壤研究所编著. 1976. 东北草本植物志 11卷. 科学出版社, 北京.
774. 辽宁省林业土壤研究所编著. 1976. 东北草本植物志 5卷. 科学出版社, 北京.
775. 辽宁省林业土壤研究所编著. 1977. 东北草本植物志 6卷. 科学出版社, 北京.
776. 廖文波,叶常镜,王晓明,常弘,赖燕玲,金建华,梁嘉声,崔大方,仲铭锦,曾曙才,贾凤龙,庄平弟,李贞,徐晓晖,孙延军,丁明艳,于法钦,凡强,付勉兴,刘汶,刘郁,刘蔚秋,羊海军,朱世杰,许涵,张兵兰,张锦新,张碧胜,杨秀敏,杨际明,邱智明,陈考科,陈素芳,罗连,孟昭祥,贾莲莲,黄伟结,黄康有,蒋昕. 2007. 深圳马峦山郊野公园生物多样性及其生态可持续发展. 科学出版社, 北京.
777. 林春蕊, 谢彦军, 梁树朝, 刘演. 2011. 中国玉叶金花属一新记录种——长瓣玉叶金花. 热带亚热带植物学报. 19(6): 554-557.
778. 林来官,张永田. 1995. 福建植物志 6卷. 福建科学技术出版社, 福州.
779. 林来官. 1982. 福建植物志 1卷. 福建科学技术出版社, 福州.
780. 林来官. 1985. 福建植物志 2卷. 福建科学技术出版社, 福州.
781. 林来官. 1988. 福建植物志 3卷. 福建科学技术出版社, 福州.
782. 林来官. 1990. 福建植物志 4卷. 福建科学技术出版社, 福州.
783. 林来官. 1993. 福建植物志 5卷. 福建科学技术出版社, 福州.
784. 林来官. 1998. 中国植物志 50(1)卷(册). 科学出版社, 北京.
785. 林媚珍,卓正大. 1996. 广东南昆山植物区系的基本特征. 华南师范大学学报(自然科学版). 2:74-79.
786. 林媚珍著. 2008. 广东南昆山生态旅游区的森林植被及生态旅游. 气象出版社, 北京.
787. 林木木,阙天福,郑世群. 2005. 福建润楠属一新种. 植物研究. 25(1):5-6.
788. 林培钧,崔乃然主编. 2000. 天山野果林资源－－伊犁野果林综合研究. 中国林业出版社, 北京.
789. 林鹏. 1999. 福建省南靖南亚热带雨林自然保护区科学考察报告. 厦门大学出版社, 厦门.
790. 林鹏. 2001. 福建梁野山自然保护区综合科学考察报告. 厦门大学出版社, 厦门.
791. 林鹏. 2001. 福建漳江口红树林湿地自然保护区综合科学考察报告. 厦门大学出版社, 厦门.
792. 林鹏主编. 2002. 福建天宝岩自然保护区综合科学考察报告. 厦门大学出版社, 厦门.
793. 林萍,田昆,杨宇明,刘朝蓬,吴荣,普晓兰,谢凌雁,潘耕耘. 2009. 云南高原湖滨常见湿地植物图鉴. 科学出版社, 北京.
794. 林泉主编. 1993. 浙江植物志 7卷. 浙江科学技术出版社, 杭州.
795. 林镕,陈艺林. 1985. 中国植物志 74卷. 科学出版社, 北京.
796. 林镕,林有润. 1991. 中国植物志 76(2)卷(册). 科学出版社, 北京.
797. 林镕,刘尚武. 1989. 中国植物志 77(2)卷(册). 科学出版社, 北京.
798. 林镕,石铸. 1983. 中国植物志 76(1)卷(册). 科学出版社, 北京.
799. 林镕,石铸. 1987. 中国植物志 78(1)卷(册). 科学出版社, 北京.
800. 林镕,石铸. 1997. 中国植物志 80(1)卷(册). 科学出版社, 北京.
801. 林镕. 1979. 中国植物志 75卷. 科学出版社, 北京.
802. 林斯超,李财德,陈叔宣主编. 1996. 牡丹峰国家级自然保护区自然资源与保护. 东北林业大学出版社, 哈尔滨.
803. 林业部调查规划设计院,广东大雾岭自然保护区. 1997. 广东大雾岭国家级自然保护区综合考察报告文集. 林业部调查规划设计院,广东大雾岭自然保护区.
804. 林英主编. 1990. 井冈山自然保护区考察研究. 新华出版社, 北京.
805. 林英主编. 1993. 江西植物志 1卷. 江西科学技术出版社, 南昌.
806. 林英主编. 2004. 江西植物志 2卷. 中国科学技术出版社, 北京.
807. 林尤兴. 2000. 中国植物志 6(2)卷(册). 科学出版社, 北京.
808. 林有润,葛学军. 1999. 中国植物志 80(2)卷(册). 科学出版社, 北京.
809. 林有润,孙秀殿. 2001. 菊科一新属.管花蒲公英属和菊科舌状花亚科的补充记载. 植物研究. 21(2):175-176.
810. 林有润主编. 2005. 菊科植物论文集. 科学教育出版社, 香港.
811. 刘宝权, 张芬耀, 谢长明, 谢文远, 马丹丹. 2011. 浙江堇菜属植物新记录. 西北植物学报. 31(5): 1053-1054.
812. 刘春延,赵亚民,刘海莹,张向忠. 2010. 塞罕坝森林植物图谱. 中国林业出版社, 北京.
813. 刘东,杨峻山. 1999. 中国特有植物三叶青化学成分的研究. 中国中药杂志. 24(10):611-612.
814. 刘恩德,彭华著. 2010. 永德大雪山种子植物区系和森林植被研究. 云南科学技术出版社, 昆明.
815. 刘广全,等著. 2005. 黄土高原植被构建效应. 中国科学技术出版社, 北京.
816. 刘国钧,沈观冕,毛祖美,冷巧珍. 1984. 新疆药用植物志 3册. 新疆人民出版社, 乌鲁木齐.
817. 刘国强,王志臣,马国青. 2006. 北京云蒙山自然保护区生物多样性. 北京出版社, 北京.
818. 刘国柱,周正仁,欧润芝. 1967. 台湾野生可食植物.
819. 刘海桑, 池敏杰. 2010. 中国分类学文献中*Swietenia mahagoni*之订正. 植物研究. 30(6): 660-663.
820. 刘海桑. 2010. 中国分类学文献中*Livistona saribus*之订正. 武汉植物学研究. 28(2): 239-242.
821. 刘海桑. 2011. 《Flora of China》中蒲葵属之分类订正(英文). 植物研究. 31(6): 644-648.
822. 刘和义,杨远波,吕胜由,施炳霖编著. 2000. 台湾维管束植物简志 3卷, 中华民国行政院农业委员会出版发行.
823. 刘厚培,朱景郊主编. 1992. 南岭山区自然资源开发利用. 科学出版社, 北京.
824. 刘家宜. 1995. 天津植物名录. 天津教育出版社, 天津.
825. 刘家宜. 2004. 天津植物志. 天津科学技术出版社, 天津.
826. 刘建林,罗强,赵丽华. 2010. 四川攀西种子植物 2卷. 清华大学出版社, 北京.
827. 刘建林,罗支铁,杨红. 2000. 爵床属一新变型. 植物研究. 20(3):254
828. 刘建林,孟秀祥,冯金朝. 2007. 四川攀西种子植物. 清华大学出版社, 北京.
829. 刘剑秋. 2006. 闽江河口湿地研究. 科学出版社, 北京.
830. 刘立品. 1998. 子午岭木本植物志. 兰州大学出版社, 兰州.
831. 刘利柱. 2000. 黑龙江省富锦七星河三环泡湿地自然保护区综合考察报告. 佳木斯市环保局,富锦市环保局.
832. 刘莲芬,钱关泽,黄勇,郭善利. 2001. 棘豆属一新种. 植物研究. 21(3):338-339.
833. 刘濂主编. 1996. 河北植被. 科学出版社, 北京.
834. 刘良源,曾新方,李志萌. 2003. 东江源区三百山野生生物资源保护与开发研究. 江西科学技术出版社, 南昌.
835. 刘亮. 2002. 中国植物志 9(2)卷(册). 科学出版社, 北京.
836. 刘迺发,马崇玉主编. 1997. 尕海-则岔自然保护区. 中国林业出版社, 北京.
837. 刘迺发,孙继周主编. 2003. 麦积山风景名胜区生物多样性. 天水市申请世杰遗产办公室,兰州大学生命科学学院.
838. 刘迺发,杨增武. 2006. 甘肃安西极旱荒漠国家级自然保护区二期综合科学考察. 兰州大学出版社, 兰州.
839. 刘迺发主编. 2001. 甘肃敦煌自然保护区科学考察. 中国林业出版社, 北京.
840. 刘品辉编. 1985. 井冈山植物名录 1册, 江西省井冈山自然保护区(内部资料).
841. 刘逎发,郝耀明,吴洪斌主编. 2005. 宁夏沙坡头国家级自然保护区综合科学考察. 兰州大学出版社发行, 兰州.
842. 刘逎发,张惠昌,窦志刚主编. 2010. 甘肃盐池湾国家级自然保护区综合科学考察. 兰州大学出版社, 兰州.
843. 刘荣成,林竑斌编著. 2008. 惠安常见树木. 中国林业出版社, 北京.
844. 刘尚武主编. 1996. 青海植物志 3卷. 青海人民出版社, 西宁.
845. 刘尚武主编. 1997. 青海植物志 1卷. 青海人民出版社, 西宁.
846. 刘尚武主编. 1999. 青海植物志 2卷. 青海人民出版社, 西宁.
847. 刘尚武主编. 1999. 青海植物志 4卷. 青海人民出版社, 西宁.
848. 刘少英,章小平,曾宗永主编. 2007. 九寨沟自然保护区生物多样性. 四川科学技术出版社, 成都.
849. 刘慎谔主编 1958. 东北草本植物志 1卷. 科学出版社, 北京.
850. 刘慎谔主编 1959. 东北草本植物志 2卷. 科学出版社, 北京.
851. 刘慎谔主编 1980. 东北草本植物志 4卷. 科学出版社, 北京.
852. 刘慎谔主编. 1981. 东北草本植物志 7卷. 科学出版社, 北京.
853. 刘胜祥,瞿建平. 2003. 湖北星斗山自然保护区科学考察集. 湖北科学技术出版社, 武汉.
854. 刘胜祥,瞿建平主编. 2006. 湖北七姊妹山自然保护区科学考察与研究报告. 湖北科学技术出版社, 武汉.
855. 刘诗峰,张坚. 2003. 佛坪自然保护区生物多样性研究与保护. 陕西科学技术出版社, 西安.
856. 刘世彪,张代贵,姜业芳,林永蕙编著. 2006. 湘西地区常见植物鉴别. 湖南科学技术出版社, 长沙.
857. 刘世龙,赵见明. 2009. 云南德宏州高等植物 (上)册. 科学出版社, 北京.
858. 刘世龙,赵见明. 2009. 云南德宏州高等植物 (下)册. 科学出版社, 北京.
859. 刘寿养. 2004. 广西荛花属(瑞香科)一新变种——黄药白花荛花. 植物分类学报. 42(3):265-267.
860. 刘棠瑞. 1960. 台湾木本植物图志 (上), 国立台湾大学农学院印行.
861. 刘棠瑞. 1962. 台湾木本植物图志 (下), 国立台湾大学农学院.
862. 刘天慰,岳建英主编. 2004. 山西植物志 4卷. 中国科学技术出版社, 北京.
863. 刘天慰,岳建英主编. 2004. 山西植物志 5卷. 中国科学技术出版社, 北京.
864. 刘天慰,张云峰主编. 1992. 太原植物志 2卷. 中国科学技术出版社, 北京.
865. 刘天慰主编 1992. 山西植物志 1卷. 中国科学技术出版社, 北京.
866. 刘天慰主编 1998. 山西植物志 2卷. 中国科学技术出版社, 北京.
867. 刘天慰主编. 1990. 太原植物志 1卷. 学术书刊出版社, 北京.
868. 刘天慰主编. 2000. 山西植物志 3卷. 中国科学技术出版社, 北京.
869. 刘贤德,杨全生. 2006. 祁连山生物多样性研究. 中国科学技术出版社, 北京.
870. 刘小林, 杜诚, 常朝阳, 徐朗然. 2010. 豆科植物阿拉善黄耆的分类订正. 西北植物学报. 30(2): 417-419.
871. 刘小明,郭英荣,刘仁林主编. 2010. 江西齐云山自然保护区综合科学考察集. 中国林业出版社, 北京.
872. 刘新民,赵哈林,赵爱芬主编. 1996. 科尔沁沙地风沙环境与植被. 科学出版社, 北京.
873. 刘信中,樊三宝,胡斌华主编. 2006. 江西南矶山湿地自然保护区综合科学考察. 中国林业出版社, 北京.
874. 刘信中,方福生. 2001. 江西武夷山自然保护区科学考察集. 中国林业出版社, 北京.
875. 刘信中,傅清. 2006. 江西马头山自然保护区科学考察与稀有植物群落研究. 中国林业出版社, 北京.
876. 刘信中,王琅主编. 2010. 江西省庐山自然保护区生物多样性考察与研究. 科学出版社, 北京.
877. 刘信中,吴和平. 2005. 江西官山自然保护区科学考察与研究. 中国林业出版社, 北京.
878. 刘信中,肖忠优,马建华主编. 2002. 江西九连山自然保护区科学考察与森林生态系统研究. 中国林业出版社, 北京.
879. 刘信中主编. 2000. 江西湿地. 中国林业出版社, 北京.
880. 刘信中主编. 2000. 江西桃红岭梅花鹿保护区. 中国林业出版社, 北京.
881. 刘岩,周荣俊. 2008. 黑龙江大兴安岭林木种质资源图鉴. 东北林业大学出版社, 哈尔滨.
882. 刘演,韦毅刚,唐赛春. 2006. 中国广西苦苣苔科一新种——灵川小花苣苔. 植物分类学报. 44(3):340-344.
883. 刘业经,吕福原,欧辰雄. 1988. 台湾树木志. 国立中兴大学农学院出版委员会.
884. 刘毅,张绍云. 2010. 滇南地区药用植物. 云南科学技术出版社, 昆明.
885. 刘毅,郑进主编. 2008. 香格里拉民族药图鉴. 云南科学技术出版社, 昆明.
886. 刘毅. 2009. 泸西药用植物图鉴. 云南民族出版社, 昆明.
887. 刘媖心,杨喜林,姚育英,张国梁. 1985. 中国沙漠植物志 1卷. 科学出版社, 北京.
888. 刘媖心,杨喜林,姚育英. 1987. 中国沙漠植物志 2卷. 科学出版社, 北京.
889. 刘媖心,杨喜林,姚育英. 1992. 中国沙漠植物志 3卷. 科学出版社, 北京.
890. 刘玉壶,罗献瑞. 1985. 中国植物志 47(1)卷(册). 科学出版社, 北京.
891. 刘玉壶. 1996. 中国植物志 30(1)卷(册). 科学出版社, 北京.
892. 刘增力,岳建兵,宋铁芳. 2009. 辽宁章古台自然保护区生物多样性. 北京出版社, 北京.
893. 刘振良,周学仁. 2001. 山西树木志. 中国林业出版社, 北京.
894. 刘正宇. 2010. 重庆金佛山生物资源名录. 西南师范大学出版社, 重庆.
895. 刘仲建,陈心启. 2004. 多根兰,中国云南兰科一新种. 云南植物研究. 26(3):297-298.
896. 刘仲建,陈心启. 2004. 四川兰科一新种——西昌风兰. 云南植物研究. 26(3):299-300.
897. 刘仲健,陈心启,茹正忠. 2005. 麻栗坡蝴蝶兰——中国兰科一新种. 云南植物研究. 27(1):37-38.
898. 刘仲健,陈心启,茹正忠. 2005. 中国云南兰科一新种——昌宁兰. 云南植物研究. 27(4):378-380.
899. 刘仲健,陈心启,茹正忠. 2006. 兰属腋花组若干种类的研究. 植物分类学报. 44(2):178-183.
900. 刘仲健,陈心启,茹正忠. 2006. 五裂红柱兰——云南兰科一新种. 云南植物研究. 28(1):13-14.
901. 刘仲健,陈心启,张建勇. 2002. 兜兰属宽瓣亚属(广义)的被充研究. 植物分类学报. 40(4):364-370.
902. 刘仲健,陈心启,张建勇. 2003. 云南兰科—新种—中华火焰兰. 武汉植物学研究. 21(1):37-39.
903. 刘仲健,陈心启. 2000. 福兰,中国云南兰科一新种. 植物分类学报. 38(6):570-572.
904. 刘仲健,陈心启. 2000. 窄瓣兜兰,中国云南兰科一新种. 植物分类学报. 38(5):464-466.
905. 刘仲健,陈心启. 2001. 玲珑兜兰,中国云南兰科一新种. 植物分类学报. 39(2):156-159.
906. 刘仲健,陈心启. 2002. 密毛兜兰,中国云南兰科一新种. 植物分类学报. 40(3):283-285.
907. 刘仲健,陈心启. 2002. 少叶硬叶兰,中国兰科一新种. 武汉植物学研究. 20(5):350-352.
908. 刘仲健,陈心启. 2002. 中国云南兰科一新种—二叶兰. 武汉植物学研究. 20(6):421-423.
909. 刘仲健,陈心启. 2004. 细花兰,中国云南兰科一新种. 武汉植物学研究. 22(6):500-502.
910. 刘仲健,陈心启. 2004. 夏凤兰——云南兰科一新种. 武汉植物学研究. 22(4):323-325.
911. 刘仲健,张建勇,李利强. 2003. 金蝉兰,中国云南兰科一新种. 武汉植物学研究. 21(4):316-318.
912. 刘仲健,张建勇. 2000. 同色兜兰及其亲缘群的研究. 云南植物研究. 22(4):390-394.
913. 刘仲健,张建勇. 2000. 心启兜兰,兜兰属宽瓣亚属的一个新增种. 植物分类学报. 38(5):467-470.
914. 刘仲健,张建勇. 2001. 金豆兜兰,中国西南部兰科兜兰属一新变种. 植物分类学报. 39(5):459-460.
915. 刘仲健,张建勇. 2001. 中国云南兜兰属(兰科)一新种. 植物分类学报. 39(6):568-570.
916. 刘仲健,张建勇. 2002. 多叶兜兰,云南兜兰属一新种. 云南植物研究. 24(2):191-192.
917. 龙春林,周翊兰编著. 2004. 口腔本草. 云南科学技术出版社, 昆明.
918. 龙溪－虹口国家级自然保护区总体规则项目组. 2000. 龙溪－虹口国级自然保护区总体规划.
919. 庐山植物园. 1982. 庐山植物名录. 江西九江第一印刷厂, 九江.
920. 陆玲娣,黄淑美. 1995. 中国植物志 35(1)卷(册). 科学出版社, 北京.
921. 陆益新,黄广宾. 1989. 广西特有植物的研究(续一). 广西植物. 9(2):119-186.
922. 陆益新,黄广宾. 1989. 广西特有植物的研究. 广西植物. 9(1):37-58.
923. 路安民,陈书坤. 1986. 中国植物志 73(1)卷(册). 科学出版社, 北京.
924. 吕光辉,杨建军,孙凡,张宝忠,舒慧章. 2008. 新疆克拉玛依生物多样性及保护. 新疆人民出版社, 乌鲁木齐.
925. 罗建, 汪书丽, 郑维列. 2010. 西藏十字花科新记录植物. 西北植物学报. 30(11): 2343-2344.
926. 罗建, 汪书丽. 2011. 西藏睡菜科一新记录属——莕菜属. 广西植物. 31(2): 153-154.
927. 罗强, 蔡光泽, 刘建林, 王同军. 2011. 中国四川凤仙花属(凤仙花科)一新种——凉山凤仙花. 广西植物. 31(4): 433-435.
928. 罗强, 刘建林, 蔡光泽. 2011. 中国栘(木衣)属(Docynia Dcne.)一新种——长爪栘(木衣). 植物研究. 31(4): 389-391.
929. 罗强. 2010. 四川猕猴桃属(猕猴桃科)一新变种——凉山猕猴桃. 西昌学院学报(自然科学版). 24(2): 1-2.
930. 罗强. 2011. 木荷属(山茶科)一新变种——扁果银木荷. 热带亚热带植物学报. 19(3): 228-229.
931. 罗献瑞. 1999. 中国植物志 71(1)卷(册). 科学出版社, 北京.
932. 罗仲春,罗毅波编著. 2008. 新宁植物. 中国林业出版社, 北京.
933. 马丹丹, 金水虎. 2010. 黄山紫荆一新变型——白花黄山紫荆(英文). 浙江林学院学报. 27(2): 277.
934. 马德滋,刘惠兰,胡福秀. 2007. 宁夏植物志(第二版) (上)卷. 宁夏人民出版社, 银川.
935. 马德滋,刘惠兰,胡福秀. 2007. 宁夏植物志(第二版) (下)卷. 宁夏人民出版社, 银川.
936. 马德滋,刘惠兰. 1986. 宁夏植物志 1卷. 宁夏人民出版社, 银川.
937. 马德滋,刘惠兰. 1990. 宁夏植物志 2卷. 宁夏人民出版社, 银川.
938. 马国青,梁兵宽,马永珍,张玉钧. 2009. 青海大通北川河源区自然保护区生物多样性研究. 北京出版社, 北京.
939. 马金双. 1997. 中国植物志 44(3)卷(册). 科学出版社, 北京.
940. 马立华, 郁永英, 谭振平, 谷淑芬. 2010. 锦带花属2个新品种. 植物研究. 30(5): 629-631.
941. 马丽莎, 易同培, 史军义, 李本祥. 2010. 越香竹——香竹属一新种. 植物研究. 30(4): 390-393.
942. 马琴, 曹瑞. 2011. 内蒙古肉苁蓉属肉苁蓉一新变种——扇形肉苁蓉. 西北植物学报. 31(3): 639-641.
943. 马声远,马建伦,王霞,刘正宇,张毓红,罗伟洪,唐洪通,黄军,谢章桂,谭杨梅. 1998. 金佛山自然保护区科学考察集. 重庆南川市林业局,重庆市金佛山自然保护区管理处.
944. 马万里. 2007. 辽宁白狼山自然保护区综合科学考察报告.
945. 马养俊. 1985. 大岗山植物名录. 内部资料未正式出版.
946. 马玉心,曲秀春,崔大练. 2001. 黑龙江省东部异燕麦属一新种. 植物研究. 21(4):506-507.
947. 马玉心. 2002. 黑龙江省东部早熟禾属(Poa)一新种. 植物研究. 22(4):387-388.
948. 马毓泉主编. 1989. 内蒙古植物志(第二版) 3卷. 内蒙古人民出版社, 呼和浩特.
949. 马毓泉主编. 1990. 内蒙古植物志(第二版) 2卷. 内蒙古人民出版社, 呼和浩特.
950. 马毓泉主编. 1993. 内蒙古植物志(第二版) 4卷. 内蒙古人民出版社, 呼和浩特.
951. 马毓泉主编. 1994. 内蒙古植物志(第二版) 5卷. 内蒙古人民出版社, 呼和浩特.
952. 马毓泉主编. 1998. 内蒙古植物志(第二版) 1卷. 内蒙古人民出版社, 呼和浩特.
953. 马占忠,郭海荣编著. 2009. 古浪树种资源. 青海人民出版社, 西宁.
954. 满秀玲,范金凤. 2008. 小兴安岭北部沼泽湿地化学过程. 东北林业大学出版社, 哈尔滨.
955. 毛祖美,冯惠兰,刘国钧,沈观冕. 1981. 新疆药用植物志 2册. 新疆人民出版社, 乌鲁木齐.
956. 毛祖美. 1994. 新疆植物志 2(1)卷(册). 新疆科技卫生出版社, 乌鲁木齐.
957. 毛祖美. 1995. 新疆植物志 2(2)卷(册). 新疆科技卫生出版社, 乌鲁木齐.
958. 梅志奋. 1999. 北京常见树木. 中国林业出版社, 北京.
959. 米吉提•胡达拜尔地,潘晓玲. 2004. 新疆植物志 4卷. 新疆科技卫生出版社, 乌鲁木齐.
960. 闵天禄主编. 2000. 世界山茶属的研究. 云南科学技术出版社, 昆明.
961. 牟凤娟, 马士祝, 李双智, 张奠湘. 2011. 米兰山小橘(芸香科)的分类订正(英文). 广西植物. 31(6): 711-713.
962. 穆立蔷,韩志坚. 2004. 中国保护植物分类与识别. 东北林业大学出版社, 哈尔滨.
963. 穆萨. 2003. 广东佛冈观音山植物区系、植被及森林效益评价. 华南农业大学(博士论文).
964. 内蒙古自治区林业勘察设计院. 2007. 内蒙古巴音杭盖自然保护区综合科学考察报告. 内蒙古自治区林业勘察设计院.
965. 倪红伟,李君,赵晓民,周瑞昌,吴海一,赵永生,周志强,杨锡臣,郝安林,高玉慧,朱宝光,崔涛. 1999. 洪河自然保护区生物多样性. 黑龙江科学技术出版社, 哈尔滨.
966. 倪红伟主编. 2001. 挠力河自然保护区综合考察报告. 黑龙江省科学院生物多样性重点实验室,黑龙江省农垦勘测设计研究院,黑龙江省农垦总局环境保护局.
967. 倪志诚,程树志. 1992. 西藏南迦巴瓦峰地区维管束植物区系. 北京科学技术出版社, 北京.
968. 倪志诚. 1990. 西藏经济植物. 北京科学出版社, 北京.
969. 宁朝枢,蒋瑞海. 2002. 河北大海陀自然保护区科学考察集. 中国林业出版社, 北京.
970. 宁世江,李锋,何成新. 2009. 生物多样性关键地区－广西元宝山科学考察研究. 广西科学技术出版社, 南宁.
971. 宁小清, 刘寿养. 2010. 广西野牡丹科一新种,大明山异药花. 广西植物. 30(6): 825-826.
972. 牛春山主编. 1990. 陕西树木志. 中国林业出版社, 北京.
973. 牛平山,宋雪琳,李凯清,法蕾,杨妍妍,温学友,戴培青著. 2007. 泥河湾自然保护区资源与环境保护. 地震出版社, 北京.
974. 欧阳峰,王磊. 2009. 玛曲湿地保护管理. 甘肃人民出版社, 兰州.
975. 潘金贵,韦直主编. 1996. 浙江省九龙山自然保护区自然资源研究. 中国林业出版社, 北京.
976. 潘锦堂. 1992. 中国植物志 34(2)卷(册). 科学出版社, 北京.
977. 潘先海. 2006. 海南省常见有毒野果彩色图鉴. 海南出版社, 海口.
978. 潘学清. 2009. 呼伦贝尔市药用植物. 中国农业出版社, 北京.
979. 潘学清主编. 1992. 中国呼伦贝尔草地. 吉林科学技术出版社, 长春.
980. 庞雄飞,张金泉. 2008 广东石门台自然保护区综合科学考察报告. 英德市人民政府.
981. 庞雄飞主编. 2003. 广东南岭国家级自然保护区生物多样性研究. 广东科学技术出版社, 广州.
982. 裴鉴,陈守良. 1982. 中国植物志 65(1)卷(册). 科学出版社, 北京.
983. 裴鉴,丁志遵. 1985. 中国植物志 16(1)卷(册). 科学出版社, 北京.
984. 裴鑑,單人骅主编. 1958. 江苏南部种子植物检索表. 科学出版社, 北京.
985. 裴盛基,陈三阳. 1991. 中国植物志 13(1)卷(册). 科学出版社, 北京.
986. 彭国栋总编. 1983. 南投县植物资源. 台湾省特有生物研究保育中心(舜程彩色印刷公司), 台中市.
987. 彭国栋总编. 1997. 彰化县植物资源. 台湾省政府农林厅台湾省特有生物研究保育中心.
988. 彭华. 1998. 滇中南无量山种子植物. 云南科学技术出版社, 昆明.
989. 彭华. 2006. 云南植物志 10卷. 科学出版社, 北京.
990. 彭明春,王崇云,党承林主编. 2006. 云南药山自然保护区生物多样性及保护研究. 科学出版社, 北京.
991. 彭少麟,陈万成主编. 2003. 广东珍稀濒危植物. 科学出版社, 北京.
992. 彭少麟,廖文波,王英永,贾凤龙,凡强,沈如江,李贞,吴金火,陈晖. 2008. 中国三清山生物多样性综合科学考察. 科学出版社, 北京.
993. 彭余开主编. 2009. 五邑中草药图谱. 军事医学科学出版社.
994. 彭镇华主编. 2005. 中国长江三峡植物大全 (上)卷. 科学出版社, 北京.
995. 彭镇华主编. 2005. 中国长江三峡植物大全 (下)卷. 科学出版社, 北京.
996. 祁承经,林亲众. 2001. 湖南树木志. 湖南科学技术出版社, 长沙.
997. 祁承经. 1987. 湖南植物名录. 湖南科学技术出版社, 长沙.
998. 齐伟,郭怀林,孛随文. 2002. 麦积山林区草本植物鉴定手册. 甘肃人民美术出版社, 兰州.
999. 钱宏. 1990. 长白山高山冻原维管植物区系地理. 地理科学. 10(4):316-325.
1000. 钱士心,刘民壮,顺庆生编著. 1959. 上海的野生植物. 上海科学技术出版社, 上海.
1001. 钱啸虎,蒋木青,陈仁钧,卢心固,李书春,刘秀梅,吴诗华,吴诚和,吴泽民. 1986. 安徽植物志 1卷. 安徽科学技术出版社, 合肥.
1002. 钱啸虎,邵建章,张定成,王学文,李书春,吴诗华,沈祖安,吴学良,苏宋旺,陈仁钧,蒋木青,刘登义. 1992. 安徽植物志 5卷. 安徽科学技术出版社, 合肥.
1003. 钱啸虎,吴泽民,吴诚和,吴诗华,李秾,刘秀梅,薛兆文,李书春,蒙仁宪,武祖发,王德群,黄世华,孙乐知,张定成,吴学良,郭新弧,邵建章,王学文. 1987. 安徽植物志 2卷. 中国展望出版社, 北京.
1004. 钱义咏. 2001. 云南苞叶兰属一新种. 植物分类学报. 39(3):278-279.
1005. 钱义咏. 2001. 云南灯心草属一新种. 植物研究. 21(1):3-4.
1006. 钱义咏. 2001. 云南莎草属一新种. 植物研究. 21(3):335-336.
1007. 钱义咏. 2002. 云南囊颖草属一新种. 植物研究. 22(2):257-258.
1008. 钱义咏. 2003. 云南长柄山蚂蝗属一新种. 植物研究. 23(2):131-132.
1009. 钱子刚,李安华主编. 2008. 高黎贡山药用植物名录. 科学出版社, 北京.
1010. 秦仁昌,邢公侠. 1990. 中国植物志 3(1)卷(册). 科学出版社, 北京.
1011. 秦忠时主编. 2004. 东北草本植物志 10卷. 科学出版社, 北京.
1012. 青海省林业局编著. 1990. 青海省孟达自然保护区. 青海人民出版社, 西宁.
1013. 丘华兴,林有润. 1988. 中国植物志 24卷. 科学出版社, 北京.
1014. 丘华兴. 1996. 中国植物志 44(2)卷(册). 科学出版社, 北京.
1015. 裘宝林主编. 1993. 浙江植物志 4卷. 浙江科学技术出版社, 杭州.
1016. 曲秀春,于爽编著. 2007. 黑龙江省东南部维管植物实习指导. 东北林业大学出版社, 哈尔滨.
1017. 饶军,李江主编. 2009. 抚州植物资源. 清华大学出版社, 北京.
1018. 热衣木•马木提, 阿地里江•阿不都拉, 阿不都拉•阿巴斯. 2010. 中国新记录属——裂片茶渍衣属及一个中国新记录种(英文). 武汉植物学研究. 28(3): 362-364.
1019. 任毅,刘明时,田联会,田先华,李智军. 2006. 太白山自然保护区生物多样性研究与管理. 中国林业出版社, 北京.
1020. 任毅,温站强,李刚. 2008. 陕西米仓山自然保护区综合科学考察报告. 科学出版社, 北京.
1021. 任玉平,冯海记,朱进忠,安沙舟,李长和,陈建录,朱明保,陈爱莲. 1988. 新疆昌吉回族自治州草场饲料资源调查报告. 昌吉州草场资源调查队, 中科院植物所图书馆内部资料.
1022. 茹文明,张金屯,张桂萍,张建国,金山,铁军. 2005. 晋东南山地种子植物区系多样性研究. 长治学院学报. 22(2):4-8.
1023. 赛胜宝,李德新主编. 1995. 荒漠草原生态系统研究. 内蒙古人民出版社, 呼和浩特.
1024. 山东经济植物编写组. 1978. 山东经济植物. 山东人民出版社, 济南.
1025. 山东农学院植物学教研编组. 1964. 泰山植物名录. 山东农学院科学研究部, 济南.
1026. 山东省林业勘察设计院. 2000. 山东荣成大天鹅自然保护区. 山东省林业勘察设计院.
1027. 山东树木志编写组. 1984. 山东树木志. 山东科学技术出版社, 济南.
1028. 陕西龙池自然保护区综合科学考察队. 2007. 陕西龙池自然保护区综合科学考察报告.
1029. 陕西省林业厅编. 1989. 太白山自然保护区综合考察论文集. 陕西师范大学出版社, 西安.
1030. 上官铁梁,马子清,谢树莲著. 1998. 山西省珍稀濒危保护植物. 中国科学技术出版社, 北京.
1031. 佘坚强,曹九明,刘守礼,杨茂森,申如明,赵国伟. 1987. 甘肃蜜源植物志. 甘肃科学技术出版社, 兰州.
1032. 沈茂才主编. 2008. 秦岭植物园科学考察报告. 陕西科学技术出版社, 西安.
1033. 时华民,张铭哲,陈介,裴元蓉,丁宝章,萇哲新. 1963. 河南经济植物志. 河南人民出版社, 郑州.
1034. 时裕华主编. 1992. 包头生物资源. 内蒙古人民出版社, 呼和浩特.
1035. 税玉民,陈文红. 2004. 中国秋海棠属等翅组植物订正. 云南植物研究. 36(5):482-486.
1036. 税玉民,陈文红. 2006. 中国喀斯特地区种子植物 1(滇东南部)册. 科学出版社, 北京.
1037. 税玉民,黄素华. 1999. 滇东南大围山地区远志科一新种. 云南植物研究. 21(1):11-23.
1038. 税玉民. 1996. 文山县老君山维管植物. 文山县林业局云南省林业学校.
1039. 税玉民. 2002. 滇东南黄连山秋海棠属一新种. 云南植物研究. 24(3):307-308.
1040. 税玉民. 2002. 云南东南部秋海棠属侧膜组一新种. 植物分类学报. 40(4):374-376.
1041. 税玉民主编. 2003. 滇东南红河地区种子植物. 云南科学技术出版社, 昆明.
1042. 四川省稻城县人民政府. 1999. 四川亚丁自然保护区总体规则. 四川省稻城县人民政府.
1043. 四川省林业科学研究院. 2006. 四川若尔盖湿地国家级自然保护区综合科学考察报告.
1044. 四川省林业厅. 2001. 四川白水河自然保护区综合考察报告. 四川省林业厅.
1045. 四川省水产局,四川省渔政船检港监管理处. 1999. 长江合江－雷波段自然保护区资料汇编.
1046. 四川省野生动物资源调查保护管理站,四川省林业科学研究院. 1999. 四川王朗自然保护区综合科学考察报告. 内部资料未正式出版.
1047. 宋朝枢,瞿文元. 1996. 董寨鸟类自然保护区科学考察集. 中国林业出版社, 北京.
1048. 宋朝枢,瞿文元主编. 1996. 太行山猕猴自然保护区科学考察集. 中国林业出版社, 北京.
1049. 宋朝枢,刘胜祥主编. 1999. 湖北后河自然保护区科学考察集. 中国林业出版社, 北京.
1050. 宋朝枢,王呈荣主编. 2002. 甘肃莲花山自然保护区科学考察集. 中国林业出版社, 北京.
1051. 宋朝枢,王有德. 1999. 宁夏白芨滩自然保护区科学考察集. 中国林业出版社, 北京.
1052. 宋朝枢,郑建旭主编. 2001. 河北小五台山自然保护区综合科学考察报告. 河北小五台山自然保护区管理处.
1053. 宋朝枢. 1994. 宝天曼自然保护区科学考察集. 中国林业出版社, 北京.
1054. 宋朝枢. 1994. 伏牛山自然保护区科学考察集. 中国林业出版社, 北京.
1055. 宋朝枢. 1994. 鸡公山自然保护区科学考察集. 中国林业出版社, 北京.
1056. 宋朝枢. 1997. 浙江清凉峰自然保护区科学考察集. 中国林业出版社, 北京.
1057. 宋朝枢主编. 1991. 山西树木图志 1册. 科学出版社, 北京.
1058. 宋立鑫主编. 2000. 西藏察隅慈巴沟国家级自然保护区自然资源综合科学考察报告. 西藏自治区林业勘察设计研究院.
1059. 宋立鑫主编. 2000. 西藏芒康滇金丝猴国家级自然保护区自然资源综合科学考察报告. 西藏自治区林业勘察设计研究院.
1060. 宋柱秋, 许东先, 李世晋. 2011. 红果黄檀一新异名. 热带亚热带植物学报. 19(4): 320-322.
1061. 宋滋圃,秦自生. 2005. 四川植物志 16卷. 四川民族出版社, 成都.
1062. 苏鸿杰. 1977. 台湾国有林自然保护区植群生态之调查研究阿里山1叶兰保护区植群生态之研究.
1063. 苏鸿雁,杨晓霞. 2000. 云南寸金草新变型. 植物研究. 20(3):253
1064. 孙必兴,李德铢,薛纪如. 2003. 云南植物志 9卷. 科学出版社, 北京.
1065. 孙广华,王升忠,张雅斌,张德隆,杜晓燕主编. 2001. 吉林鸭绿江上游自然保护区科学考察报告. 吉林省水产科学研究所,吉林农业大学,东北师范大学,吉林省水利厅,农业部.
1066. 孙航,周浙昆. 2000. 雅鲁藏布江大峡弯河谷地区种子植物. 云南科学技术出版社, 昆明.
1067. 孙立元,任宪威. 1997. 河北树木志. 中国林业出版社, 北京.
1068. 孙祥钟. 1992. 中国植物志 8卷. 科学出版社, 北京.
1069. 孙祥钟. 1992. 中国植物志 8卷. 科学出版社, 北京.
1070. 孙悦华,方昀. 2008. 莲花山自然生态. 辽宁科学技术出版社, 沈阳.
1071. 孙治宇,刘少英. 2005. 四川长沙贡玛自然保护区综合科学考察报告. 四川省林业科学研究院.
1072. 台湾省林业试验所编辑. 1982. 台湾省林业试验所台北植物园植物名录. 台湾省政府印刷厂.
1073. 泰世民. 2001. 吉林天佛指山自然保护区综合考察报告. 吉林省林业勘察设计研究院.
1074. 覃海宁,方鼎主编. 2003. 广西那坡县种子植物名录. 中国科学技术出版社, 北京.
1075. 覃海宁,刘演主编. 2010. 广西植物名录. 科学出版社, 北京.
1076. 覃勇荣. 2000. 十万大山植物区系的基本特征. 河池师范高等专科学校学报. 20(2):9-14.
1077. 谭伟福. 2005. 广西岑王老山自然保护区生物多样性保护研究. 中国环境科学出版社, 北京.
1078. 谭伟福. 2005. 广西十万大山自然保护区生物多样性及其保护体系. 中国环境科学出版社, 北京.
1079. 谭仲明. 1999. 四川植物志 14卷. 四川民族出版社, 成都.
1080. 汤慧敏, 邓云飞. 2011. 中国爵床科一新记录种——毛萼爵床(英文). 热带亚热带植物学报. 19(4): 323-326.
1081. 唐昌林. 1996. 中国植物志 26卷. 科学出版社, 北京.
1082. 唐进,汪发缵. 1961. 中国植物志 11卷. 科学出版社, 北京.
1083. 唐进,汪发缵. 1980. 中国植物志 14卷. 科学出版社, 北京.
1084. 唐启孟. 1986. 四川省甘孜藏族自治州农业资源调查及区划草地资源. 甘孜州农业区划办公室,草地站编印.
1085. 唐小平,何承仁,宋朝枢主编. 2001. 甘肃民勤连古城自然保护区科学考察集. 中国林业出版社, 北京.
1086. 唐小平,王志臣,张明祥. 2006. 河南丹江湿地自然保护区生物多样性. 北京出版社, 北京.
1087. 田传铭主编. 2002. 大连树木图鉴. 大连出版社, 大连.
1088. 田怀珍, 李宏庆, 杨智凯. 2010. 天麻属——广东省兰科植物一新记录属(英文). 热带亚热带植物学报. 18(5): 488-490.
1089. 田家怡,申保忠,高奎江,孟向东,潘怀剑,贾建华,傅荣恕,刘恩家. 2002. 鹤伴山国家森林公园生物多样性与生态旅游建设. 山东省地图出版社, 济南.
1090. 田旗, 葛斌杰, 王正伟. 2011. 四川省杜鹃花属植物地理分布新记录. 西北植物学报. 31(1): 192-194.
1091. 田随味,田德雨,宋伯为,张军,张文恒,张永亮,赵天良,赵晋龙,赵益善. 山西省蟒河自然保护区综合考察报告及总体规则设计. 山西省林业勘测设计院,中条山森林经营局勘测设计队.
1092. 田志慧, 陈晓双, 达良俊. 2010. 上海两种新记录植物. 西北植物学报. 30(3): 624-625.
1093. 涂英芳,杨野,衣俊鹏. 1993. 长白山野生观赏植物. 中国林业出版社, 北京.
1094. 屠玉麟. 1991. 贵州特有植物初步研究(二). 贵州林业科技. 19(4):71-78.
1095. 屠玉麟. 1991. 贵州特有植物初步研究(一). 贵州林业科技. 19(3):68-80.
1096. 屠玉麟. 1992. 贵州特有植物初步研究(三). 贵州林业科技. 20(2):69-80.
1097. 庹德政,刘胜祥. 2006. 湖北湿地. 湖北科学技术出版社, 武汉.
1098. 宛涛,卫智军著. 1999. 内蒙古草地现代植物花粉形态. 中国农业出版社, 北京.
1099. 宛涛,燕玲,李红,阿拉腾其其格. 2003. 贺兰山特有植物花粉形态特征研究. 中国草地. 25(1):5-8.
1100. 万泉,肖祥希,林瑞荣主编. 2009. 福建乡土油料植物. 中国林业出版社, 北京.
1101. 万煜. 1986. 广西龙虎山自然保护区维管植物名录. 龙虎山自然保护区管理站.
1102. 汪发缵,唐进. 1978. 中国植物志 15卷. 科学出版社, 北京.
1103. 汪庆, 邓懋彬, 杨如同, 姚淦. 2011. 江苏省维管植物分布新记录(四). 植物资源与环境学报. 19(4): 90-91.
1104. 汪书丽, 兰小中, 罗建. 2011. 西藏独一味属一新变型——白花独一味. 西北植物学报. 31(6): 1266-1267.
1105. 汪松,解炎. 2004. 中国物种红色名录 1卷. 高等教育出版社, 北京.
1106. 汪万森,卓卫华,田金萍,阴三军执行主编. 2001. 河南黄河湿地自然保护区科学考察集. 中国环境科学出版社, 北京.
1107. 汪正祥,朱兆泉,王立志,雷耘,蒲云海,刘兴虎. 2008. 湖北漳河源自然保护区生物多样性及其保护研究. 科学出版社, 北京.
1108. 王德群. 1999. 安徽省特有植物的分类、分布和药用类群. 中国中药杂志. 24(8):451-454.
1109. 王冬米,陈征海主编. 2010. 台州乡土树种识别与应用. 浙江科学技术出版社, 杭州.
1110. 王二林主编. 2007. 天津滨海盐生植物. 中国林业出版社, 北京.
1111. 王发国,陈坚,邢福武,曾庆文,陈红锋. 2010. 东莞珍稀植物. 华中科技大学出版社, 武汉.
1112. 王国宏,冯自诚. 1996. 甘肃木本植物区系中特有现象分析. 甘肃农业大学学报. 31(3):282-287.
1113. 王国平. 2007. 西溪的植物. 杭州出版社, 北京.
1114. 王荷生. 1997. 华北植物区系地理. 科学出版社, 北京.
1115. 王菏生. 1992. 植物区系地理. 科学出版社, 北京.
1116. 王红,张长芹,李德铢,薛润光,杨亲二著. 2007. 丽江高山植物园种子植物名录. 云南科学技术出版社, 昆明.
1117. 王洪,朱华. 1999. 云南厚壳桂属一新种. 广西植物. 19(3):197-198.
1118. 王华青,吴振海. 2006. 陕西黄河湿地自然保护区综合科学考察与研究. 陕西科学技术出版社, 西安.
1119. 王晖主编. 1991. 五常树木志. 黑龙江科学技术出版社, 哈尔滨.
1120. 王继和. 2008. 库姆塔格沙漠综合科学考察. 甘肃科学技术出版社, 兰州.
1121. 王金潮,李天珍,周建成,杨建明,张进宝. 2006. 太原地区树木物种资源. 山西科学技术出版社, 太原.
1122. 王进, 罗光宏, 陈叶, 张勇, 祖廷勋. 2011. 锁阳寄主植物的一个国内新记录——多裂骆驼篷. 中国中药杂志. 26(23): 3244-3246.
1123. 王景权,李殿明,袁晓影. 2002. 柳属一新变种. 植物研究. 22(1):9
1124. 王景祥主编. 1992. 浙江植物志 2卷. 浙江科学技术出版社, 杭州.
1125. 王九中主编. 2008. 百花山植物 1. 气象出版社, 北京.
1126. 王军, 夏念和. 2011. 缅甸杜茎山属(杜茎山科)一新变种(英文).热带亚热带植物学报. 19(3): 225-227.
1127. 王俊. 2007. 六盘山药用植物原色图谱 (上)册. 宁夏人民出版社, 银川.
1128. 王黎元,苏建英编著. 2002. 包头高等植物检索表. 包头师范学院生命科学系.
1129. 王良民, 张玉钧. 2011. 论辽东栎(壳斗科)的分类地位及命名. 植物科学学报. 29(6): 749-754.
1130. 王良民著. 2010. 山西太宽河自然保护区植物多样性研究. 中国农业科学技术出版社, 北京.
1131. 王玛丽,邢连喜,张国昌主编. 2004. 陕西化龙山自然保护区综合科学考察报告. 西安地图出版社发行, 西安.
1132. 王民柱,唐臻编. 1997. 汉中植物名录. 陕西科学技术出版社, 西安.
1133. 王宁珠,张树藩,黄仁煌,马芳莲. 1983. 中国水生维管束植物图谱. 湖北人民出版社, 武汉.
1134. 王培善,王筱英. 2001. 贵州蕨类植物志. 贵州科学技术出版社, 贵阳.
1135. 王培善. 1992. 梵净山蕨类植物. 贵州科学技术出版社, 贵阳.
1136. 王鹏,邱英杰主编. 2002. 辽宁仙人洞国家级自然保护区科学考察集. 中国林业出版社, 北京.
1137. 王青锋,葛继稳. 2002. 湖北九宫山自然保护区生物多样性及其保护. 中国林业出版社, 北京.
1138. 王庆瑞. 1991. 中国植物志 51卷. 科学出版社, 北京.
1139. 王庆锁. 2004. 河北北部、内蒙古东部森林草原交错带植被和生物多样性研究. 气象出版社, 北京.
1140. 王绍先. 2007. 长白山保护开发区生物资源. 辽宁科学技术出版社, 沈阳.
1141. 王诗云,赵子恩,彭铺松,蒋祖德. 1995. 华中珍稀濒危植物及其保存 1册. 科学出版社, 北京.
1142. 王遂义. 1994. 河南树木志. 河南科学技术出版社, 郑州.
1143. 王铁娟,赵一之. 2000. 绣线菊属二新种. 植物研究. 20(4):361-364.
1144. 王万鹏,俞诗源,钟芳. 2010. 兰州市南北两山植物动物资源. 甘肃科学技术出版社, 兰州.
1145. 王维国主编. 2008. 克什克腾旗植物名录. 内蒙古科学技术出版社, 赤峰.
1146. 王文采, 李良千. 2011. 甘南铁线莲,甘肃毛茛科一新种. 广西植物. 31(3): 285-287.
1147. 王文采,陈家瑞. 1995. 中国植物志 23(2)卷(册). 科学出版社, 北京.
1148. 王文采. 1979. 中国植物志 27卷. 科学出版社, 北京.
1149. 王文采. 1980. 中国植物志 28卷. 科学出版社, 北京.
1150. 王文采. 1980. 中国植物志 2卷. 科学出版社, 北京.
1151. 王文采. 1990. 中国植物志 69卷. 科学出版社, 北京.
1152. 王文采. 2010. 葡萄属一新名和一新种. 广西植物. 30(3): 287-289.
1153. 王文采. 2010. 荨麻科赤车属和楼梯草属新分类群. 广西植物. 30(1): 1-12.
1154. 王文采. 2010. 紫草科新分类群. 广西植物. 30(4): 429-439.
1155. 王文采. 2011. 云南东南部楼梯草属二新种. 广西植物. 31(2): 143-147.
1156. 王文采. 2011. 云南高黎贡山荨麻科楼梯草属六新种. 植物分类与资源学报. 33(2): 145-156.
1157. 王文采. 2013. 中国荨麻科楼梯草属植物. 青岛出版社, 青岛.
1158. 王文采主编. 1995. 武陵山地区维管植物检索表. 科学出版社, 北京.
1159. 王喜贵,吴埃平. 1999. 山西黑茶山林区植物志. 山西科学技术出版社, 太原.
1160. 王香亭主编. 1996. 甘肃兴隆山国家级自然保护区资源本底调查研究. 甘肃民族出版社, 兰州.
1161. 王小平,张志翔,甘敬,赵良成,林秦文,秦永胜,余新晓,张钢民,M.克拉索夫斯基. 2008. 北京森林植物图谱. 科学出版社, 北京.
1162. 王毅, 王燕. 2010. 中国兰科玉凤兰属一新记录种——奇花玉凤兰(英文). 武汉植物学研究. 28(6): 696-697.
1163. 王英伟,刘全儒,张明理. 2003. 紫堇属(荷包牡丹科)一新种. 植物研究. 23(4):385-387.
1164. 王勇,李振宇,吴金清,黄宏文. 2004. 中国车前属(车前科)一新组合——丰都车前. 植物分类学报. 42(6):557-560.
1165. 王勇,吴金清,陶勇,李作洲,黄宏文. 2003. 三峡库区消涨带特有植物疏花水柏枝(*Myricaria laxiflora*)的自然分布及迁地保护研究. 武汉植物学研究. 21(5):415－422.
1166. 王羽梅. 2008. 中国芳香植物 (上). 科学出版社, 北京.
1167. 王玉兵, 陈发菊, 梁宏伟, 何泽卫. 2010. 湖北省玄参科一新记录属——幌菊属. 西北植物学报. 30(7): 1497-1498.
1168. 王战,方振富. 1984. 中国植物志 20(2)卷(册). 科学出版社, 北京.
1169. 王郑敏. 2002. 河北衡水湖自然保护区科学考察报告. 河北衡水湖自然保护区管理处.
1170. 王枝荣主编. 1990. 中国农田杂草原色图谱. 农业出版社, 北京.
1171. 王志臣,马国青,王九中. 2009. 北京百花山国家级自然保护区生物多样性研究. 北京出版社, 北京.
1172. 王仲朗,李恒. 1999. 高黎贡山斑龙芋(天南星科). 云南植物研究. 21(11):61-64.
1173. 王祝年,肖邦森. 2009. 海南药用植物名录. 中国农业出版社, 北京.
1174. 王作宾,傅坤俊,郭本兆,唐昌林. 1959. 西北野生有用植物手册 3册. 陕西人民出版社, 西安.
1175. 韦直,何业祺主编. 1993. 浙江植物志 3卷. 浙江科学技术出版社, 杭州.
1176. 韦直. 1994. 中国植物志 40卷. 科学出版社, 北京.
1177. 魏建和,朱平主编. 2007. 中国医学科学院药用植物研究所海南分所南药园植物名录. 中国农业出版社, 北京.
1178. 魏鲁明, 郎楷永, 余登利. 2010. 革叶石豆兰——中国兰科植物一新记录种. 广西植物. 30(4): 446-447.
1179. 魏鲁明, 玉屏, 余登利. 2010. 插天山羊耳蒜——中国大陆兰科植物新记录. 热带亚热带植物学报. 18(4): 399-400.
1180. 温远光,和太平,谭伟福. 2004. 广西热带和亚热带山地的植物多样性及群落特征. 气象出版社, 北京.
1181. 温战强,杨玉柱. 2007. 陕西桑园自然保护区科学考察报告. 陕西科学技术出版社, 西安.
1182. 文明. 1993. 长江中下游水域洲滩野生经济植物. 湖南科学技术出版社, 长沙.
1183. 翁国庆,李燕主编. 2006. 内蒙古哈腾套海国家级自然保护区生物多样性. 北京出版社, 北京.
1184. 乌仁格日乐. 2008. 达里诺尔自然保护区维管植物区系研究. 内蒙古农业大学(硕士论文).
1185. 邬文祥著. 1993. 无锡植物区系.
1186. 吴长申主编. 1999. 扎龙国家级自然保护区自然资源研究与管理. 东北林业大学出版社, 哈尔滨.
1187. 吴诚和,张定成,李秾,傅玉兰,郭新弧,钱啸虎,刘秀梅,李书春,吴诗华,沈显生,张小平,王学文,朱玉简,王树松,叶如欣,蒋木青,邵建章,钱宏. 1991. 安徽植物志 4卷. 中国展望出版社, 北京.
1188. 吴德邻,邢福武,李泽贤,叶华谷,陈炳辉,钟义. 1994. 海南及广东沿海岛屿植物名录. 科学出版社, 北京.
1189. 吴德邻. 1981. 中国植物志 16(2)卷(册). 科学出版社, 北京.
1190. 吴德邻. 1996. 红水河上游地区植物调查研究报告集. 科学出版社, 北京.
1191. 吴德邻主编. 1995. 广东植物志 3卷. 广东科学技术出版社, 广州.
1192. 吴德邻主编. 2000. 广东植物志 4卷. 广东科学技术出版社, 广州.
1193. 吴德邻主编. 2003. 广东植物志 5卷. 广东科学技术出版社, 广州.
1194. 吴德邻主编. 2005. 广东植物志 6卷. 广东科学技术出版社, 广州.
1195. 吴德邻主编. 2006. 广东植物志 7卷. 广东科学技术出版社, 广州.
1196. 吴德邻主编. 2007. 广东植物志 8卷. 广东科学技术出版社, 广州.
1197. 吴德邻主编. 2009. 广东植物志 9卷. 广东科学技术出版社, 广州.
1198. 吴德鄰主编. 2002. 香港植物名录. 香港特别行政区政府渔农自然护理属.
1199. 吴棣飞, 金孝锋. 2011. 浙江省兰科植物2新记录种. 西北植物学报. 31(9): 1891-1893.
1200. 吴国芳. 1997. 中国植物志 13(3)卷(册). 科学出版社, 北京.
1201. 吴虎山,潘英,王伟共. 2009. 呼伦贝尔市饲用植物. 中国农业出版社, 北京.
1202. 吴黄豪,纪伟涛. 2002. 江西鄱阳湖国家级自然保护区研究. 中国林业出版社, 北京.
1203. 吴剑雄主编. 2007. 鄂尔多斯植物志 (上)卷. 内蒙古人民出版社, 呼和浩特.
1204. 吴剑雄主编. 2007. 鄂尔多斯植物志 (下)卷. 内蒙古人民出版社, 呼和浩特.
1205. 吴金清,金义兴,赵子恩. 2008. 三峡库区大老岭植物多样性与保护. 中国水利水电出版社, 北京.
1206. 吴金清,赵子恩,金义兴著. 2009. 三峡库区珍稀濒危保护植物彩色图谱. 中国水利水电出版社, 北京.
1207. 吴隆生. 2010. 福州市种子植物地理分布新记录. 福建林业科技. 37(2): 98-99.
1208. 吴明江,赵宏,谢航,赵毓棠. 2000. 东北香蒲属一新种. 植物研究. 20(3):251-252.
1209. 吴容芬,黄淑美. 1987. 中国植物志 60(2)卷(册). 科学出版社, 北京.
1210. 吴望辉, 蒋日红, 农东新, 许为斌. 2010. 广西苦苣苔科一新记录属——报春苣苔属. 广西植物. 30(3): 290-291.
1211. 吴孝兵,顾长明,朱家龙. 2008. 安徽扬子鳄国家级自然保护区综合研究. 合肥工业大学出版社, 合肥.
1212. 吴修仁编著. 1997. 潮汕生物资源志略. 中山大学出版社发行, 广州.
1213. 吴玉虎,苟新京,梅丽娟. 1998. 青海植物名录. 青海人民出版社, 西宁.
1214. 吴玉虎,梅丽娟著. 2001. 黄河源区植物资源及其环境. 青海人民出版社, 西宁.
1215. 吴玉虎. 2003. 青海棘豆属一新变种. 植物研究. 23(4):391
1216. 吴玉虎. 2008. 青藏高原维管植物及其生态地理分布. 科学出版社, 北京.
1217. 吴跃峰,赵建成,程俊主编. 2006. 河北茅荆坝自然保护区科学考察与生物多样性研究. 科学出版社, 北京.
1218. 吴跃峰,赵建成,刘宝忠主编. 2007. 河北辽河源自然保护区科学考察与生物多样性研究. 科学出版社, 北京.
1219. 吴章文,陈就和,吴楚才主编. 2001. 广东象头山自然保护区科学考察集. 内部资料未正式出版.
1220. 吴兆洪. 1999. 中国植物志 4(2)卷(册). 科学出版社, 北京.
1221. 吴兆洪. 1999. 中国植物志 6(1)卷(册). 科学出版社, 北京.
1222. 吴振海, 田涛, 陈西, 杜诚. 2010. 秦岭兰科一新分布种——囊唇山兰. 西北植物学报. 30(9): 1917-1918.
1223. 吴征镒,李恒. 1979. 中国植物志 13(2)卷(册). 科学出版社, 北京.
1224. 吴征镒,李锡文. 1977. 中国植物志 65(2)卷(册). 科学出版社, 北京.
1225. 吴征镒,李锡文. 1977. 中国植物志 66卷. 科学出版社, 北京.
1226. 吴征镒,李锡文主编. 1983. 云南植物志 3卷. 科学出版社, 北京.
1227. 吴征镒. 1979. 中国植物志 64(1)卷(册). 科学出版社, 北京.
1228. 吴征镒. 1999. 中国植物志 32卷. 科学出版社, 北京.
1229. 吴征镒主编. 1977. 云南植物志 1卷. 科学出版社, 北京.
1230. 吴征镒主编. 1979. 云南植物志 2卷. 科学出版社, 北京.
1231. 吴征镒主编. 1980. 中国植被. 科学出版社, 北京.
1232. 吴征镒主编. 1983. 西藏植物志 1卷. 科学出版社, 北京.
1233. 吴征镒主编. 1984. 云南种子植物名录 (上)册. 云南人民出版社, 昆明.
1234. 吴征镒主编. 1985. 西藏植物志 2卷. 科学出版社, 北京.
1235. 吴征镒主编. 1985. 西藏植物志 4卷. 科学出版社, 北京.
1236. 吴征镒主编. 1986. 西藏植物志 3卷. 科学出版社, 北京.
1237. 吴征镒主编. 1986. 云南植物志 4卷. 科学出版社, 北京.
1238. 吴征镒主编. 1987. 西藏植物志 5卷. 科学出版社, 北京.
1239. 吴征镒主编. 1991. 云南植物志 5卷. 科学出版社, 北京.
1240. 吴征镒主编. 1995. 云南植物志 6卷. 科学出版社, 北京.
1241. 吴征镒主编. 1997. 云南植物志 7卷. 科学出版社, 北京.
1242. 伍光和,张可荣主编. 1997. 甘肃白水江国家级自然保护区综合科学考察报告. 甘肃科学技术出版社, 兰州.
1243. 伍煜廷. 1999. 四川植物志 13卷. 四川民族出版社, 成都.
1244. 武光南, 夏念和, 裴世山. 2010. 越南木兰科植物新记录(英文). 热带亚热带植物学报. 18(6): 661-664.
1245. 武光南, 夏念和. 2010. 越南木兰科植物新记录(英文). 热带亚热带植物学报. 18(1): 43-46.
1246. 武光南, 夏念和. 2011. 越南拟单性木兰属一新组合(英文). 热带亚热带植物学报.(4).
1247. 武光南, 夏念和. 2011. 越南植物Michelia tonkinensis(木兰科)的模式考订(英文). 热带亚热带植物学报. 19(6): 549-553.
1248. 武丕琼主编. 1989. 云南野生饲用植物. 云南科学技术出版社, 昆明.
1249. 武素功. 2000. 中国植物志 5(1)卷(册). 科学出版社, 北京.
1250. 西藏林业勘察设计研究所. 1999. 中国西藏羌塘国家级自然保护区总体规划.
1251. 西南林学院,云南省林业厅,屏边县大围山保护区管理所,河口县大围山保护区管理所. 1999. 大围山自然保护区. 内部资料未正式出版.
1252. 西双版纳州环境监测站. 1989. 西双版纳纳板河流域生物圈保护区综合考察研究. 内部资料未正式出版.
1253. 夏光成,张庆荣. 1994. 有毒中草药彩色图鉴. 天津科技翻译出版公司, 天津.
1254. 夏国华, 李根有. 2011. 高野山龙头草——中国唇形科植物新记录. 广西植物. 31(5): 581-583.
1255. 夏念和,邓云飞,叶国梁. 2006. 国产樟科-新种-香港油果樟. 热带亚热带植物学报. 14(1):75-77.
1256. 夏念和,韦发南,邓云飞. 2006. 香港樟科一新种－腺叶琼楠. 热带亚热带植物学报. 14(1):78-80.
1257. 向其柏, 唐开山, 杜多青. 2010. 湖南冬青属一新种. 植物研究. 30(6): 645-647.
1258. 肖培根主编. 2002. 新编中药志 1卷. 化学工业出版社, 北京.
1259. 肖培根主编. 2002. 新编中药志 2卷. 化学工业出版社, 北京.
1260. 肖培根主编. 2007. 新编中药志 5卷. 化学工业出版社, 北京.
1261. 肖文发,陈龙清,苏化龙,林英华,聂必红著. 2009. 重庆五里坡自然保护区生物多样性. 中国林业出版社, 北京.
1262. 肖文发,李建文,于长青,马娟,程瑞梅,刘少英,王金锡,葛继稳. 2000. 长江三峡库区陆生动植物生态. 西南师范大学出版社, 重庆.
1263. 谢碧霞,陈训主编. 2008. 中国木本淀粉植物. 科学出版社, 北京.
1264. 谢良生,雷江丽,庄雪影,谭一凡,曹华,郑明轩. 2007. 深圳植物名录. 中国林业出版社, 北京.
1265. 谢儒,柏梁真,朱玉奇. 2010. 中国甘肃野生观赏植物. 上海文化出版社, 上海.
1266. 谢双喜,李明晶,喻理飞. 2000. 贵州朱家山自然保护区科学考察集. 中国林业出版社, 北京.
1267. 谢双喜,喻理飞,周庆主编. 2006. 大沙河自然保护区本底资源. 贵州科学技术出版社, 贵阳.
1268. 谢寅堂,王玛丽,赵桂仿. 2007. 西安植物志. 陕西科学技术出版社, 西安.
1269. 谢宗强,陈伟烈. 1994. 三峡库区特有植物及三峡工程对其影响. 国土与自然资源研究. 4:61-65.
1270. 辛海静, Do, Van, HAI, 邓云飞. 2010. 爵床科蛇根叶属一新组合——越南蛇根叶(英文). 热带亚热带植物学报. 18(4): 397-398.
1271. 辛泽华,张子健,范喜梅主编. 2002. 焦作植物志. 西安地图出版社, 西安.
1272. 新疆林科院. 2000. 新疆甘家湖梭梭自然保护区综合考察报告总体规划. 新疆林科院,新疆甘家湖梭梭自然保护区乌苏管理站精河管理站.
1273. 新疆林业勘察设计院,新疆林业厅天山西部林业局. 1997. 新疆巩留雪岭云杉自然保护区综合考察报告. 新疆林业勘察设计院成品资料室.
1274. 新疆生物土壤沙漠研究所编. 1977. 新疆药用植物志 1册. 新疆人民出版社, 乌鲁木齐.
1275. 邢福武,陈坚,曾庆文,王发国,陈红锋主编. 2010. 东莞植物志. 华中科技大学出版社, 武汉.
1276. 邢福武,吴德邻. 1995. 海南岛特有植物的研究. 热带亚热带植物学报. 3(1):1-12.
1277. 邢福武,吴德邻主编. 1996. 南沙群岛及其邻近岛屿植物志. 海洋出版社, 北京.
1278. 邢福武,叶华谷. 2005. 澳门植物志 1卷, 澳门特别行政区民政总署园林绿化部,中国科学院华南植物园.
1279. 邢福武,叶华谷. 2006. 澳门植物志 2卷, 澳门特别行政区民政总署园林绿化部,中国科学院华南植物园.
1280. 邢福武,叶华谷. 2007. 澳门植物志 3卷, 澳门特别行政区民政总署园林绿化部,中国科学院华南植物园.
1281. 邢福武,余明恩,张永夏. 2003. 深圳植物物种多样性及其保育. 中国林业出版社, 北京.
1282. 邢福武,余明恩. 2000. 深圳野生植物. 中国林业出版社, 北京.
1283. 邢福武,曾庆文,谢左章. 2011. 广州野生植物. 华中科技大学出版社.
1284. 邢福武,周远松,龚友夫,张永夏. 2004. 深圳市七娘山郊野公园植物资源与保护. 中国林业出版社, 北京.
1285. 邢福武. 2005. 中国的珍稀植物. 湖南教育出版社, 长沙.
1286. 邢福武主编. 2004. 澳门植物名录. 澳门民政总署园林绿化部.
1287. 邢公侠. 1999. 中国植物志 4(1)卷(册). 科学出版社, 北京.
1288. 雄济华. 1990. 四川植物志 8卷. 四川民族出版社, 成都.
1289. 熊济华主编. 2005. 重庆缙云山植物志. 西南师范大学出版社, 重庆.
1290. 熊源新,杨传东著. 2009. 梵净山国家级自然保护区常见草本种子植物图鉴. 贵州科学技术出版社, 贵阳.
1291. 徐炳声. 1988. 中国植物志 72卷. 科学出版社, 北京.
1292. 徐炳声编著. 1959. 上海植物名录. 上海科学技术出版社, 上海.
1293. 徐炳声主编. 1999. 上海植物志 (上)册. 上海科学技术文献出版社, 上海.
1294. 徐炳声主编. 1999. 上海植物志 (下)册. 上海科学技术文献出版社, 上海.
1295. 徐光禹,刘国海,朱志祥. 大连城山头海滨地貌自然保护区综合考察报告.
1296. 徐宏发,赵云龙. 2005. 上海市崇明东滩鸟类自然保护区科学考察集. 中国林业出版社, 北京.
1297. 徐景先,赵良成,林秦文主编. 2009. 北京湿地植物. 北京科学技术出版社, 北京.
1298. 徐来富. 2006. 贵州野生木本花卉. 贵州科学技术出版社, 贵阳.
1299. 徐朗然,黄成就. 1998. 中国植物志 43(1)卷(册). 科学出版社, 北京.
1300. 徐荣章. 1989. 天目山木本植物图鉴. 中国林业出版社, 北京.
1301. 徐万林. 1983. 中国蜜源植物. 黑龙江科学技术出版社, 哈尔滨.
1302. 徐文铎,刘广田主编. 1998. 内蒙古白音敖包自然保护区沙地云杉林生态系统研究. 中国林业出版社, 北京.
1303. 徐燕千主编. 1993. 车八岭国家级自然保护区调查研究论文集. 广东科学技术出版社, 广州.
1304. 徐养鹏,王克制,于兆英主编. 1993. 中国滩羊区植物志 2卷. 宁夏人民出版社, 银川.
1305. 徐养鹏,王克制主编. 1996. 中国滩羊区植物志 3卷. 宁夏人民出版社, 银川.
1306. 徐养鹏,王克制主编. 1996. 中国滩羊区植物志 4卷. 宁夏人民出版社, 银川.
1307. 徐永椿,姜汉桥,全复主编. 1987. 西双版纳自然保护区综合考察报告集. 云南科学技术出版社, 昆明.
1308. 徐永椿主编. 1988. 云南树木图志 (上)册. 云南科学技术出版社, 昆明.
1309. 徐永椿主编. 1990. 云南树木图志 (中)册. 云南科学技术出版社, 昆明.
1310. 徐永椿主编. 1991. 云南树木图志 (下)册. 云南科学技术出版社, 昆明.
1311. 徐志辉,李增耀. 2004. 中国云南红河州野生珍稀动植物. 云南科学技术出版社, 昆明.
1312. 徐志辉主编. 1998. 怒江自然保护区. 云南美术出版社, 昆明.
1313. 许建初. 2003. 云南绿春黄连山自然保护区. 云南科学技术出版社, 昆明.
1314. 许介眉. 1991. 四川植物志 7卷. 四川民族出版社, 成都.
1315. 许林书主编. 2000. 内蒙古图牧吉自然保护区科学考察报告. 内蒙古自治区图牧吉自然保护区,东北师范大学泥炭沼泽研究所.
1316. 许宁. 1990. 天津市蓟县八仙桌子自然保护区综合调查. 天津科学技术出版社, 天津.
1317. 许天全,吴金清. 2000. 三峡库区地方特有维管植物研究. 武汉植物学研究. 18(3):253-256.
1318. 许为斌, 黄俞淞, 叶晓霞, 刘演. 2010. 中国蜘蛛抱蛋属一新记录种——合瓣蜘蛛抱蛋. 广西植物. 30(5): 613-615.
1319. 许为斌, 刘演, 梁永延, 农东新. 2010. 陆氏细筒苣苔——中国广西苦苣苔科一新种(英文). 武汉植物学研究. 28(1): 7-9.
1320. 许为斌, 盘波, 梁永延, 朱运喜, 刘演. 2010. 广西植物区系新资料. 广西植物. 30(4): 448-450.
1321. 薛纪如主编. 1995. 高黎贡山国家自然保护区. 中国林业出版社, 北京.
1322. 闫丽春主编. 2009. 云南哀牢山种子植物. 云南科学技术出版社, 昆明.
1323. 严仲铠,李万林. 1997. 中国长白山药用植物彩色图志. 人民卫生出版社, 北京.
1324. 颜素珠. 1983. 中国水生高等植物图说. 科学出版社, 北京.
1325. 杨柏云, 孔令杰, 李波, 何纪力, 范志刚, 周广, 李恩香. 2011. 江西虾脊兰属一新变型——异钩距虾脊兰. 热带亚热带植物学报.(4).
1326. 杨昌煦,熊济华,钟世理,王海洋,李先源编著. 2009. 重庆维管植物检索表. 四川科学技术出版社, 成都.
1327. 杨昌友,崔乃然,安争夕,田允温. 1982. 新疆植物检索表 1册. 新疆人民出版社, 乌鲁木齐.
1328. 杨昌友,黄俊华. 2001. 新疆葱属—新变种. 植物研究. 21(2):186
1329. 杨昌友. 1992. 新疆植物志 1卷. 新疆科技卫生出版社, 乌鲁木齐.
1330. 杨成华, 陈景艳, 戴晓勇. 2011. 贵州报春花属植物的新分布. 贵州科学. 29(1): 32-33.
1331. 杨春澍,孙建宁,黄建海主编. 2006. 细辛属和八角属中药研究与应用. 人民卫生出版社, 北京.
1332. 杨道德,胡慧建主编. 2009. 武汉涨渡湖湿地生物多样性研究和保护. 广东科学技术出版社, 广州.
1333. 杨逢春,梁淑云. 2009. 海南蕨类植物. 中国林业出版社, 北京.
1334. 杨虎彪, 王清隆, 李晓霞, 刘国道. 2011. 海南莎草科一新记录属——针蔺属. 西北植物学报. 31(5): 1055-1056.
1335. 杨俊良,杨光辉. 1988. 四川植物志 5(2)卷(册). 四川科学技术出版社, 成都.
1336. 杨林,张淑萍主编. 2006. 北京陆生生物学野外实习指导. 中央民族大学出版社, 北京.
1337. 杨平厚,郎楷永. 2006. 陕西鸟巢兰属一新种——太白山鸟巢兰. 植物分类学报. 44(1):86-88.
1338. 杨平厚,孙承骞. 2007. 陕西野生兰科植物图鉴. 陕西科学技术出版社, 西安.
1339. 杨钦周主编. 1997. 四川树木分布. 贵州科学技术出版社, 贵阳.
1340. 杨野,刘军,魏成录等编. 1982. 长白山动植物名录. 吉林省长白山自然保护区研究所延边医学院印刷厂印刷(内部资料).
1341. 杨宇明,杜凡. 2004. 中国南滚河国家级自然保护区. 云南科学技术出版社, 昆明.
1342. 杨宇明,杜凡. 2006. 云南铜壁关自然保护区科学考察研究. 云南科学技术出版社, 昆明.
1343. 杨宇明,田昆,和世钧主编. 2008. 中国文山国家级自然保护区科学考察研究. 科学出版社, 北京.
1344. 杨远波,刘和义,林讚标编著. 2003. 台湾维管束植物简志 5卷, 中华民国行政院农业委员会出版发行.
1345. 杨远波,刘和义,吕胜由编著. 1999. 台湾维管束植物简志 2卷, 中华民国行政院农业委员会出版发行.
1346. 杨远波,刘和义,彭镜毅,施炳霖,吕胜由编著. 2002. 台湾维管束植物简志 4卷, 中华民国行政院农业委员会出版发行.
1347. 杨宗愈,周文能编著. 2005. 国立自然科学博物馆植物园植物名录. 国立自然科学博物馆.
1348. 姚德生,陈西仓,任继文. 2002. 甘肃杠柳属一新种. 植物研究. 22(3):259-260.
1349. 姚德生. 2004. 甘肃木本植物特有种类及其分布. 西北植物学报. 24(9):1714-1720.
1350. 姚纲, 张奠湘. 2010. 中国算盘子属植物(大戟科)2新记录种. 热带亚热带植物学报. 18(4): 394-396.
1351. 叶汉生,李成河主编. 1995. 南安生物资源. 鹭江出版社, 厦门.
1352. 叶华谷,陈邦余. 2005. 乐昌植物志 (上)册. 广东世界图书出版公司, 广州.
1353. 叶华谷,陈邦余. 2005. 乐昌植物志 (下)册. 广东世界图书出版公司, 广州.
1354. 叶华谷,彭少鳞. 2006. 广东植物多样性编目. 广东世界图书出版公司, 广州.
1355. 叶康, 刘启新, 邓懋彬, 褚晓芳. 2011. 江苏省维管植物分布新记录(三). 植物资源与环境学报. 19(3): 95-96.
1356. 叶康, 刘启新, 邓懋彬. 2010. 江苏省维管植物分布新记录(二). 植物资源与环境学报. 18(3): 89-90.
1357. 叶能干. 1989. 贵州植物志 7卷. 四川民族出版社, 贵阳.
1358. 叶晓霞, 黄俞淞, 农东新, 许为斌. 2010. 广西蝴蝶兰属(兰科)新资料. 广西植物. 30(6): 827-828.
1359. 叶永昌,苏志尧. 2008. 东莞森林生态建设模式与评价. 中国林业出版社, 北京.
1360. 叶永忠,瞿文元,黄远超主编. 2002. 连康山自然保护区科学考察集. 科学出版社, 北京.
1361. 叶永忠,汪万森,李合中. 2004. 河南小秦岭自然保护区科学考察集. 科学出版社, 北京.
1362. 叶永忠,吴顺卿. 1993. 崂山植物志. 中国科学技术出版社, 北京.
1363. 叶永忠,吴顺卿主编. 1993. 嵩山植物志. 中国科学技术出版社, 北京.
1364. 叶永忠,张赞平,葛得有. 2000. 北方习见植物. 河南科学技术出版社, 郑州.
1365. 叶育石, 易绮斐, 曾飞燕, 付琳. 2011. 广州植物志新资料. 热带亚热带植物学报. 19(5): 480-482.
1366. 易富科主编. 2008. 中国东北湿地野生维管束植物 (上)册. 科学出版社, 北京.
1367. 易富科主编. 2008. 中国东北湿地野生维管束植物 (下)册. 科学出版社, 北京.
1368. 易同培, 蒋学礼, 唐海倬, 李志宏. 2011. 四川方竹属一新种. 四川林业科技. 32(1): 11-13.
1369. 易同培. 1998. 四川植物志 12卷. 四川民族出版社, 成都.
1370. 应俊生,张玉龙. 1994. 中国种子植物特有属. 科学出版社, 北京.
1371. 应俊生. 2001. 中国植物志 29卷. 科学出版社, 北京.
1372. 游以德,陈玉峰,吴盈著. 1990. 台湾原生植物 (上)册. 淑馨出版社, 台北.
1373. 游以德,陈玉峰,吴盈著. 1990. 台湾原生植物 (下)册. 淑馨出版社, 台北.
1374. 于景华, 原树生, 佟露. 2010. 内蒙古大兴安岭白头翁属一新变种. 植物研究. 30(6): 648.
1375. 于胜祥, 许为斌, 陈艺林, 覃海宁. 2010. 海南凤仙花与龙州凤仙花花的形态补充描述. 广西植物. 30(1): 33-35.
1376. 于延球. 2008. 江苏沿海地区原色种子植物志. 科学出版社, 北京.
1377. 于兆英,徐光远,刘亮,崔建丽,徐养鹏. 1988. 中国滩羊区植物志 1卷. 宁夏人民出版社, 银川.
1378. 余治家,赵世华,袁彩霞. 2008. 六盘山主要野生木本观赏植物. 宁夏人民出版社, 银川.
1379. 余作岳,彭少麟主编. 1996. 热带亚热带退化生态系统植被恢复生态学研究. 广东科学技术出版社, 广州.
1380. 俞德浚. 1974. 中国植物志 36卷. 科学出版社, 北京.
1381. 俞德浚. 1985. 中国植物志 37卷. 科学出版社, 北京.
1382. 俞德浚. 1986. 中国植物志 38卷. 科学出版社, 北京.
1383. 俞德浚. 1986. 中国植物志 3卷. 科学出版社, 北京.
1384. 喻理飞,李明晶,谢双喜. 2000. 贵州佛顶山自然保护区科学考察集. 中国林业出版社, 北京.
1385. 喻理飞,谢双喜,吴太伦. 2004. 宽阔水自然保护区综合科学考察集. 贵州科学技术出版社, 贵阳.
1386. 喻庆国,钱德仁主编. 2006. 小黑山自然保护区. 云南科学技术出版社, 昆明.
1387. 喻勋林,肖育檀. 1999. 湖南石灰岩特有植物的初步研究. 中南林学院学报. 19(2):34-38.
1388. 袁家谟,陈训,先静缄. 1990. 贵州芳香植物. 贵州科学技术出版社, 贵阳.
1389. 袁明,王慷林,普迎冬. 2005. 云南德宏傣族景颇族自治州竹亚科(禾本科)植物区系地理研究. 云南植物研究. 27(1):19-26.
1390. 袁永孝,宋朝枢. 1998. 白石砬子自然保护区科学考察集. 中国林业出版社, 北京.
1391. 云南大学生态学与地植物学研究所,昭通行署林业局,昭通市林业局. 2001. 云南大山包黑颈鹤自然保护区科学考察报告集. 内部资料未正式出版.
1392. 云南省林业调查规划设计院昆明分院,云南省曲靖市林业局. 2000. 云南珠江源自然保护区综合考察报告. 内部资料未正式出版.
1393. 云南省林业厅野生动植物保护办公室. 1996. 云南无量山自然保护区科学考察报告. 内部资料未正式出版.
1394. 云南省植物研究所编著. 1973. 云南经济植物. 云南人民出版社, 昆明.
1395. 云南师范大学西南林学院. 2007. 云南云龙天池自然保护区综合科学考察报告. 云南师范大学西南林学院.
1396. 臧得奎,樊金会. 1994. 山东省特有植物的研究. 植物研究. 14(1):48-58.
1397. 臧得奎. 1999. 鼠李属一新种. 植物研究. 19(4):381-373.
1398. 曾桂芳. 1996. 中国植物志 79卷. 科学出版社, 北京.
1399. 曾庆波,李意德,陈步峰,周光益,吴仲民主编. 1995. 海南岛尖峰岭地区生物物种名录. 中国林业出版社, 北京.
1400. 曾宪锋, 邱贺媛. 2010. 广东省野生植物一新记录科——蜡梅科. 西北植物学报. 30(1): 205-207.
1401. 曾宪锋. 2004. 秦皇岛植物区系地理. 中国农业科学技术出版社, 北京.
1402. 湛盛田主编. 2003. 黑龙江宝清七星河湿自然保护区植物志. 东北林业大学印刷厂.
1403. 张爱春,刘国荣. 2004. 赤峰植物. 内蒙古科学技术出版社, 赤峰.
1404. 张斌,李盛湖,肖笃志. 1991. 西藏阿里土地资源. 中国农业科学技术出版社, 北京.
1405. 张朝芳,章绍尧主编 1993. 浙江植物志 1卷. 浙江科学技术出版社, 杭州.
1406. 张承芸,花立民. 2008. 景泰高等植物资源. 甘肃科学技术出版社, 兰州.
1407. 张方钢. 2010. 天目山植物志 3卷. 浙江大学出版社, 杭州.
1408. 张芬耀, 陈征海, 谢文远, 吴棣飞, 马丹丹, 李根有. 2010. 浙江植物新资料. 西北植物学报. 30(11): 2340-2342.
1409. 张芬耀, 陈征海, 叶喜阳, 马丹丹, 李根有. 2010. 浙江省苦苣苔科一新记录属——全唇苣苔属. 热带亚热带植物学报. 18(4): 403-404.
1410. 张凤臣,杨兴中,李登武主编. 2006. 陕西韩城黄龙山褐马鸡自然保护区综合科学考察报告. 陕西科学技术出版社, 西安.
1411. 张光富. 2007. 安徽板桥自然保护区植物多样性. 南京师范大学出版社, 南京.
1412. 张贵一,于丽杰,王凤春著. 1996. 中国东北菊科蒿属专志. 东北林业大学出版社, 哈尔滨.
1413. 张桂萍,何平,张峰. 2002. 我国特有植物缙云卫矛同工酶变异的数量分析. 广西植物. 22(6):523-528.
1414. 张国兴,唐廷贵主编. 1991. 天津蓟县山区野生生物资源及开发利用. 海洋出版社, 北京.
1415. 张国珍,杨道德主编. 2004. 湖南壶瓶山国家级自然保护区科学考察报告集. 湖南科学技术出版社, 长沙.
1416. 张海浪,刘国云,袁正科主编. 2003. 湖南省通道县龙底沟谷森林生态系统自然保护区自然资源研究. 湖南科学技术出版社, 长沙.
1417. 张宏达,陈桂珠,刘治平,张社尧. 1998. 深圳福田红树林湿地生态系统研究. 广东科学技术出版社, 广州.
1418. 张宏达. 1979. 中国植物志 35(2)卷(册). 科学出版社, 北京.
1419. 张宏达. 1989. 中国植物志 49(1)卷(册). 科学出版社, 北京.
1420. 张宏达. 1998. 中国植物志 49(3)卷(册). 科学出版社, 北京.
1421. 张宏达等主编. 1998. 深圳福田红树林鸟类自然保护区植物名录. 广东科学技术出版社, 广州.
1422. 张洪江,杜士才主编. 2010. 重庆四面山森林植物群落及其土壤保持与水文生态功能. 科学出版社, 北京.
1423. 张华海,李明晶,邓锦光. 2006. 黎平太平山自然保护区综合科学考察集. 贵州科学技术出版社, 贵阳.
1424. 张华海,龙启德,廖德平主编. 2006. 兴义坡岗自然保护区综合科学考察集. 贵州科学技术出版社, 贵阳.
1425. 张华海,周庆,张金国主编. 2006. 湄潭百面水自然保护区综合科学考察集. 贵州科学技术出版社, 贵阳.
1426. 张华海主编. 2000. 贵州野生珍贵植物资源. 中国林业出版社, 北京.
1427. 张华海主编. 2003. 老蛇冲自然保护区科学考察集. 贵州科学技术出版社, 贵阳.
1428. 张华海主编. 2003. 南宫自然保护区科学考察集. 贵州科学技术出版社, 贵阳.
1429. 张建军. 2009. 甘肃小陇山国家级自然保护区生物多样性. 北京出版社, 北京.
1430. 张建勇,刘仲健. 2001. 彩云兜兰及其近缘种的研究. 植物分类学报. 39(6):562-567.
1431. 张剑,周虹,纪少波,张清,马锦星. 2007. 湖北黄石药用植物彩色图谱. 中医古籍出版社, 北京.
1432. 张金泉,徐颂军,项颂信,黄少敏,李协文,庄志成,朱自相,翁抗美,蓝锦欣,麦荣臻. 2004. 广东古兜山自然保护区总体规划. 中国林业出版社, 北京.
1433. 张金泉. 1991. 广东阴那山自然保护区植物及旅游地理. 广东教育出版社, 广州.
1434. 张君义主编. 1991. 吉林省野生经济植物图鉴. 吉林科学技术出版社, 长春.
1435. 张俊朴,吴红臣,朱学文,马宜品主编. 1996. 濮阳植物志. 中国科学技术出版社, 北京.
1436. 张良实主编. 2006. 云南轿子山自然保护区. 云南科学技术出版社, 昆明.
1437. 张美珍,赖明洲. 1993. 华东五省一市植物名录. 上海科学普及出版社, 上海.
1438. 张美珍,邱莲卿. 1992. 中国植物志 61卷. 科学出版社, 北京.
1439. 张明海,张勇,孙立新. 2009. 辽宁蛇岛老铁山自然保护区综合科学考察报告.
1440. 张荣京, 陈红锋, 叶育石, 吴世捷, 邢福武, 王发国. 2010. 海南鹦哥岭的种子植物资源. 华南农业大学学报. 31(3): 116-118.
1441. 张胜邦,董旭. 2004. 青海柴达木梭梭林自然保护区科学考察集. 中国林业出版社, 北京.
1442. 张寿洲,李沛琼. 2008. 深圳野生植物名录. 中国林业出版社, 北京.
1443. 张书东,王红,李德铢著. 2008. 滇东北巧家药山种子植物名录. 云南科学技术出版社, 昆明.
1444. 张书理,韩力峰,王国文主编. 2000. 大黑山自然保护区综合科学考察集. 大黑山自然保护区管理局,敖汉旗环境保护局.
1445. 张树仁. 2009. 中国常见湿地植物. 科学出版社, 北京.
1446. 张玺玉,陈安绪,王葆劳,李静甫,郑鸿波,骆增福. 1985. 华山药物志. 陕西科学技术出版社, 西安.
1447. 张喜祥,吴建平主编. 2003. 三江自然保护区自然资源研究. 东北林业大学出版社, 哈尔滨.
1448. 张宪春. 2004. 中国植物志 6(3)卷(册). 科学出版社, 北京.
1449. 张晓台,滕崇德编著. 1960. 山西野生植物. 山西人民出版社, 太原.
1450. 张秀实,吴征镒. 1998. 中国植物志 23(1)卷(册). 科学出版社, 北京.
1451. 张秀实. 1986. 贵州植物志 3卷. 贵州人民出版社, 贵阳.
1452. 张秀实. 1989. 贵州植物志 4卷. 四川民族出版社, 成都.
1453. 张耀甲,彭泽祥. 1998. 白龙江流域珍稀、特有植物的多样性及其保护. 甘肃科学学报. 10(2):15-19.
1454. 张掖市人民政府,兰州大学生命科学学院. 2008. 甘肃张掖黑河湿地自然保护区综合科学考察报告.
1455. 张义文. 2005. 南大港湿地保护研究. 西安地图出版社, 西安.
1456. 张勇,刘贤德,李鹏,李彩霞. 2001. 甘肃河西地区维管植物检索表. 兰州大学出版社, 兰州.
1457. 张跃进, 季景玉. 2010. 天麻属(兰科)一新变种——卵果天麻. 西北植物学报. 30(6): 1277-1278.
1458. 张云霞, 朱长山. 2011. 卢氏凤仙花(Impatiens lushiensis Y.L.Chen)的新模式指定. 植物科学学报. 29(5): 652-653.
1459. 张泽荣. 1988. 四川植物志 4卷. 四川科学技术出版社, 成都.
1460. 张炤玒,郎益贵,董福辰. 1992. 沂山植物. 山东大学出版社, 济南.
1461. 张志勇, 张玉斌, 刘晓娟, 闫成才, 王煜明, 董万涛, 付红彦, 马有旭, 余海正, 孙学刚. 2011. 甘肃省被子植物8个新记录种. 甘肃农业大学学报. 46(5): 77-80.
1462. 张自学,曹江营,阿荣. 1988. 科尔沁自然保护区-湿地、鸟类、植物资源. 内蒙古自治区环境保护科学研究所,内蒙古自治区环境监测中心站.
1463. 赵承春,李永新,文旭,易建文,周自臣,谢文芳,刘福荣,杨学锡,潘子秀,吴应华. 1993. 云南中药资源名录. 科学出版社, 北京.
1464. 赵厚涛, 税玉民. 2010. 石林石蝴蝶,中国云南苦苣苔科一新种. 云南植物研究. 32(4).
1465. 赵建成,孔照普主编. 2008. 河北木兰围场植物志 (上)卷. 科学出版社, 北京.
1466. 赵建成,孔照普主编. 2008. 河北木兰围场植物志 (下)卷. 科学出版社, 北京.
1467. 赵建成,马清温,郭晓莉主编. 2009. 北京地区珍稀濒危植物资源. 北京科学技术出版社, 北京.
1468. 赵建成,吴跃峰,关文兰. 2008. 河北驼梁自然保护区科学考察与生物多样性研究. 科学出版社, 北京.
1469. 赵俊,易祖盛,周先叶,肖智,张俊岭,徐宁,孙树刚,陈园,李扬,李继秋,岑竞仪,马婧,董俊,罗燕平,李强,孙晓瑜,蓝昭军,王忠明,卿小芳. 2010. 广州市水生动植物本底资源. 科学出版社, 北京.
1470. 赵可夫,李法曾. 1999. 中国盐生植物. 科学出版社, 北京.
1471. 赵利清, 郭柯, 朱相云, 陈宝瑞. 2011. 中国棘豆属(豆科)一新记录种——平卧棘豆. 植物科学学报. 31(2).
1472. 赵利清, 刘芳, 陈宝瑞. 2011. 内蒙古白头翁属一新变种. 西北植物学报. 31(10): 2131-2132.
1473. 赵利清. 2010. 内蒙古、宁夏维管植物新资料. 西北植物学报.30(3): 621-623.
1474. 赵能,刘军. 2002. 巴蜀地区的杨柳科植物(二). 四川林业科技. 23(3):1-13.
1475. 赵能,刘军. 2002. 巴蜀地区的杨柳科植物(一). 四川林业科技. 23(2):1-7.
1476. 赵能,周邦楷. 1985. 四川植物志 3卷. 四川科学技术出版社, 成都.
1477. 赵山虎,钱法文,宋永胜,于有忠主编. 2009. 内蒙古科尔沁国家级自然保护区综合科学考察. 云南科学技术出版社, 昆明.
1478. 赵文阁,谷金学. 2005. 黑龙江大沾河湿地自然保护区综合科学考察报告. 哈尔滨师范大学生命与环境科学学院黑龙江省沾河林业局.
1479. 赵延茂,宋朝枢主编. 1995. 黄河三角洲自然保护区科学考察集. 中国林业出版社, 北京.
1480. 赵一之,曹瑞. 1996. 内蒙古的特有植物. 内蒙古大学学报(自然科学版). 27(2):208-213.
1481. 赵一之,马玲. 2003. 鄂尔多斯黄鹌菜--黄鹌菜属一新种. 植物研究. 23(3):261-262.
1482. 赵一之,马玲. 2004. 中国黄鹌菜属一新种. 植物研究. 24(2):133-134.
1483. 赵一之,王铁娟. 2000. 关于蒙古绣线菊毛枝变种及回折绣线菊的学名订正. 植物研究. 20(3):257-259.
1484. 赵一之. 2002. 新疆婆婆纳属一种. 植物研究. 22(4):385
1485. 赵一之. 2005. 内蒙古大青山高等植物检索表. 内蒙古大学出版社, 呼和浩特.
1486. 赵毓棠主编. 2009. 吉林树木图志. 中国林业出版社, 北京.
1487. 赵运林,潘晓玲. 1996. 湘黔桂交界地区植物名录. 湖南科学技术出版社, 长沙.
1488. 赵运林,喻勋林,傅晓华,李顺祥,高志强,谢昭明. 2009. 湖南药用植物资源. 湖南科学技术出版社, 长沙.
1489. 赵之一. 1992. 内蒙古珍稀濒危植物图谱. 中国农业科学技术出版社, 北京.
1490. 赵之一. 2006. 鄂尔多斯高原维管植物. 内蒙古大学出版社, 呼和浩特.
1491. 哲里木盟林业处科左后旗林业局编印. 1980. 大青沟自然保护区. 内部资料未正式出版.
1492. 浙江省环境保护局编. 1994. 南麂列岛自然保护区综合考察文集. 中国环境出版社, 北京.
1493. 浙江省开化县林业局,古田山自然保护区. 1999. 浙江省古田山自然保护区动植物名录. 浙江省开化县林业局,古田山自然保护区管理处.
1494. 浙江省科学技术协会编. 1982. 九龙山自然资源综合科学考察报告. 浙江省科学技术协会, 杭州.
1495. 浙江省磐安县环境保护局. 2001. 浙江大盘山自然保护区自然资源综合考察报告. 浙江省磐安县环境保护局.
1496. 浙江省卫生厅主编. 1965. 浙江天目山药用植物志 (上)集. 浙江人民出版社, 杭州.
1497. 郑宝江,马建华. 2008. 扎龙湿地植物志. 东北林业大学出版社, 哈尔滨.
1498. 郑朝宗主编. 1993. 浙江植物志 6卷. 浙江科学技术出版社, 杭州.
1499. 郑成洋. 2003. 福建武夷山自然保护区珍稀、濒危和特有植物及其分布. 福建林业科技. 30(3):54-58.
1500. 郑光荣. 2009. 青海大通河流域高等植物名录. 青海人民出版社, 西宁.
1501. 郑惠章,李健,王守聪,郑宇编著. 2004. 西藏果树种质资源志. 中国农业出版社, 北京.
1502. 郑勉,闵天禄. 1980. 中国植物志 45(1)卷(册). 科学出版社, 北京.
1503. 郑万钧,傅立国. 1978. 中国植物志 7卷. 科学出版社, 北京.
1504. 郑万钧主编. 1983. 中国树木志 1卷. 中国林业出版社, 北京.
1505. 郑万钧主编. 1985. 中国树木志 2卷. 中国林业出版社, 北京.
1506. 郑万钧主编. 1997. 中国树木志 3卷. 中国林业出版社, 北京.
1507. 郑万钧主编. 2004. 中国树木志 4卷. 中国林业出版社, 北京.
1508. 郑艳,姚胜,张定成. 2000. 安徽景天属一新种. 植物研究. 20(3):246-247.
1509. 郑重,许天全. 1990. 湖北省珍稀特有植物及其分布概况. 环境科学与技术. 4:40-47.
1510. 郑重,赵子恩,付书遐,刘启宏,郑洁华,黄仁煌,王映明,倪学明,张树藩,高文本,汪前生,王诗云,龙颜贞,王宁珠,姜钟华. 1980. 神农架植物. 湖北人民出版社, 武汉.
1511. 中国科学院《中国自然地理》编辑委员会. 1983. 中国自然地理：植物地理 (上)册. 科学出版社, 北京.
1512. 中国科学院北京植物研究所. 1974. 中国高等植物图鉴 3册. 科学出版社, 北京.
1513. 中国科学院北京植物研究所. 1975. 中国高等植物图鉴 4册. 科学出版社, 北京.
1514. 中国科学院登山科学考察队主编. 1985. 天山托木尔峰地区的生物. 新疆人民出版社, 乌鲁木齐.
1515. 中国科学院华南植物研究所鼎湖山树木园. 1976. 鼎湖山植物手册. 肇庆新华印刷厂印刷.
1516. 中国科学院及甘肃省,冰川冻土沙漠研究所沙漠研究室编. 1973. 中国沙漠地区药用植物. 甘肃人民出版社, 兰州.
1517. 中国科学院昆明植物研究所,云南省林业厅,金平分水岭自然保护区管理局编著. 2000. 云南省金平分水岭自然保护区综合科学考察报告集. 内部资料未正式出版.
1518. 中国科学院林业土壤研究所,辽宁省商业厅,辽宁省林业厅,沈阳药学院编. 1960. 辽宁经济植物志. 辽宁人民出版社, 沈阳.
1519. 中国科学院内蒙古宁夏综合考察队. 1975. 内蒙古植被(续). 科学出版社, 北京.
1520. 中国科学院内蒙古宁夏综合考察队. 1985 内蒙古植被. 科学出版社, 北京.
1521. 中国科学院青藏高原综合科学考察队编,王文采主编. 1993. 横断山区维管植物 (上)册. 科学出版社, 北京.
1522. 中国科学院青藏高原综合科学考察队编,王文采主编. 1994. 横断山区维管植物 (下)册. 科学出版社, 北京.
1523. 中国科学院山西分院生物研究所主编. 1961. 山西省习见有毒植物(初稿). 内部资料未正式出版.
1524. 中国科学院陕西分院生物研究所,西北大学生物系编. 1960. 安康地区经济植物. 陕西人民出版社, 西安.
1525. 中国科学院沈阳应用生态研究所. 1998. 内蒙古白音敖包自然保护区综合科学考察报告集. 内蒙古白音敖包自然保护区.
1526. 中国科学院四川分院农业生物研究所主编. 1962. 四川野生经济植物志 (上)册. 四川人民出版社, 成都.
1527. 中国科学院四川分院农业生物研究所主编. 1963. 四川野生经济植物志 (下)册. 四川人民出版社, 成都.
1528. 中国科学院武汉植物研究所. 1979 湖北植物志 2卷. 湖北人民出版社, 武汉.
1529. 中国科学院武汉植物园. 1964. 湖北植物标本名录. 武汉市科学技术协会,中国科学院武汉植物园.
1530. 中国科学院武汉植物园. 2007. 中国科学院武汉植物园保育植物名录. 湖北科学技术出版社, 武汉.
1531. 中国科学院武汉植物园编. 1960. 湖北野生淀粉植物. 湖北人民出版社, 武汉.
1532. 中国科学院武汉植物园编. 1960. 湖北野生饲料植物. 湖北人民出版社, 武汉.
1533. 中国科学院武汉植物园编. 1960. 湖北野生纤维植物. 湖北人民出版社, 武汉.
1534. 中国科学院武汉植物园编. 1960. 湖北野生油料植物. 湖北人民出版社, 武汉.
1535. 中国科学院武汉植物园编. 1964. 鄂西草药名录. 湖北省地方国营新生印刷厂.
1536. 中国科学院西北生物土壤研究所植物组编. 1958. 黄河中游黄土区植物名录. 西安第一印刷厂印刷, 西安.
1537. 中国科学院西北植物研究所,兰州大学编著. 1977. 西北油脂植物. 陕西人民出版社, 西安.
1538. 中国科学院西北植物研究所编. 1974. 秦岭植物志 1(2)卷(册). 科学出版社, 北京.
1539. 中国科学院西北植物研究所编. 1976. 秦岭植物志 1(1)卷(册). 科学出版社, 北京.
1540. 中国科学院西北植物研究所编. 1978. 秦岭植物志 3(1)卷(册). 科学出版社, 北京.
1541. 中国科学院新疆综合考察队,中国科学院植物研究所主编. 1978. 新疆植被及其利用. 科学出版社, 北京.
1542. 中国科学院植物研究所,北京植物园编. 1959. 北京植物园栽培植物名录. 高等教育出版社, 北京.
1543. 中国科学院植物研究所,南京中山植物园编. 1959. 南京中山植物园栽培植物名录. 上海科学技术出版社, 上海.
1544. 中国科学院植物研究所,山西省生物研究所,山西省林业厅. 1987. 山西省关帝山庞泉沟自然保护区植被基本特征研究. 内部资料未正式出版.
1545. 中国科学院植物研究所,中国科学院西北植物研究所. 1974. 秦岭植物志 2(蕨类植物门)卷(册). 科学出版社, 北京.
1546. 中国科学院植物研究所主编. 1972. 中国高等植物图鉴 1册. 科学出版社, 北京.
1547. 中国科学院植物研究所主编. 1972. 中国高等植物图鉴 2册. 科学出版社, 北京.
1548. 中国科学院植物研究所主编. 1976. 中国高等植物图鉴 5册. 科学出版社, 北京.
1549. 中国科院西安植物园编. 1976. 西安植物园栽培植物名录. 陕西省汉中地区印刷厂.
1550. 中国林业科学研究院,青海林业局. 2001. 三江源自然保护区科学考察报告附录动植物名录. 内部资料未正式出版.
1551. 中国医学科学院药物研究所,北京医学院药学系,南京药学院,江苏植物研究所,卫生部药品检定所,中医研究院中药研究所,北京中医学院,上海第一医学院药学系,沈阳药学院药学系,云南省药品检定所,中国科学院上海药物研究所,浙江医科大学药学系,上海中医学院药学系. 1961. 中药志 3册. 人民卫生出版社, 北京.
1552. 中国医学科学院药物研究所,北京医学院药学系,南京药学院,江苏植物研究所,卫生部药品检定所,中医研究院中药研究所,北京中医学院,上海第一医学院药学系,沈阳药学院药学系,云南省药品检定所,中国科学院上海药物研究所,广州市药品检定所. 1959. 中药志 2册. 人民卫生出版社, 北京.
1553. 中国医学科学院药物研究所,北京医学院药学系,南京药学院,江苏植物研究所,卫生部药品生物制品检定所,中医研究院中药研究所,北京中医学院,上海第一医学院药学系,沈阳药学院药学系,云南省药品检定所,上海药物研究所,广州市药品检定所. 1959. 中药志 1册. 人民卫生出版社, 北京.
1554. 中国医学科学院药物植物资源开发研究所,中国医学科学院药物研究所,北京医科大学药学院,中国药科大学,江苏植物研究所,中国药品生物制品检定所,中国中医研究院中药研究所,北京中医学院,上海医科大学药学院,沈阳药学院药学系,云南省中医学院,浙江医科大学药学系,上海中医学院药学系,广州市药品检验所. 1994. 中药志 5册. 人民卫生出版社, 北京.
1555. 中华人民共和国商业部土产废品局,中国科学院植物研究所主编. 1961. 中国经济植物志 (上)册. 科学出版社, 北京.
1556. 中华人民共和国商业部土产废品局,中国科学院植物研究所主编. 1961. 中国经济植物志 (下)册. 科学出版社, 北京.
1557. 钟补求,杨汉碧. 1979. 中国植物志 67(2)卷(册). 科学出版社, 北京.
1558. 钟补求,郑斯绪,杨汉碧,黎兴江,金存礼,李沛琼. 1963. 中国植物志 68卷. 科学出版社, 北京.
1559. 钟补求. 1963. 中国植物志 6卷. 科学出版社, 北京.
1560. 钟章成,齐代华. 2009. 三峡库区消落带生物多样性与图谱. 西南师范大学出版社, 重庆.
1561. 重庆大风堡自然保护区科考组. 2005. 重庆大风堡自然保护区科学考察报告.
1562. 周邦楷. 1994. 四川植物志 11卷. 四川民族出版社, 成都.
1563. 周昌弘,彭镜毅,赵淑妙. 1987. 台湾植物资源与保育. 中华民国自然生态保育协会, 台北.
1564. 周杰, 陈俊愉. 2010. 中国菊属一新变种. 植物研究. 30(6).
1565. 周守标,郭新弧,秦卫华. 2004. 安徽紫薇属(千屈菜科)一新种. 植物研究. 24(4):392-393.
1566. 周守标,郭新弧,邵剑文. 2003. 山罗花属(玄参科)一新种. 植物研究. 23(3):263-265.
1567. 周守标,郭新弧. 2001. 黄芩属(唇形科)一新种. 植物研究. 21(4):504-505.
1568. 周太炎. 1987. 中国植物志 33卷. 科学出版社, 北京.
1569. 周伟,陈宝昆. 2010. 云南碧塔海自然保护区. 云南科学技术出版社, 昆明.
1570. 周繇,朱俊义,于俊林著. 2005. 中国长白山观赏植物彩色图志. 吉林教育出版社, 长春.
1571. 周繇. 2010. 中国长白山植物资源志. 中国林业出版社, 北京.
1572. 周以良,董世林,聂绍荃. 1986. 黑龙江树木志. 黑龙江科学技术出版社, 哈尔滨.
1573. 周以良,聂绍荃,袁晓颖,杨逢建主编. 2003. 黑龙江植物资源志. 东北林业大学出版社, 哈尔滨.
1574. 周以良主编. 1992. 黑龙江省植物志 4卷. 东北林业大学出版社, 哈尔滨.
1575. 周以良主编. 1992. 黑龙江省植物志 5卷. 东北林业大学出版社, 哈尔滨.
1576. 周以良主编. 1993. 黑龙江省植物志 11卷. 东北林业大学出版社, 哈尔滨.
1577. 周以良主编. 1998. 黑龙江省植物志 6卷. 东北林业大学出版社, 哈尔滨.
1578. 周以良主编. 1998. 黑龙江省植物志 9卷. 东北林业大学出版社, 哈尔滨.
1579. 周以良主编. 2001. 黑龙江省植物志 8卷. 东北林业大学出版社, 哈尔滨.
1580. 周以良主编. 2002. 黑龙江省植物志 10卷. 东北林业大学出版社, 哈尔滨.
1581. 周以良主编. 2003. 黑龙江省植物志 7卷. 东北林业大学出版社, 哈尔滨.
1582. 周印锁,彭泽祥. 1992. 甘肃远志属一新种. 植物研究. 12(4):363-365.
1583. 周政贤,姚茂森. 1989. 雷公山自然保护区科学考察集. 贵州人民出版社, 贵阳.
1584. 周重想. 2006. 二蹬岩自然保护小区植物多样性及其对水电工程建设响应. 华中农业大学(硕士论文).
1585. 朱佰江,张红,张海军,苏香玲,李岩,任步远,牛亚林,刘浦江. 2001. 托木尔峰自然保护区综合考察报告. 新疆林业勘察设计院.
1586. 朱长山,李贺敏,许永安. 2002. 中国繁缕(Stellaria chinensis Regel)一新变种. 植物研究. 22(4):390
1587. 朱棣原著,周自恒编著. 2008. 中国的野菜. 南海出版公司, 海口.
1588. 朱华,王洪,李保贵. 1998. 云南山胡椒属一新变种. 云南植物研究. 20(1):32-32.
1589. 朱华. 1996. 西双版纳石灰岩森林及植物多样性研究. 中国科学院西双版纳热带植物园.
1590. 朱华. 2000. 西双版纳龙脑香热带雨林生态学与生物地理学研究. 云南科学技术出版社, 昆明.
1591. 朱强, 李志刚, 宋景景, 王俊. 2011. 假水生龙胆一新变种——白花假水生龙胆. 西北植物学报. 31(10).
1592. 朱强, 王鸿, 田英, 王俊. 2010. 宁夏被子植物2个新记录种. 西北植物学报. 30(12).
1593. 朱仁斌, 程积民. 2011. 宁夏4种新记录植物. 西北植物学报. 31(11).
1594. 朱石麟,马乃训,傅懋毅主编. 1994. 中国竹类植物图志. 中国林业出版社, 北京.
1595. 朱守谦主编. 1993. 喀斯特森林生态研究 I. 贵州科学技术出版社, 贵阳.
1596. 朱太平,刘亮,朱明. 2007. 中国资源植物. 科学出版社, 北京.
1597. 朱维明. 1999. 中国植物志 3(2)卷(册). 科学出版社, 北京.
1598. 朱维明. 2006. 云南植物志 20卷. 科学出版社, 北京.
1599. 朱文中,周立志主编. 2010. 安庆沿江湖泊湿地生物多样性及其保护与管理. 合肥工业大学出版社, 合肥.
1600. 朱亚民主编. 1989. 内蒙古植物药志 2卷. 内蒙古人民出版社, 呼和浩特.
1601. 朱亚民主编. 1989. 内蒙古植物药志 3卷. 内蒙古人民出版社, 呼和浩特.
1602. 朱亚民主编. 2000. 内蒙古植物药志 1卷. 内蒙古人民出版社, 呼和浩特.
1603. 朱英群,谢兰禹,许继法,吴宝利,梁子山,张玉芳. 2002. 山东荚蒾属一新变种. 植物研究. 22(3):261
1604. 朱英群,臧得奎. 1998. 山东植物两新变种. 植物研究. 18(1):9
1605. 朱有昌. 1989. 东北药用植物. 黑龙江科学技术出版社, 哈尔滨.
1606. 朱兆泉,宋朝枢. 1999. 神农架自然保护区科学考察集. 中国林业出版社, 北京.
1607. 朱兆云主编. 2004. 云南天然药物图鉴 1卷. 云南科学技术出版社, 昆明.
1608. 朱宗元,梁存柱,王炜,刘钟龄. 2003. 紊蒿属一新种和对该属分类及演化的讨论. 植物研究. 23(2):147-153.
1609. 朱宗元,马毓泉,刘钟龄,赵一之. 1999. 阿拉善－鄂尔多斯生物多样性中心的特有植物和植物区系的性质. 干旱区资源与环境. 13(2):1-16.
1610. 祝正银, 闵伯清, 王秋玲. 2011. 唇形科鼠尾草属新植物. 植物研究. 31(1).
1611. 祝正银, 闵伯清, 祝世杰. 2011. 四川天南星属(天南星科)一新种——川西南星. 广西植物. 31(5): 572-574.
1612. 祝正银, 祝世杰. 2011. 峨眉山鸡矢藤属(茜草科)二新种. 植物研究. 31(4).
1613. 祝正银,闵伯清. 2000. 峨眉山天南星属一新种. 植物研究. 20(4):5-6.
1614. 祝正银,张士良. 2001. 峨眉山蕺菜属药用植物一新种. 植物研究. 21(1):1-2.
1615. 祝正银. 2004. 峨眉山旋花科植物一新种. 植物研究. 24(3):257-258.
1616. 庄凯勋. 2008. 大兴安岭东部林区湿地资源. 东北林业大学出版社, 哈尔滨.
1617. 庄平,刘健,王云龙,张涛,陈锦辉. 2009. 长江口中华鲟自然保护区科学考察与综合管理. 海洋出版社, 北京.
1618. 庄璇. 2004. 云南植物志 13卷. 科学出版社, 北京.
1619. 訾兴中,张定成. 2009. 大别山植物志. 中国林业出版社, 北京.
1620. 訾兴中. 1999. 琅琊山植物志. 中国林业出版社, 北京.
1621. 邹天才. 2001. 贵州特有及稀有种子植物. 贵州科学技术出版社, 贵阳.
1622. 左家哺. 1998. 南岳森林生物多样性研究. 中国林业出版社, 北京.

Herbarium

The below list includes 37 herbarium and their cities across China.

| Code | Herbarium name | City |
| --- | --- | --- |
| 1 | Herbarium, Institute of Botany, Chinese Academy of Sciences | Beijing |
| 2 | Herbarium, South China Botanical Garden, Chinese Academy of Sciences | Guangzhou |
| 3 | Herbarium, Kunming Institute of Botany, Chinese Academy of Sciences | Kunming |
| 4 | Herbarium, Northwest Institute of Botany, Shaanxi Province and Chinese Academy of Sciences | Xianyang |
| 5 | Herbarium, College of Life Sciences Sichuan University | Chengdu |
| 6 | Chinese Field Herbarium | Beijing |
| 7 | Herbarium, Institute of Botany, Jiangsu Province and Chinese Academy of Sciences | Nanjing |
| 8 | Herbarium, Institute of Botany, Guangxi Zhuangzu Anonimimous Region and Chinese Academy of Sciences | Nanning |
| 9 | Herbarium, Lushan Botanical Garden, Jiangxi Province and Chinese Academy of Sciences | Jiujiang |
| 10 | Herbarium, Chengdu Institute of Biology, Chinese Academy of Sciences | Chengdu |
| 11 | The Qinghai-Tibet Plateau Biological Herbarium, Northwest Institute of Plateau Biology, Chinese Academy of Sciences | Lasha |
| 12 | Herbarium, Wuhan Botanical Garden, Chinese Academy of Sciences | Wuhan |
| 13 | Botanical Herbarium, School of Life Sciences, Nanjing University | Nanjing |
| 14 | Herbarium, Northwest Institute of Plateau Biology, Chinese Academy of Sciences | Xining |
| 15 | The Herbarium of Northeast China, Institute of Applied Ecology, Chinese Academy of Sciences | Shenyang |
| 16 | Herbarium, Xinjiang Institute of Ecology and Geography, Chinese Academy of Sciences | Urumqi |
| 17 | Botanical Herbarium, School of Life Sciences, Fudan University | Shanghai |
| 18 | Herbarium, Xishuangbanna Tropical Botanical Garden, Chinese Academy of Sciences | Menglun |
| 19 | Botanical Herbarium, Lanzhou University | Lanzhou |
| 20 | Botanical Herbarium, School of Life Sciences, Sun Yat-sen University | Guangzhou |
| 21 | Herbarium, Guangxi Institute of Chinese Medicine & Pharmaceutical Science | Nanning |
| 22 | Botanical Herbarium, School of Life Sciences, Inner Mongolia University | Hohhot |
| 23 | Botanical Herbarium, Zhejiang A & F University | Lin'an |
| 24 | Botanical Herbarium, Jiangxi Biotech Vocational College | Nanchang |
| 25 | Herbarium, West China Subalpine Botanical Garden, Institute of Botany, Chinese Academy of Sciences | Dujiangyan |
| 26 | Herbarium, Hangzhou Botanical Garden | Hangzhou |
| 27 | Botanical Herbarium, Institute of Biology, Guizhou Academy of Sciences | Guiyang |
| 28 | Forestry College, Northeast Forestry University | Harbin |
| 29 | Herbarium, Hunan University of Science and Technology | Xiangtan |
| 30 | Botanical Herbarium, School of Life Sciences, Xinjiang University | Urumqi |
| 31 | Botanical Herbarium, College of Life Sciences Henan Normal University | Xinxiang |
| 32 | Botanical Herbarium, College of Life Sciences Nankai University | Tianjin |
| 33 | Museum of Traditional Chinese Medicine, Chengdu University of Traditional Chinese Medicine | Chengdu |
| 34 | Botanical Herbarium, College of Life and Envrionment Science Hangzhou Normal University | Hangzhou |
| 35 | Botanical Herbarium, Southwest Forestry University | Kunming |
| 36 | Botanical Herbarium, Xinjiang Agricultural University | Urumqi |
| 37 | Botanical Herbarium, School of Life Sciences, East China Normal University | Shanghai |
